# Nonmonotone invasion landscape by noise-aware control of metastasis activator levels

**DOI:** 10.1038/s41589-023-01344-z

**Published:** 2023-05-25

**Authors:** Yiming Wan, Joseph Cohen, Mariola Szenk, Kevin S. Farquhar, Damiano Coraci, Rafał Krzysztoń, Joshua Azukas, Nicholas Van Nest, Alex Smashnov, Yi-Jye Chern, Daniela De Martino, Long Chi Nguyen, Harold Bien, Jose Javier Bravo-Cordero, Chia-Hsin Chan, Marsha Rich Rosner, Gábor Balázsi

**Affiliations:** 1 Department of Biomedical Engineering, Stony Brook University, Stony Brook, NY USA; 2 Louis and Beatrice Laufer Center for Physical and Quantitative Biology, Stony Brook University, Stony Brook, NY USA; 3 Genetics and Epigenetics Graduate Program, The University of Texas MD Anderson Cancer Center, UT Health Graduate School of Biomedical Sciences, Houston, TX USA; 4 Department of Pharmacological Sciences, Stony Brook University, Stony Brook, NY USA; 5 Department of Molecular and Cellular Biology, Roswell Park Cancer Institute, Buffalo, NY USA; 6 Department of Medicine, Division of Hematology and Oncology, Tisch Cancer Institute, Icahn School of Medicine at Mount Sinai, New York, NY USA; 7 Ben May Department for Cancer Research, University of Chicago, Chicago, IL USA; 8 Stony Brook Cancer Center, Stony Brook University, Stony Brook, NY USA

**Keywords:** Synthetic biology, Target validation, Cancer therapy

## Abstract

A major pharmacological assumption is that lowering disease-promoting protein levels is generally beneficial. For example, inhibiting metastasis activator BACH1 is proposed to decrease cancer metastases. Testing such assumptions requires approaches to measure disease phenotypes while precisely adjusting disease-promoting protein levels. Here we developed a two-step strategy to integrate protein-level tuning, noise-aware synthetic gene circuits into a well-defined human genomic safe harbor locus. Unexpectedly, engineered MDA-MB-231 metastatic human breast cancer cells become more, then less and then more invasive as we tune BACH1 levels up, irrespective of the native BACH1. BACH1 expression shifts in invading cells, and expression of BACH1ʼs transcriptional targets confirm BACH1ʼs nonmonotone phenotypic and regulatory effects. Thus, chemical inhibition of BACH1 could have unwanted effects on invasion. Additionally, BACH1ʼs expression variability aids invasion at high BACH1 expression. Overall, precisely engineered, noise-aware protein-level control is necessary and important to unravel disease effects of genes to improve clinical drug efficacy.

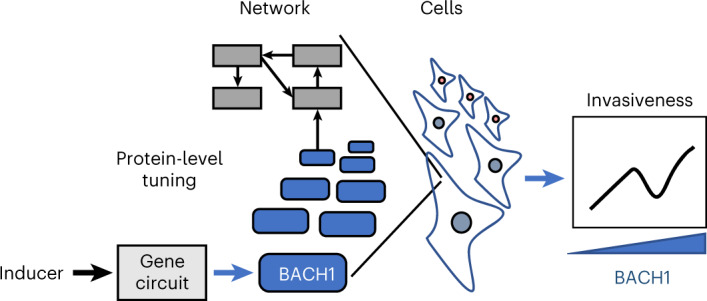

## Abstract (web summary)

Lowering the levels of disease-promoting proteins is generally assumed to be beneficial. The authors developed a two-step strategy to integrate protein-level tuning, noise-aware synthetic gene circuits into a well-defined human genomic locus. This approach was used to study the effect of BACH1 levels on MDA-MB-231 human breast metastatic cells.

## Main

Cancer is a process of cellular evolution, whereby cells exposed to oncogenic selection pressures develop characteristic hallmarks^1–3^. As in other evolutionary scenarios, cancer progression requires heritable variation within the cell population^4^, which, besides DNA alterations^5^, could also originate from epigenetic, transcriptomic^6^, proteomic^7^ or metabolomic^8^ variability^9,10^. Dividing cells can pass on their phenotypic differences^11,12^, allowing non-genetic evolution^13^. Protein levels can correlate closely with single-cell phenotypes that diversify due to genetic, microenvironmental or stochastic causes^14,15^ and shift due to regulatory responses^16^ or phenotypic selection^17,18^. Protein-level deviations can drive tumorigenesis, chemoresistance, immune evasion and metastasis^13,19,20^. Thus, to refine the view on how protein levels control single cells and cell populations, tools that can precisely tune not only protein levels but also protein variability in living cells are needed.

**Fig. 1:**
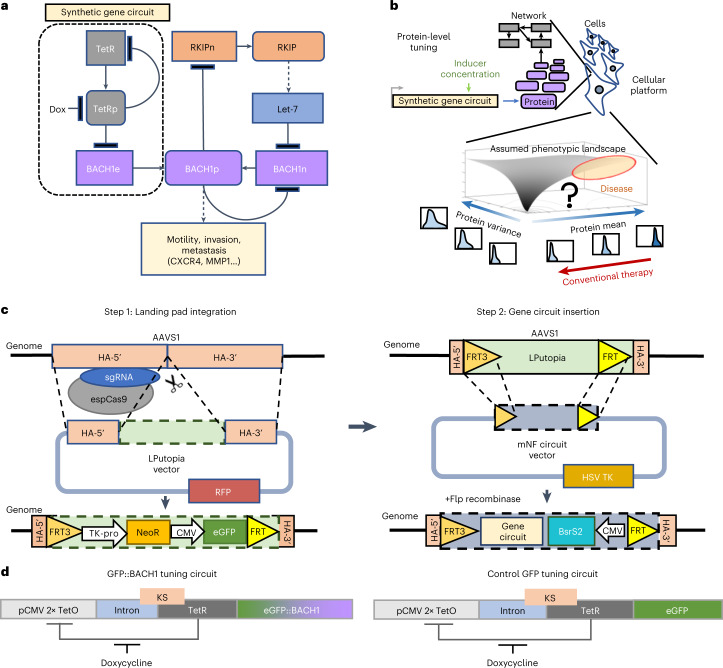
Two-step strategy for repeatable site-specific gene circuit integration. **a**, Network diagram of regulatory interactions and phenotypic impacts for native and ectopic BACH1. ‘BACH1e’ indicates the ectopic BACH1 gene introduced and controlled via the mNF gene circuit, whereas ‘BACH1n’ indicates the native BACH1 gene; ‘BACH1p’ indicates the BACH1 protein. The same notation applies to RKIP. **b**, A phenotype as a function of the mean and/or variability of one or more protein’s levels defines a phenotypic landscape. Conventional therapy assumes monotone phenotypic landscapes. However, uncovering the actual landscape requires protein-level tuning. **c**, Schematic diagram of the two-step strategy for repeatable AAVS1 site-specific integration of genetic payloads, such as synthetic gene circuits. Left: LP insertion into *AAVS1* with CRISPR–Cas9. Right: RMCE-based LP-specific integration of the mNF gene circuit that controls the expression of the eGFP::BACH1 bifunctional fusion. Step 2 is repeatable by using different selection markers. espCas9, enhanced specificity SpCas9; NeoR, neomycin resistance gene. **d**, Synthetic mNF gene circuit for dox-controlled tuning of the TetR co-expressed with either the GFP reporter (mNF-GFP) protein levels or GFP::BACH1 fusion (mNF-BACH1) protein levels after site-specific integration. KS, Kozak sequence; TetO, tetracycline operator.

Breast cancer is still a leading cause of mortality in women. Laboratory studies have benefitted from cell lines^21^ isolated from mammary tumors^22,23^ that are either mostly amenable for targeted therapy or multidrug-resistant, metastatic triple-negative breast cancers (TNBCs)^24^. Unlike for most primary tumors, the mutational bases of metastases are complex and ill-defined^25^. Instead, metastatic cells have altered cell population-level features, such as heterogeneous morphologies and transcriptomes^6,26^, with gene expression mean or heterogeneity perturbed by specific transcription factors, such as BTB and CNC homology 1 (BACH1). BACH1 is a metastasis-activator transcription factor^27,28^ that represses its own transcription as well as that of metastasis suppressor Raf kinase inhibitor protein (RKIP), also known as PE-binding protein-1 (PEBP1) (ref. ^29^) (Fig. 1a). Additionally, BACH1 activates multiple metastasis effector proteins such as CXCR4 and matrix metalloproteinase MMP1 (ref. ^30^). To design effective therapies targeting BACH1 in TNBC^31,32^, the phenotypic and regulatory consequences (‘landscape’; Fig. 1b) of altering both the mean and heterogeneity of cellular BACH1 levels should be established. For example, would metastatic behaviors always intensify with increasing BACH1 levels, as typically assumed for metastasis activators? Would increasing BACH1 levels always lower RKIP levels? What would happen upon altering BACH1 variance besides its average? Although these crucial questions are still open, therapeutic BACH1 inhibition is being suggested to diminish metastasis^31,32^, by the unverified paradigm that inhibiting oncogenes or other disease-promoting genes is generally beneficial—that is, their effect landscapes are monotone (Fig. 1b). However, such assumptions are risky without quantitative cellular phenotyping^33^ versus the fine-tuned mean and variance of drug-target protein levels^34,35^, initially in vitro, which necessitates genetic tools currently lacking in human cells.

Studying the phenotypic effects of proteins has benefitted from transient (plasmids) or stable (gene deletion or transgene integration) knockout/knockdown or overexpression approaches. However, all these perturbations suffer from two shortcomings. The first shortcoming is their qualitative character, which cannot define quantitatively either the ups and downs of regulatory and phenotypic effects at intermediate protein levels or the effects of protein-level variability. Synthetic biology is addressing this challenge using gene circuits to fine-tune protein-level mean and variability by chemicals or light^36–38^. Similar tools have generated cellular fitness landscapes in bacteria^39^ and yeast^40^ but not yet in mammalian studies. Alternatively, CRISPR methods based on DNA-binding mutated Cas^41^ or RNA-cleaving Cas variants^42^ have created either transient or randomly integrated transcript-controlling tools but without adjusting the variability. The second shortcoming is that genomic integration of expression control tools—for example, by lentiviral transduction^43^ or CRISPR–Cas^44^—can trigger unwanted, potentially risky off-target genetic and epigenetic alterations. Conversely, native molecular mechanisms, such as epigenetic silencing, can compromise transgene expression in human cells^45^. To overcome these drawbacks, site-specific recombinases (SSRs) targeting human safe harbor sites (SHSs)—that is, SSR/SHS—can minimize the mutual interference between transgenes and host cell^46^ because (1) SSR only interacts with its own recognition sites^47^; (2) altering SHS genetic sequences does not disrupt cell functions^46^; and (3) genes inserted into SHSs are well expressed, with modest silencing^46^. Developed and standardized in bacteria and yeast^48^, SSR/SHS is mostly restricted to a few commercial mammalian cell lines carrying SSR recognition sites in unknown genomic SHS loci^37^. Overall, combining synthetic gene circuit engineering with SSR/SHS could address both shortcomings, enabling disease-related fitness landscape mapping while adjusting both the mean and variance of protein levels.

As a step toward quantitatively unraveling BACH1ʼs role as a metastasis regulator in TNBC, we developed a generally applicable, two-step genome engineering strategy (Fig. 1c) to integrate synthetic gene circuits into the *AAVS1* (adeno-associated virus integration site 1) SHS of any human cell line. Using this method, we established clones with tunable mean and variance of BACH1 levels from the MDA-MB-231 TNBC cell line. Unexpectedly, we discovered a nonmonotone invasion landscape, with invasion increasing, decreasing and then increasing again as mean BACH1 levels increase in such cells in vitro. We confirmed this nonmonotone relationship by examining BACH1 expression distributions of invading versus seeded cells, which indicate directional or disruptive selection. Additionally, BACH1 expression noise aids invasion but only at high BACH1 expression. Moreover, the expression of multiple BACH1 transcriptional targets confirm nonmonotone BACH1 regulatory effects, with additional support from TNBC clinical samples and cell line data. Homozygous BACH1 deletion alters, but does not eliminate, the nonmonotone effects, excluding native BACH1 as the sole cause of nonmonotonicity. Taken together, we uncovered nonmonotone effects of BACH1 in TNBC cells, and here we demonstrate the need and synthetic biology-based possibility of phenotypic landscape mapping to quantitatively understand and control the complex effects of clinically relevant proteins.

## Results

### Two-step strategy for robust SHS-specific gene circuit integration

To develop a strategy for reliable single-copy gene circuit integration into *AAVS1* (ref. ^49^), which is the most prominent human SHS, we designed a two-step technical pipeline (Fig. 1c). First, we introduce an FRT landing pad (LP) into *AAVS1* by generating a double-strand break with CRISPR–Cas9. Second, we flip various gene circuits into the LP by homologous recombination without additional double-strand breaks^50^.

**Extended Data Fig. 1:**
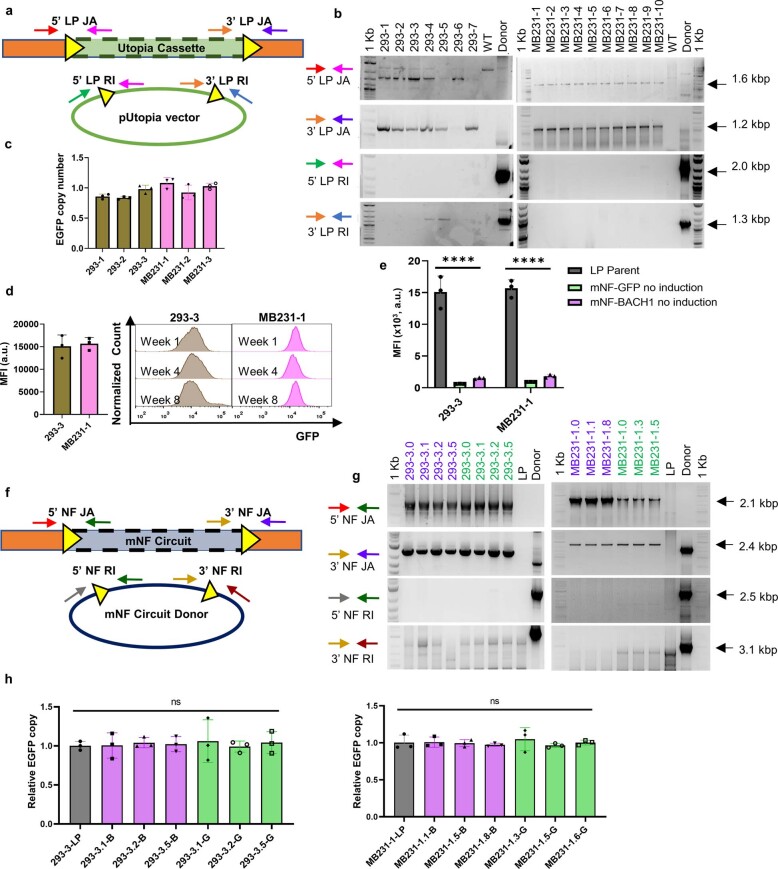
Validation of the integration specificity and quality of Landing Pad and mNF clones. **(a)** Strategy to validate the insertion specificity of LP cassette of each monoclonal sample via PCR genotyping. Primers were designed to amplify the adjunction (border) regions between the constructs and the genome at both the (5’ and 3’) ends of the inserts. The adjunction regions with the vector backbones were also tested for any possible random integration. Identical primers are indicated in the same color. **(b)** PCR genotyping of 293 and MB231 Landing Pad (LP) cell lines using primer sets depicted in **(a)** for 5’/3’ junction assay (JA) and random integration assay (RI). Parental cells (WT) and Donor plasmid were included as controls. **(c)** *EeGFP* copy number determination via qPCR of the selected 293 and MB231 LP cell lines. Each sample copy number was normalized to the reference gene *RPPH1* (n = 3). **(d)** Identical constitutive GFP expression of representative LP cell lines of 293 and MB231 over time (n = 3). **(e)** Mean fluorescence intensity (MFI) comparison between parental Landing Pad cells, versus uninduced mNF-GFP and mNF-BACH1 integrated cells. n = 3, One-way ANOVA, P < 0.0001. **(f)** Strategy to validate the insertion specificity of mNF circuits for each monoclonal sample via PCR genotyping. Primers were designed to amplify the adjunction regions between the constructs and the genome at both the (5’ and 3’) ends of the inserts. The adjunction regions with the vector backbones were also tested for any possible random integration. Identical primers are indicated in the same color. **(g)** PCR genotyping of the mNF-BACH1- (purple) and mNF-GFP- (green) integrated monoclonal populations and polyclonal populations (marked as clone 0) of 293-3 and MB231-1 using primer sets depicted in **(f)** for 5’/3’ junction assay (JA) and random integration assay (RI). Parental Landing Pad cells (LP) and mNF circuit donor plasmids were included as controls. **(h)** *eGFP* copy number determination via qPCR of the selected 293 **(left)** and MB231 **(right)** mNF-BACH1- (purple) and mNF-GFP- (green) integrated monoclonal populations relative to the corresponding parental Landing Pad population (LP, grey). Each sample was normalized to corresponding LP sample in each cell type. One-way ANOVA, n = 3. Source data

First, to create an LP, we designed the donor vector LPutopia (Fig. 1c), with selection markers and the cytomegalovirus (CMV) promoter-driven *eGFP* reporter between the Flp recombinase target sites FRT and FRT3, which were further flanked by two *AAVS1*-targeting homology arms (HAs). After inserting LPutopia with CRISPR–espCas9 into *AAVS1* in HEK293 and MDA-MB-231 cells, we characterized 10 MDA-MB-231 LP clones (MB231-1 through MB231-10) and seven HEK293 LP clones (293-1 through 293-7) (Supplementary Fig. 1a). We confirmed that all selected clones had stable *AAVS1*-specific, single-copy LP integration and were random integration-free with very few exceptions (Extended Data Fig. 1a–d).

Second, we integrated a mammalian negative feedback (mNF) synthetic gene circuit into LP clones MB231-1 and 293-3 by Flp^50^ recombinase-mediated cassette exchange (RMCE)^51^ without double-strand breaks^52^. These mNF gene circuits^53^ (Fig. 1d) rely on a self-controlling tetracycline repressor (TetR) for doxycycline (dox)-dependent, low-noise tuning of either the eGFP reporter (mNF-GFP) or a bifunctional^54^ eGFP::BACH1 fusion (mNF-BACH1). Using the low uninduced expression of mNF-integrant cells (Extended Data Fig. 1e), we enriched for these cells by double sorting, first GFP^low^ cells without dox and then GFP^high^ cells with 100 ng ml^−1^ dox induction (Supplementary Fig. 1b), followed by single-cell bottlenecking. Finally, we confirmed single-copy *AAVS1*- and LP-specific integration of the mNF gene circuits without random insertions even in polyclonal samples (Extended Data Fig. 1f–h).

In conclusion, through SSR/SHS, we generated and validated multiple monoclonal cell lines derived from 293-3 and MB231-1 LP clones, with either the mNF-GFP or the mNF-BACH1 gene circuit integrated into the LP in the *AAVS1* locus. Such precisely engineered cell lines are fundamentally necessary for quantitatively tuning target gene expression and studying the corresponding disease-related phenotypic landscapes.

### Fluorescence dose responses of LP-integrated mNF gene circuits

**Extended Data Fig. 2:**
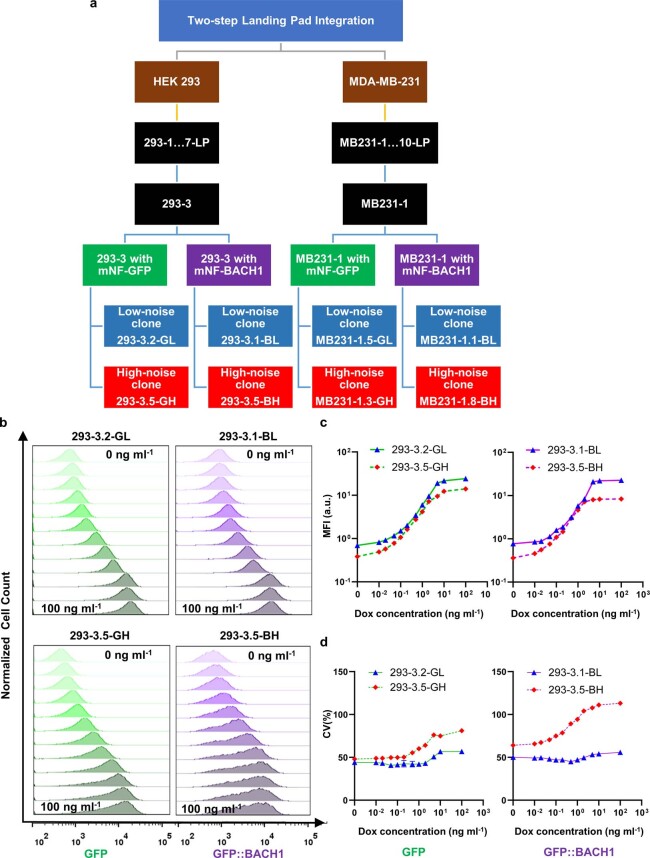
Cell line engineering scheme in MB231 and HEK293 cells, and flow cytometry quantitation of inducible expression in selected HEK293 clones. **(a)** Cell line engineering steps and corresponding annotations for different sets of engineered cells, indicating the chosen parental LP clones and corresponding mNF clones. Low- and high-noise clones of both mNF-GFP and mNF-BACH1 clones were labelled GL, GH and BL, BH, respectively. **(b)** Representative dose-responses of fluorescence intensity histograms from low-noise mNF-GFP (GL), mNF-BACH1 (BL) and high-noise mNF-GFP (GH), mNF-BACH1 (BH) 293 clones measured at 0, 0.01, 0.02, 0.05, 0.1, 0.2, 0.5, 1, 2, 5, 10, 100 ng ml^−^^1^ Dox levels, respectively. **(c)** Dose-responses of mean fluorescence intensity (MFI) for low-noise mNF-GFP (GL), mNF-BACH1 (BL) and high-noise mNF-GFP (GH), mNF-BACH1 (BH) 293 clones (n = 3). **(d)** Dose-responses of coefficient of variation (CV) from low-noise mNF-GFP (GL), mNF-BACH1 (BL) and high-noise mNF-GFP (GH), mNF-BACH1 (BH) 293 clones (n = 3). Source data

Phenotypic landscape mapping requires dose–response measurements to characterize gene expression tunability. To test mNF’s expression control capability, we first screened multiple mNF-BACH1 and mNF-GFP clones by measuring the fold change and variance of eGFP expression. Despite their identical descent and genetic background before gene circuit insertion, clones had substantial differences both in the expression fold change and cell–cell variability or noise, measured by the coefficient of variation (CV). We chose from each cell type one low-noise and one high-noise mNF clone with maximum noise difference but relatively similar fold change of mean expression (Extended Data Fig. 2a) for detailed dose–response characterization and subsequent phenotypic landscape mapping.

**Fig. 2:**
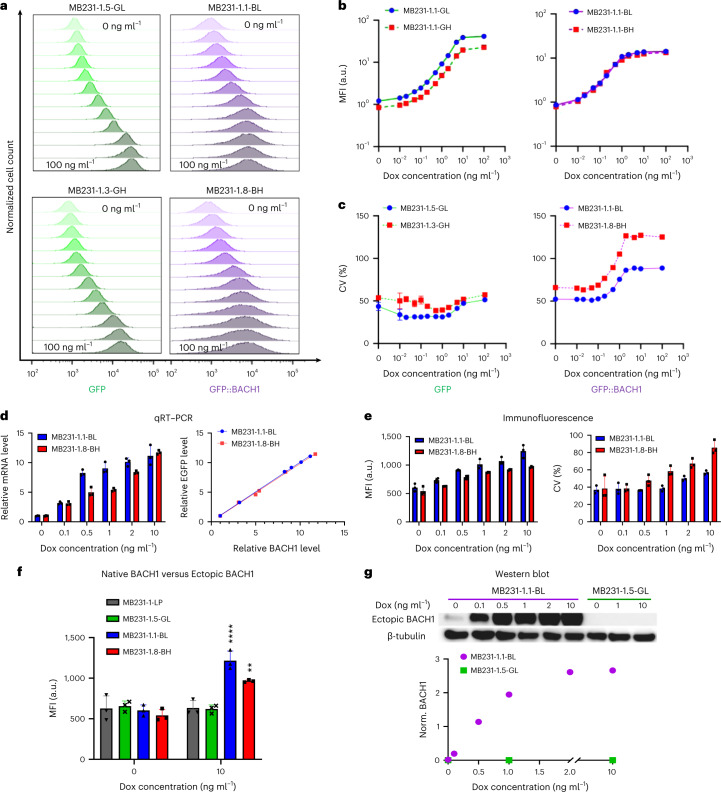
Protein-level and transcript-level dose responses of the mNF-BACH1 gene circuit, site-specifically integrated into AAVS1 in MB231 cells. **a**, Representative dose responses of fluorescence intensity histograms from low-noise mNF-GFP (GL), mNF-BACH1 (BL) and high-noise mNF-GFP (GH), mNF-BACH1 (BH) MB231 clones measured at 0, 0.01, 0.02, 0.05, 0.1, 0.2, 0.5, 1, 2, 5, 10 and 100 ng ml^−1^ dox levels, respectively. **b**, Dose responses of mean fluorescence intensity (MFI) for low-noise mNF-GFP (GL), mNF-BACH1 (BL) and high-noise mNF-GFP (GH), mNF-BACH1 (BH) MB231 clones (*n* = 3). **c**, Dose responses of CV for low-noise mNF-GFP (GL), mNF-BACH1 (BL) and high-noise mNF-GFP (GH), mNF-BACH1 (BH) MB231 clones (*n* = 3). **d**, Left: BACH1 mRNA level dose responses of both low-noise mNF-BACH1 (BL) and high-noise mNF-BACH1 (BH) MB231 clones. Relative mRNA levels were calculated between each individual replicate and the corresponding uninduced control (*n* = 3). Right: correlation between BACH1 and GFP mRNA levels in mNF-BACH1 clones (linear regression slopes of 0.9965 and 0.9778 for MB231-1.1-BL and MB231-1.8-BH, respectively; R^2^ goodness-of-fit values of 0.9992 and 0.9971 for MB231-1-BL and BH, respectively). **e**, Left: protein-level dose responses of total (endogenous + ectopic) BACH1 protein in both low-noise mNF-BACH1 (BL) and high-noise mNF-BACH1 (BH) MB231 clones (*n* = 3). Right: total BACH1 protein level noise assessed from immunofluorescence measurements for both low-noise mNF-BACH1 (BL) and high-noise mNF-BACH1 (BH) MB231 clones (*n* = 3). **f**, Comparison of total BACH1 protein level at uninduced (0 dox) and fully induced (10 ng ml^−1^ dox) conditions for low-noise mNF-BACH1 (BL) and high-noise mNF-BACH1 (BH) MB231 cell populations to native BACH1 protein level in low-noise mNF-GFP (GL) and their parental LP cell populations. *n* = 3, one-way ANOVA with Tukey’s multiple comparison correction at 0 dox and 10 µg ml^−1^ dox with respect to LP sample, **P < 0.01, ****P < 0.0001. **g**, Western blot examination and quantitation of ectopic BACH1 protein-level dose response in low-noise mNF-BACH1 and mNF-GFP MB231 samples. BACH1 levels were normalized to corresponding internal β-tubulin levels using grayscale quantitation. Source data

**Extended Data Fig. 3:**
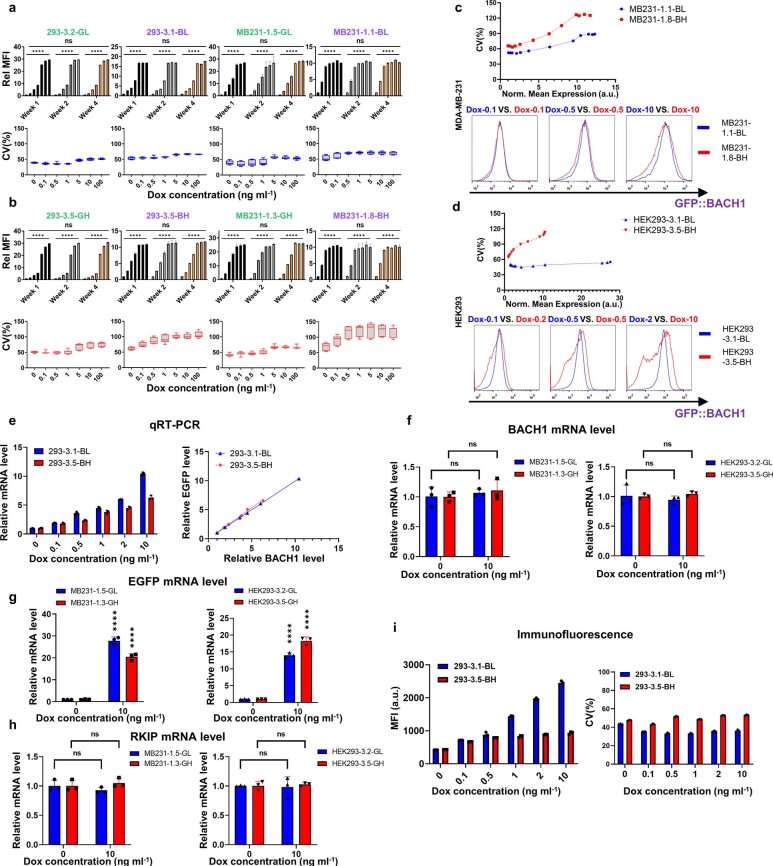
Reproducibility of inducible expression and BACH1-dependent downstream target changes and mean-noise decoupling in selected clones. **(a)**,**(b)** Dose-responses of mean fluorescence intensity (MFI) and CV at 0, 0.1, 0.5, 1, 5, 10, 100 ng ml^−^^1^ Dox concentrations over 4 weeks in low-noise **(a)** and high-noise **(b)** 293 and MB231 clones, respectively. Relative mean fluorescence intensity (Rel MFI) **(Top)** of each individual replicate of each clone was calculated via normalization to a single replicate at 0 ng ml^−^^1^ Dox concentration in the first week. Two-way ANOVA was performed to compare the differences between doses and time points. All time point comparisons had P-values over 0.5 and comparisons between doses had P-values below 0.0001, n = 3. Coefficient of variation (CV) **(Bottom)** of each clone at every dose condition was calculated from 3 independent measurements at first, second and fourth week with total 9 replicates pooled together. Two-tailed Mann Whitney test was performed to compare the CV differences between corresponding low- and high-noise clones at every dose. MB231-1.1-BL versus MB231-1.8-BH had a P-value of 0.0111 and MB231-1.5-GL versus MB231-1.3-GH had a P-value below 0.0111. 293-3.1-BL versus 293-3.5-BH had a P-value of 0.0041 and 293-3.2-GL versus 293-3.5-GH had a P-value below 0.0111, n = 9. For box-plot, center line represents the median and whiskers is from minimum to maximum. **(c)(d)** Plotting the noise (CV) as a function of normalized mean gene expression for both low- and high-noise clones of MB231 **(c)** and 293 **(d)** cells revealed broad decoupled noise regimes **(Top)** where the CVs differ while the means are similar. Gene expression profile comparison between two clones at representative decoupled noise points **(Bottom)**. **(e) Left panel:** BACH1 mRNA level dose-responses of both low-noise mNF-BACH1 (BL) and high-noise mNF-BACH1 (BH) 293 clones. Relative mRNA levels were calculated between each individual replicate and the corresponding uninduced control (n = 3). **Right panel:** Correlation between BACH1 and GFP mRNA levels in mNF-BACH1 clones (linear regression slopes of 0.9613 and 1.070 for 293-3.1-BL and 293-3.5-BH, respectively; R-square goodness of fit values of 0.9999, 0.9959 for 293-3-BL, BH, respectively). **(f) (g) (h)** BACH1, eGFP and RKIP mRNA expression in low- and high-noise mNF-GFP clones of both MB231 **(left)** and 293 **(right)** cells, unpaired two-tailed t-test, n = 3, P > 0.05. **(i) Left panel:** Protein-level dose-responses of total (endogenous + ectopic) BACH1 protein in both low-noise mNF-BACH1 (BL) and high-noise mNF-BACH1 (BH) 293 clones (n = 3). **Right panel:** Total BACH1 protein level noise assessed from immunofluorescence measurements for both low-noise mNF-BACH1 (BL) and high-noise mNF-BACH1 (BH) 293 clones (n = 3). Source data

To determine the full dose responses of all eight mNF clones (MB231-based and 293-based, high-noise and low-noise, mNF-BACH1 and mNF-GFP), we next examined the means and CVs of their gene expression after 48 h in constant dox concentrations ranging between 0 and 100 ng ml^−1^. Flow cytometry (Fig. 2a and Extended Data Fig. 2b) and fluorescence microscopy (Supplementary Figs. 2 and 3) consistently indicated a monotone dox-dependent, up to 30-fold increase of mean eGFP fluorescence intensity for every low-noise and high-noise clone (Fig. 2b,c and Extended Data Fig. 2c,d). The expression distributions of high-noise clones were broader, and their CV difference from low-noise clones increased with dox concentration (Fig. 2c and Extended Data Fig. 2d). These expression features of mNF clones were stable and reproducible in cell culture for up to 4 weeks (Extended Data Fig. 3a,b).

To later study how BACH1 expression noise impacts phenotypes, we sought to establish decoupled noise points with different gene expression noise but with similar mean expression^52,55^. Plotting the CV versus mean of expression revealed broad decoupled noise regimes for MB231 and 293 mNF clones (Extended Data Fig. 3c,d). Interestingly, the low-noise and high-noise MB231 mNF-BACH1 clones had significantly different CVs but nearly identical mean expression, making them suitable to test the phenotypic roles of noise independently of the mean.

To verify that the protein-level measurements reflect BACH1 mRNA expression, we examined BACH1 transcript levels via qRT–PCR. Total BACH1 mRNA levels increased in a monotone dox-dependent manner up to 11-fold in both low-noise and high-noise mNF-BACH1 293 and MB231 clones, and the eGFP and BACH1 mRNA levels correlated positively with slopes near 1 (Fig. 2d and Extended Data Fig. 3e). In mNF-GFP clones, only eGFP transcript levels increased, without significant changes in transcript levels of BACH1 or its direct downstream regulatory target RKIP (Extended Data Fig. 3f–h).

**Extended Data Fig. 4:**
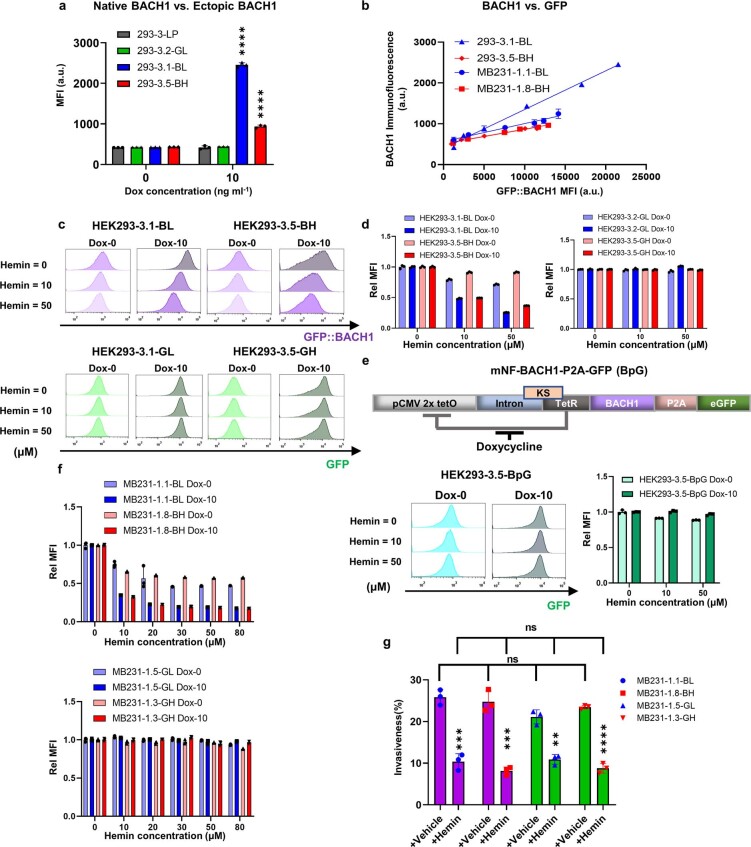
BACH1 protein level and cell invasion tuning via hemin treatment. **(a)** Comparison of total BACH1 protein level at uninduced (0 Dox) and fully-induced (10 ng ml^−^^1^ Dox) conditions for low-noise mNF-BACH1 (BL) and high-noise mNF-BACH1 (BH) 293 cell populations to native BACH1 protein level in low-noise mNF-GFP (GL) and their parental Landing Pad (LP) cell populations. n = 3, one-way ANOVA with Tukey’s multiple comparison correction at 0 and 10 ug/ml Dox with respect to LP sample, **P < 0.01, ****P < 0.0001. **(b)** Near-linear correlation between BACH1 immunofluorescence (IF) readout and GFP mean fluorescence intensity (MFI) (n = 3; R-square goodness of fit values for 293-3-BL, BH and MB231-1-BL, BH are 0.9905, 0.9571, 0.9026, 0.9615, respectively). **(c)** GFP fluorescence histogram at 0 and 10 ng ml^−^^1^ Dox shifts down upon hemin treatment for 48 hours in both 293 low- and high-noise mNF-BACH1 **(Top)** and mNF-GFP clones **(Bottom)**. **(d)** Relative changes of fluorescence intensity at 10 and 50 µM hemin concentration compared to no treatment control in both mNF-BACH1 **(left)** and mNF-GFP **(right)** 293 clones, n = 3. **(e)** Applying the same hemin treatment levels on the unfused BACH1 and GFP target revealed direct connection between BACH1 protein and GFP fluorescence intensity when they are fused, n = 3. **(f)** Relative changes of fluorescence intensity at 10, 20, 30, 50 and 80 µM hemin concentration compared to no treatment control in both mNF-BACH1 **(top)** and mNF-GFP **(bottom)** MB231 clones, n = 3. **(g)** Invasiveness universally drops over 50% in all selected MB231 clones upon 48 hours of 50 µM hemin treatment. Mean ± S.D. of n = 3 independent replicates. One-way ANOVA within each condition group, P > 0.05. Unpaired two-tailed t-test for each comparison between control and hemin treated sample, **P < 0.01, ***P < 0.001, ****P < 0.0001. Source data

In addition to the mNF-controlled, ectopic BACH1 copy, all clones contain native copies of the BACH1 gene. Using immunofluorescence to understand the dox dose response of overall BACH1 protein levels expressed from both native and ectopic BACH1 genes, we recapitulated monotone increases and noise differences for total BACH1 protein levels in all mNF-BACH1 293 and MB231 clones (Fig. 2e and Extended Data Fig. 3i). In mNF-GFP cells, total BACH1 protein levels were statistically indistinguishable from those of LP parental cells (Fig. 2f and Extended Data Fig. 4a), regardless of induction. In mNF-BACH1 cells, the eGFP reporter was an excellent indicator of BACH1 protein levels because its fluorescence correlated strongly with BACH1 immunofluorescence intensity (Extended Data Fig. 4b). Western blots further confirmed the monotone dox-dependent increase of ectopic BACH1 levels in the MB231 mNF-BACH1 clones and no change in mNF-GFP controls (Fig. 2g).

Moreover, we used hemin to test ubiquitin-mediated co-degradation of BACH1 (ref. ^56^) and eGFP reporter. Hemin caused a substantial reduction of eGFP fluorescence intensity in mNF-BACH1 cells (Extended Data Fig. 4c) but none in mNF-GFP cells. We also confirmed theoretical expectations of percent BACH1 reductions based on hemin-dependent but dox-independent rate constants of BACH1 degradation (Supplementary Notes 1.1 and Extended Data Fig. 4d). Finally, when we translationally separated BACH1 from eGFP in another SHS/SSR-generated 293 mNF clone, eGFP intensity decreased minimally upon hemin treatment (Extended Data Fig. 4e).

Overall, these findings indicate that dose responses may differ across mNF clones but remain stable and highly reproducible over time within each clone, supporting mNF as a protein expression-controlling device. The eGFP reporter co-exists and co-degrades with BACH1 via protein fusion, so eGFP fluorescence accurately reports BACH1 protein levels in single cells. The precise BACH1 tuning device that we created can interface with and deliver signals into the native BACH1 regulatory network^29,30^ (Fig. 1a), enabling quantitative exploration of phenotypic landscapes and network responses to tunable mean and variance of BACH1 levels.

### Noise-aware control shows that BACH1 nonmonotonically regulates cell invasion

As a master regulator^57^, BACH1 plays diverse roles in regulating multiple signaling and metabolic pathways, including its cancer metastasis activator function in TNBC. What exactly does the term ‘activator’ mean about the effect of BACH1 protein levels on metastasis—a highly complex evolutionary process requiring many steps, including cell migration, invasion, intravasation and extravasation, dissemination, colonization and metastatic outgrowth, each of which occurs with poorly measurable, low probabilities in vivo^58^? Owing to its complexity, quantitative studies of the entire metastatic process are currently unfeasible. Thus, to focus on a key aspect of BACH1ʼs metastasis activator function, we assayed invasion in vitro, which indicates metastatic potential^59^.

**Fig. 3:**
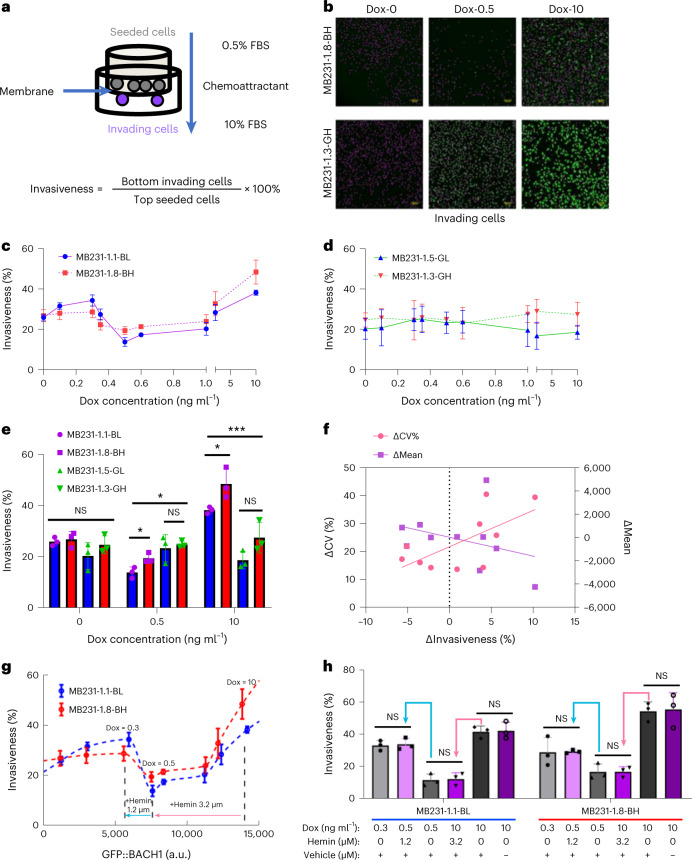
Nonmonotone BACH1 invasion landscape in engineered MDA-MB-231 cells. **a**, Experimental design for measuring and calculating invasiveness. **b**, Representative images of invading cells at the bottom of the inserts after 16 h–24 h invasion assays. Cell number was determined using nuclear staining and GFP fluorescence. Scale bar, 200 µm. **c**, Invasiveness of low-noise mNF-BACH1 (BL) and high-noise mNF-BACH1 (BH) clones over increasing dox concentrations (*n* = 3). **d**, Invasiveness of low-noise mNF-GFP (GL) and high-noise mNF-GFP (GH) clones at increasing dox concentrations (*n* = 3). **e**, Invasiveness at 0, 0.5 and 10 ng ml^−1^ dox levels for mNF-BACH1 versus mNF-GFP clones (*n* = 3, one-way ANOVA for cross-comparison among four samples at each dose and unpaired two-tailed *t*-test for every comparison between low-noise and high-noise clones. *P < 0.05, **P < 0.01, ***P < 0.001, ****P < 0.0001). **f**, Expression noise difference (ΔCV%, left *y* axis) and mean difference (ΔMean, right *y* axis) versus invasiveness difference (ΔInvasiveness) at different dox inductions between low-noise and high-noise mNF-BACH1 clones. Δ = BH − BL, *n* = 3, Pearson correlation coefficients for ΔCV% versus ΔInvasiveness, *r* = 0.6611, *P* = 0.0374; for ΔMean versus ΔInvasiveness, *r* = −0.3477, *P* = 0.3248. **g**, Invasiveness of low-noise mNF-BACH1 (BL) and high-noise mNF-BACH1 (BH) clones versus eGFP::BACH1 expression (fluorescence) level. Pink and cyan arrows indicate hemin concentrations needed to degrade BACH1 levels along an upslope, at 10 ng ml^−1^ dox to resemble 0.5 ng ml^−1^ dox induction, and along a downslope, at 0.5 ng ml^−1^ dox to resemble 0.3 ng ml^−1^ dox induction, respectively. **h**, Predictable, opposite shifts of invasiveness for low-noise mNF-BACH1 (BL) and high-noise mNF-BACH1 (BH) clones when treated with hemin to induce degradation along an upslope and a downslope, compared to control samples. Pink and cyan arrows indicate hemin-dependent BACH1 degradation corresponding to the upslope and downslope in **g**. Unpaired two-tailed *t*-test was used for each comparison, *n* = 3, *P* > 0.05. NS, not significant. Source data

If BACH1 promotes invasion, then its reduction should reduce invasion. To confirm this in MB231 cells, we lowered BACH1 levels in mNF clones by various doses of hemin (Extended Data Fig. 4f). Boyden chamber (transwell) invasion assays^59^ indicated that hemin reduced invasiveness (the ratio of invading versus originally seeded cells) over two-fold in both uninduced mNF-BACH1 and mNF-GFP MB231 clones (Fig. 3a and Extended Data Fig. 4g), as seen with anti-BACH1 short hairpin RNA (shRNA) in 1,833 cells^30^. This indicates similar native BACH1 levels and negligible ectopic BACH1 contribution in all uninduced clones, consistent with the immunofluorescence measurements.

**Extended Data Fig. 5:**
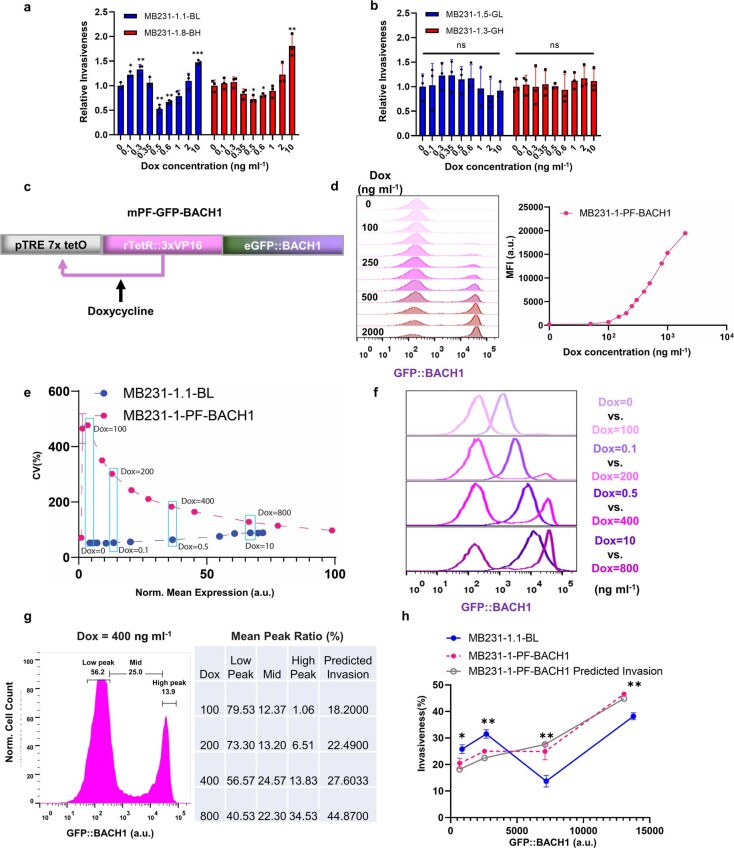
Invasion effects of BACH1 expression noise are landscape-dependent. **(a)** Relative invasiveness of low-noise mNF-BACH1 (BL) and high-noise mNF-BACH1 (BH) clones at each Dox concentration with respect to the uninduced controls. Two-tailed t-test between uninduced control and every other dose, n = 3, *P < 0.05, **P < 0.01, ***P < 0.001. **(b)** Relative invasiveness of low-noise mNF-GFP (GL) and high-noise mNF-GFP (GH) clones at each Dox concentration with respect to the uninduced controls. n = 3, one-way ANOVA, P > 0.05. **(c)** Synthetic mammalian Positive-Feedback (mPF) gene circuit for Dox-induced co-expression of reverse tetracycline-controlled transactivator (rtTA) and GFP::BACH1 fusion protein (mPF-BACH1). TetO: Tetracycline Operator; VP16: Virus Protein 16 transcription activator. **(d) Left panel:** Representative dose-responses of fluorescence intensity histograms from mPF-BACH1 integrated MB231 clone measured at 0, 50, 100, 150, 200, 250, 300, 400, 500, 800, 1000 and 2000 ng ml^−^^1^ Dox levels, respectively. **Right panel:** Dose-responses of mean fluorescence intensity (MFI) for MB231 mPF-BACH1 clone (n = 3, Dox=10 corresponds to Dox=0). **(e)** Plotting the noise (CV) as a function of normalized mean gene expression for both low-noise mNF-BACH1 and mPF-BACH1 clones of MB231. Total four mean-noise decoupling pairs were selected based on the minimum mean differences between mNF and mPF populations. **(f)** Representative histograms of selected mean-noise decoupling pairs in **(e)**. **(g)** Deconstruction of BACH1 expression distribution among mPF-BACH1 population in the context of invasion landscape. Invasiveness predictions for each dose were based on each subpopulation’s mean invasiveness in the context of invasion landscape in Fig. 3e. Mean Peak Ratio was averaged over 3 independent replicates. **(h)** Comparison of invasiveness for mPF versus mNF cells at four selected decoupled noise points along with mPF cell invasion predicted computationally. Two-tailed t-test for each experimental pair, n = 3, *P < 0.05, **P < 0.01. Source data

Because BACH1 downregulation reduced invasiveness, we expected that, conversely, BACH1 overexpression would promote invasiveness. To test this, we tuned BACH1 expression up in MB231 cells. Although at full induction (10 ng ml^−1^ dox) cells were more invasive than without induction, intermediate induction effects did not follow suit (Fig. 3b). Instead, BACH1 overexpression halved invasiveness at 0.5 ng ml^−1^ dox, just like 50 μM hemin did. Generally, the BACH1 invasion landscape (dependence of invasiveness on BACH1 levels) was surprisingly nonmonotone, with a remarkable valley between ~0.3 ng ml^−1^ and 2 ng ml^−1^ dox induction. Even though BACH1 levels kept increasing in this dox range, both low-noise and high-noise mNF-BACH1 clones invaded less than without induction (Fig. 3c and Extended Data Fig. 5a). By contrast, the invasion landscapes of both mNF-GFP clones were flat, without significant changes (Fig. 3d and Extended Data Fig. 5b). Accordingly, mNF-GFP clones were more invasive at 0.5 ng ml^−1^ dox and less invasive at 10 ng ml^−1^ dox than correspondingly induced mNF-BACH1 clones (Fig. 3e). Also, the consistent up/down invasion trends for mNF-BACH1 but not mNF-GFP clones support biological significance. In contrast to MB231 cells, the 293 mNF-GFP and mNF-BACH1 cells failed to invade, regardless of induction.

To test the relationship between BACH1 expression noise and invasiveness, we compared the invasiveness of high-noise versus low-noise clones. Interestingly, the high-noise mNF-BACH1 clone was significantly more invasive at high BACH1 levels (Fig. 3e). Accordingly, the invasiveness differential, Δinvasiveness, between high-noise versus low-noise clones correlated positively with the CV differential, ΔCV but not the mean differential, Δmean (Fig. 3f). A similarly engineered positive feedback (mPF-BACH1) gene circuit with bimodal expression and high noise corroborated these observations (Supplementary Notes 1.2 and Extended Data Fig. 5c–h). Overall, we found that BACH1 noise can enhance the invasiveness of TNBC cell populations.

**Extended Data Fig. 6:**
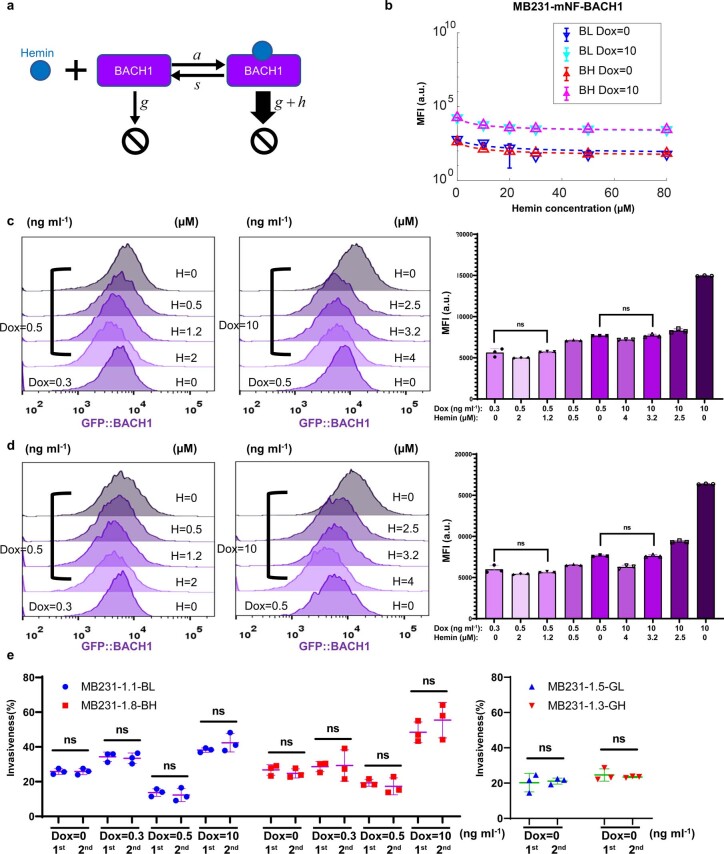
Hemin-promoted BACH1 degradation and reproducibility of invasiveness. **(a)** Schematic illustration of the hemin-promoted BACH1 degradation model. **(b)** Dose-response of steady state BACH1 protein levels versus hemin concentrations. Experimental mean fluorescence intensity (MFI) values are overlaid with predictions from a mathematical model of dose-response of hemin-promoted BACH1 degradation using best-fit parameters to experimental data with autofluorescence background subtraction. BL: low-noise clone; BH: high-noise clone. Modeling details can be found in Supplementary Notes1.1**. (c)(d)** Experimental identification of hemin concentrations needed to achieve BACH1 degradation to specific levels in both low-noise and high-noise clones. Starting from 0.5 ng ml^−^^1^ and 10 ng ml^−^^1^ Dox-induction, we added hemin to reduce BACH1 levels equivalent to 0.3 ng ml^−^^1^ and 0.5 ng ml^−^^1^ Dox induction, respectively. n = 3, unpaired two-tailed t-test, P > 0.05. **(e)** Reproducibility of invasiveness in two independent experiments for both mNF-BACH1 clones at multiple Dox doses **(Left)** and both mNF-GFP clones without induction **(Right)**. Source data

**Extended Data Fig. 7:**
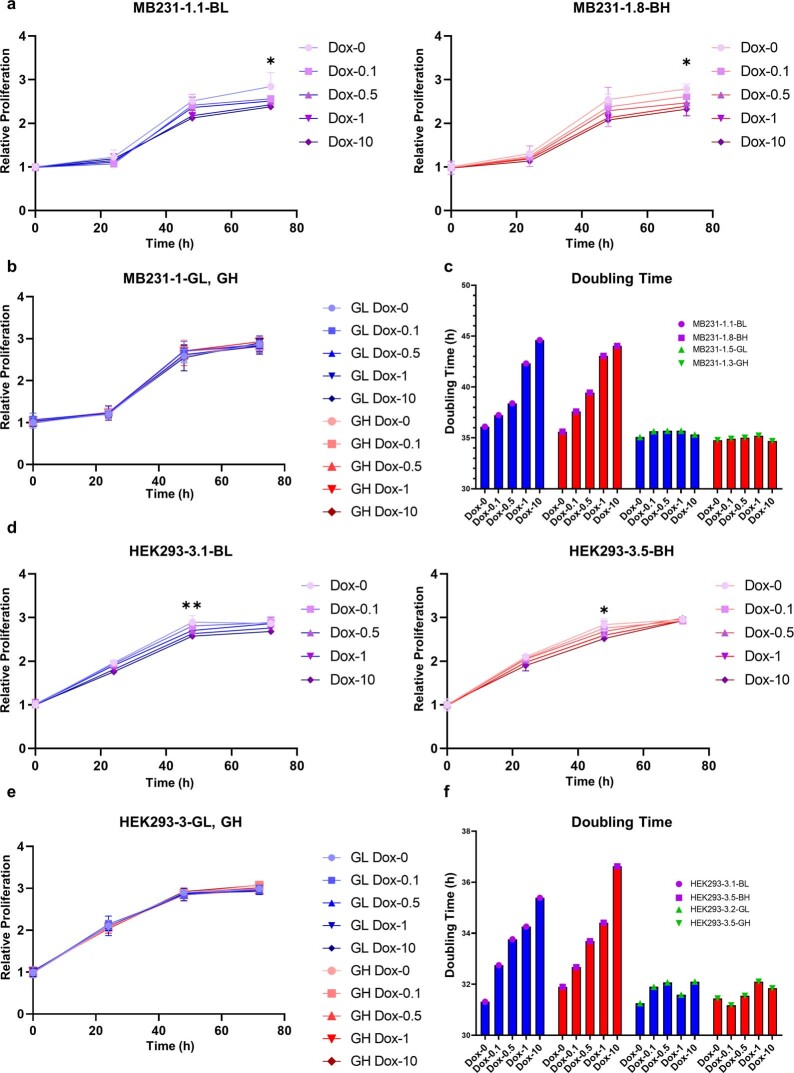
Monotone reduction of cell proliferation due to controlled BACH1 expression increase. Relative proliferation measurements at 0-, 24-, 48- and 72-hour time points for both low-noise and high-noise MB231-mNF-BACH1 clones **(a)**, MB231-mNF-GFP clones **(b)**, HEK293-mNF-BACH1 clones **(d)** and HEK293-mNF-GFP clones **(e)**. **(c) (f)** Doubling times calculated for all selected clones of MB231 **(c)** and HEK293 **(f)**. Doubling times were calculated using time points 0 and 48 h. One-way ANOVA, n = 3, *P < 0.05, **P < 0.01. Source data

BACH1ʼs role as a metastasis activator is generating interest in BACH1 inhibition by hemin for therapy development in TNBC^31^. Such initiatives illustrate the widespread yet simplistic assumptions and naive expectations from protein inhibitors across drug development, pharma and clinical trials. However, the nonmonotone invasion landscape predicts that BACH1 inhibition could unwantedly promote invasiveness on downslopes of the landscape. To examine this possibility, we modeled how hemin treatment lowers BACH1 levels (Extended Data Fig. 6a,b and Supplementary Notes 1.1) and predicted that applying 1.2 µM hemin at a downslope (0.5 ng ml^−1^ dox) or 3.2 µM hemin at an upslope (10 ng ml^−1^ dox) should reduce BACH1 levels to resemble 0.3 ng ml^−1^ dox and 0.5 ng ml^−1^ dox induction, respectively (Fig. 3g). Flow cytometry confirmed the expected BACH1 level reductions (Extended Data Fig. 6c,d), and transwell assays proved that BACH1 reduction lowers invasiveness at high BACH1 expression, yet it promotes invasion at mid-range BACH1 (Fig. 3h). These reproducible observations confirm the antagonistic effects of BACH1-reducing hemin treatment on cellular invasiveness (Extended Data Fig. 6e), which was unlikely attributable to BACH1ʼs influence on cell proliferation^27,31^, because BACH1 upregulation curbed cell proliferation monotonically based on growth curves (Extended Data Fig. 7a,d) and doubling time calculations (Extended Data Fig. 7c,f) in mNF-BACH1 cells, without effects in mNF-GFP cells (Extended Data Fig. 7b,e). Thus, proliferation cannot explain the nonmonotone BACH1 invasiveness landscape.

Overall, these results suggest antagonistic, protein-level-dependent effects of BACH1 overexpression on the invasion of MB231 cells. Moreover, BACH1 expression noise can enhance invasion independently of the mean, in a landscape-dependent manner.

### BACH1 invasion landscape mediates phenotypic selection

Cellular evolution can occur by selection of nongenetic variants^17,60^, according to the Price equation^61–63^. For example, non-genetic cell–cell differences in BACH1 levels could mediate phenotypic adaptation if BACH1ʼs concentration (1) varies from cell to cell; (2) correlates with fitness; and (3) persists between two consecutive observations under selection. Whereas fitness is typically related to cell proliferation, metastasis correlates better with cellular invasiveness, which depends on BACH1 expression according to a metastatic fitness landscape (Fig. 3g and Supplementary Notes 1.3). Cell populations climb that landscape under four main types of selection^64^, depending on the local geography. First, fitness upslopes impose positive directional selection, which enriches for cells with high BACH1 expression, increasing BACH1ʼs mean in invading cells without upregulation. Second, fitness downslopes impose negative directional selection, with effects opposite to positive directional selection. Third and fourth, as we derive by manipulating the Price equation (Supplementary Notes 1.4), fitness peaks and valleys impose stabilizing and disruptive selection, which should reduce or amplify BACH1ʼs variance, respectively. Overall, testing how BACH1 expression mean and variance change in invading cells could validate the nonmonotone BACH1 invasion landscape.

**Fig. 4:**
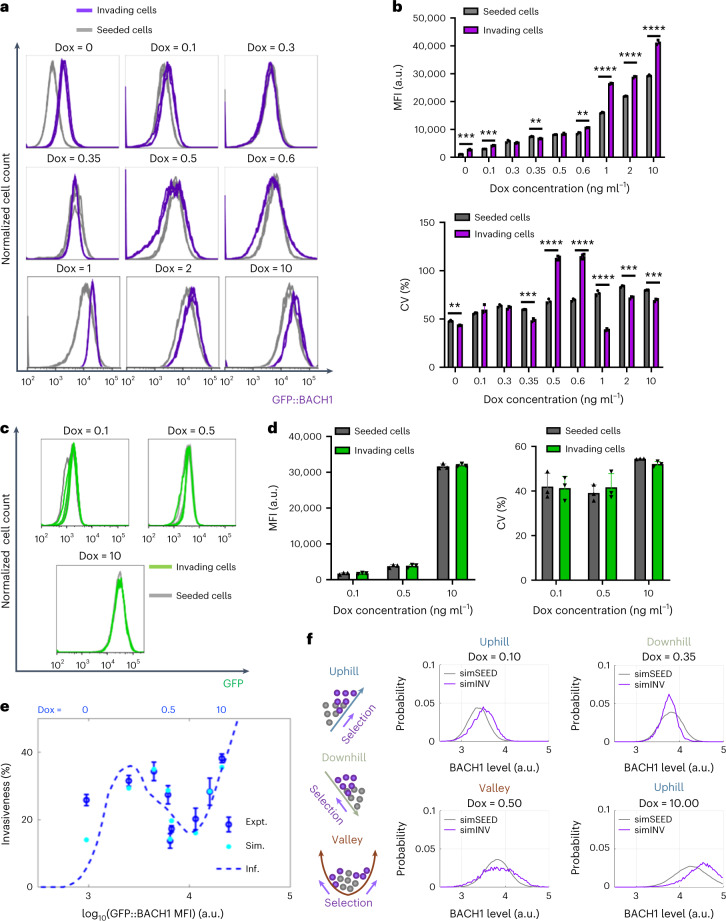
BACH1 expression profile shifts of invading cells indicate directional and divergent selection. **a**, Flow cytometry expression profile changes of invading low-noise BACH1 cells versus seeded cells at multiple points of the invasion landscape (*n* = 3). **b**, MFI (top) and CV (bottom) comparison between invading cells and seeded cells (unpaired two-tailed *t*-test, *n* = 3, **P < 0.01, ****P < 0.0001). **c**, Flow cytometry expression profile comparisons for invading low-noise mNF-GFP cells versus seeded cells at three key dox levels. **d**, MFI (left) and CV (right) of invading versus seeded cells (unpaired two-tailed *t*-test, *n* = 3, *P* > 0.05). **e**, Cellular invasion landscape (dashed blue line) of low-noise mNF-BACH1 (BL) clone versus log_10_(mean BACH1 expression) inferred from flow cytometry histograms of seeded and invading cells. Stochastic simulation results (cyan) for cells invading according to their BACH1 level dependent position on the landscape are compared to the experimental data (blue). **f**, Computationally predicted log_10_(BACH1 expression) profile changes in the invaded cell population at distinct landscape ranges due to directional (fitness slope uphill and downhill) and divergent (fitness valley) selection. Expt., experimental; Inf., inferred; Sim., simulated. Source data

**Extended Data Fig. 8:**
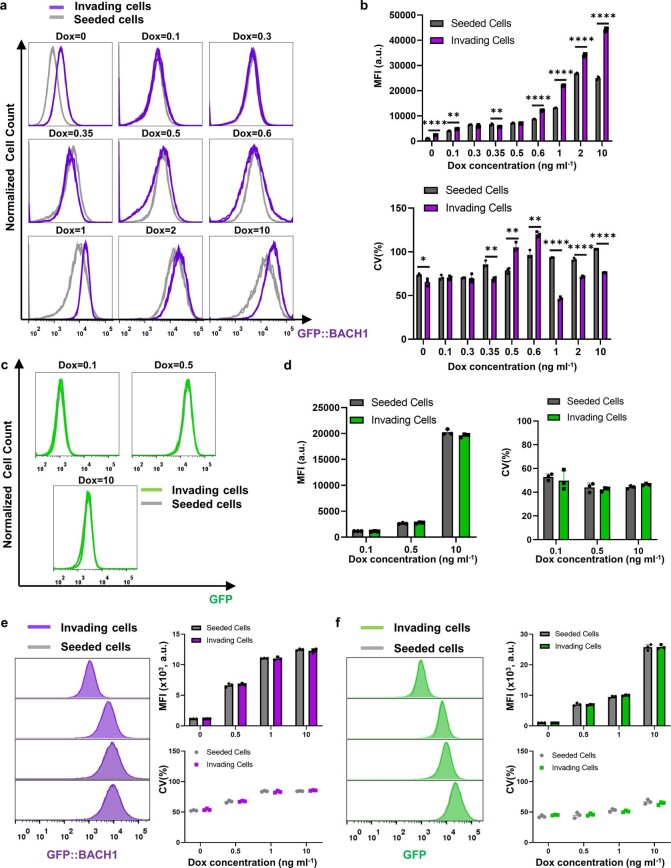
BACH1 expression profile shifts in high-noise clones and loss of BACH1 expression memory. **(a)** Flow cytometry assessment of BACH1 expression profile changes in invading versus seeded cells at multiple points of the invasion landscape. These measurements are from the high-noise mNF-BACH1 MB231 clone, with three independent technical replicates. **(b)** Mean fluorescence intensity **(top panel)** and coefficient of variation **(bottom panel)** of invading versus seeded cells (unpaired two-tailed Student’s t-test, n = 3, *P < 0.05, **P < 0.01, ****P < 0.0001). **(c)** BACH1 expression profile-reshaping in invading cells versus seeded cells at three key Dox levels. These measurements are from the high-noise mNF-GFP MB231 clone with three independent technical replicates. **(d)** Mean fluorescence intensity **(left panel)** and coefficient of variation **(right panel)** for invading versus seeded cells (unpaired two-tailed t-test, n = 3, P > 0.05). **(e)(f)** Expression mean and noise measurements of the invading subpopulation of cells from low-noise mNF-BACH1 **(e)** and mNF-GFP **(f)** clones from the 10 ng ml^−^^1^ Dox invasion assay, which were allowed to recover in normal culture condition and were then reinduced. The data was compared to the seeded cell populations. Unpaired two-tailed t-test for each comparison, P > 0.05. Source data

To test nonmonotone phenotypic selection along the landscape, we performed invasion assays at increasing dox concentrations and examined BACH1 expression profiles of invading MB231 cells harvested below the membrane insert of Boyden chambers. Interestingly, expression distributions and means of the invading versus seeded low-noise (Fig. 4a,b) and high-noise (Extended Data Fig. 8a,b) mNF-BACH1 cells shifted differently at various dox doses: upward at 0, 0.1,1, 2 and 10 ng ml^−1^ dox but downward at 0.3 ng ml^−1^ and 0.35 ng ml^−1^ dox. Strikingly, at 0.5 ng ml^−1^ and 0.6 ng ml^−1^ dox, BACH1 distribution in invading cells broadened, and their CV increased compared to the seeded cells, as expected from disruptive selection at a fitness valley. Meanwhile, eGFP distributions of invading and seeded cells were indistinguishable over multiple doses in both mNF-GFP clones, corroborating the flat invasion landscape (Fig. 4c,d and Extended Data Fig. 8c,d). The expression shifts are not due to genetic mutations, because passaging and reinducing invading cells harvested from 10 ng ml^−1^ dox caused their BACH1 expression to return to its original distribution, supporting selection of phenotypic variant cells (Extended Data Fig. 8e,f).

**Extended Data Fig. 9:**
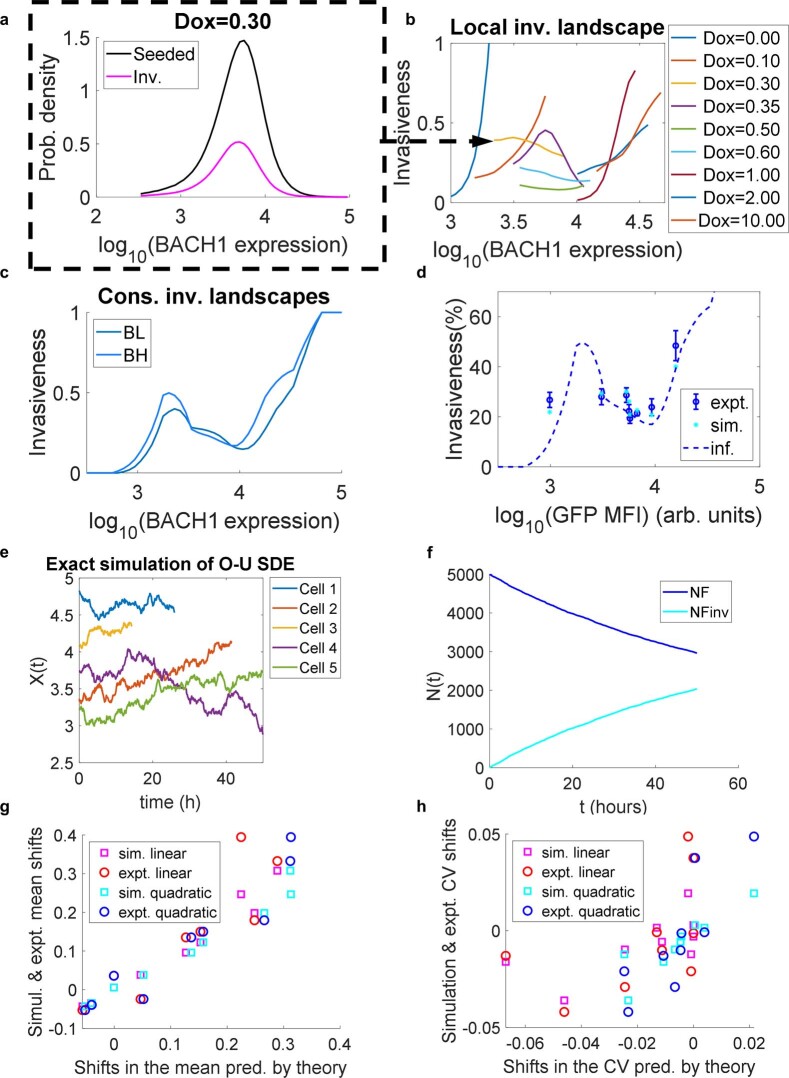
Theory and simulation of phenotypic selection by the nonmonotone invasion landscape. **(a)** Histograms of seeded and invaded cells. Ratios of bin heights were used to estimate the local cellular fitness landscape for each Dox concentration. **(b)** Local cellular fitness landscapes estimated for each Dox concentration. **(c)** Global consensus cellular fitness landscapes for low-noise and high-noise cells, estimated as weighted averages of local fitness landscapes. **(d)** Simulated values of high-noise mNF invasiveness (cyan) compared to the experimental invasion landscape (blue - data, dashed blue line – consensus cellular invasion landscape). **(e)** Representative simulated Ornstein-Uhlenbeck (O-U) time courses of BACH1 levels in 5 single seeded cells. Some time courses terminate when seeded cells with higher BACH1 levels invade. **(f)** Simulated time courses of cell count above and below the membrane over 50 hours in invasion assays. **(g)** The shift of means for invading versus control seeded cells according to simulation and experiment, versus theory developed for linear and quadratic approximations of the fitness landscape, for low-noise mNF cells. **(h)** The shift of CVs for invading versus control seeded cells according to simulation, and experiment versus theory developed for linear and quadratic approximations of the fitness landscape, for low-noise mNF cells. Expt., experimental; Inf., inferred; Sim., simulated.

Previously, we showed that fitness, like other phenotypes, can vary across genetically identical cells^40,52^. Likewise, the invasiveness of individual cells could differ markedly from the cell population’s average invasiveness. To gain insight into single-cell invasiveness and confirm BACH1 expression shifts by phenotypic selection, we inferred single-cell invasion landscapes (Fig. 4e and Extended Data Fig. 9a–d) from experimental data (Supplementary Notes 1.5). Stochastic simulations^65^ of cells that invade on single-cell invasion landscapes (Extended Data Fig. 9e,f), according to their fluctuating log_10_(BACH1) levels, confirmed that fitness upslopes/downslopes and valleys can cause the experimentally observed shifts in BACH1 expression mean and variance (Fig. 4f), according to theoretical predictions based on the Price equation (Extended Data Fig. 9g,h).

Overall, mathematical and computational models of various selective invasion effects on cellular BACH1 expression explained histogram shifts observed experimentally, validating the nonmonotone invasion landscape of BACH1 expression in MB231 cells.

### Native BACH1 does not cause nonmonotonicity

**Fig. 5:**
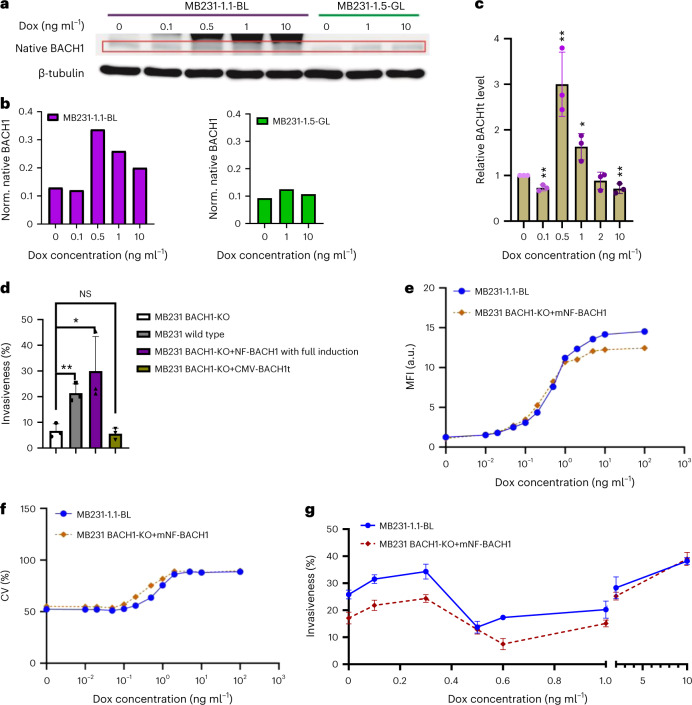
BACH1ʼs nonmonotone invasion landscape is independent of native BACH1. **a**, Western blot examination of native BACH1 protein level dose response in low-noise mNF-BACH1 and mNF-GFP MB231 samples. Native BACH1 bands are highlighted in the red rectangle. **b**, Quantitation of native BACH1 protein level dose response in low-noise mNF-BACH1 and mNF-GFP MB231 samples using grayscale normalization to internal reference β-tubulin in **a**. **c**, mRNA level dose responses of truncated BACH1 isoform, BACH1t, in low-noise mNF-BACH1 MB231 clones. BACH1t is transcribed only from the native copy of BACH1 gene. One-way ANOVA with Tukey’s multiple comparisons tests between each dose and uninduced controls, *n* = 3, *P < 0.05, **P < 0.01. **d**, Invasiveness significantly decreased due to BACH1 knockout (KO) compared to the wild-type parental population. Invasiveness can be rescued by transient overexpression from the induced mNF-BACH1 circuit but not by BACH1t overexpression. Two-tailed *t*-test between BACH1-KO sample and every other condition, *n* = 3, *P < 0.05, **P < 0.01. **e**,**f**, Dose responses of MFI (**e**) and CV (**f**) for a selected MB231 BK monoclone with AAVS1 site-specifically integrated mNF-BACH1 circuit relative to the low-noise mNF-BACH1 (BL) clones (*n* = 3). **g**, Invasiveness of a selected MB231 BK + mNF-BACH1 circuit monoclone relative to low-noise mNF-BACH1 (BL) clones over increasing dox concentrations (*n* = 3). NS, not significant. Source data

Ectopic BACH1 transcription is under synthetic gene circuit control, practically unaffected by native transcriptional regulation. On the other hand, BACH1 overexpression perturbs BACH1ʼs native regulatory network (Fig. 1a), which contains multiple feedback loops^29^, and other interactions. Thus, seeking clues to the nonmonotonicity, we investigated if ectopic eGFP::BACH1 had a nonmonotone effect on native BACH1 expression, the two proteins being distinguishable by western blotting based on their size (Fig. 5a). With a peak around 0.5 ng ml^−1^ dox induction, native BACH1 levels changed in a nonmonotone manner opposite to the invasion landscape upon ectopic BACH1 tuning in MB231 mNF-BACH1 cells, without change in mNF-GFP cells (Fig. 5b). We confirmed these trends at the mRNA level, focusing on the C-terminus-truncated mRNA isoform BACH1t^54^ co-expressed with native BACH1 (Fig. 5c).

To further investigate how native BACH1 affects nonmonotonicity, we stably deleted the native BACH1 gene by CRISPR–Cas9, creating the MB231 BACH1-knockout cell line (Supplementary Fig. 4a,b). BACH1ʼs deletion upregulated its transcriptional target HMOX1 and lowered MB231 BACH1-knockout invasiveness compared to parental MB231 cells (Supplementary Fig. 4c). These phenotypes were rescued by transient reintroduction of ectopic BACH1, but not BACH1t, which lacks the DNA-binding domain^54^ (Fig. 5d). Next, we integrated and tested the mNF-BACH1 gene circuit with a silent BACH1 mutation to avoid cutting by Cas9 in MB231 BACH1-knockout cells (Supplementary Fig. 4a and Fig. 5e,f). Invasion assays revealed a nonmonotone invasion landscape resembling that of the low-noise mNF-BACH1 clone (Fig. 5h). Thus, nonmonotonicity is independent of native BACH1ʼs presence, but BACH1 deletion reshapes somewhat the MB231 BACH1 invasion landscape.

### Transcriptional regulation of BACH1 targets consistent with invasion landscape

**Fig. 6:**
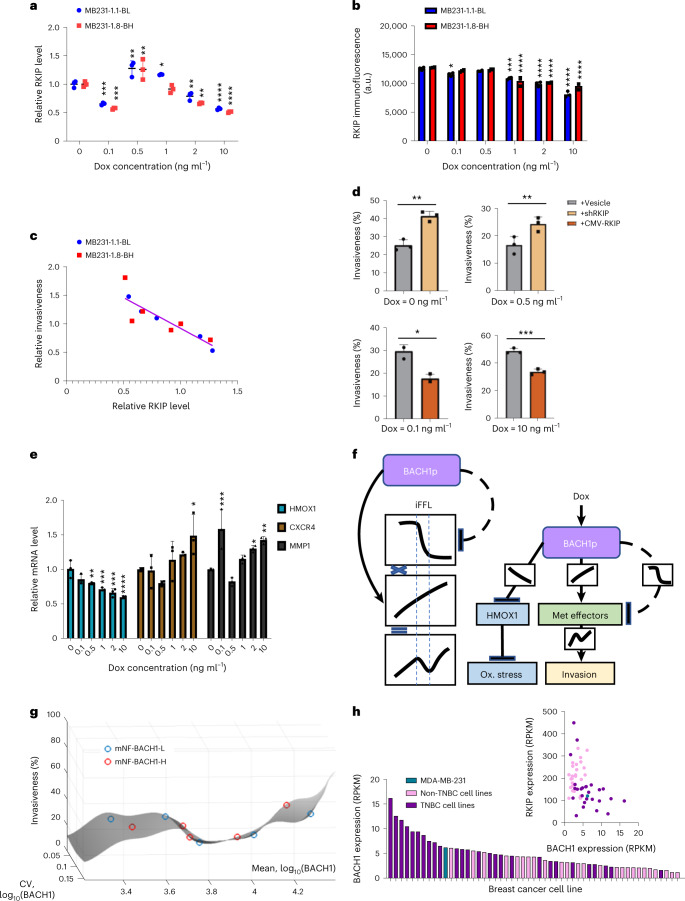
Nonmonotone transcriptional regulations of metastasis-related targets from BACH1 consistent with nonmonotone invasion landscape. **a**, RKIP mRNA level changes for increasing dox concentrations in both low-noise mNF-BACH1 (BL) and high-noise mNF-BACH1 (BH) MB231 clones with respect to the corresponding uninduced sample. *n* = 3, one-way ANOVA with Tukey’s multiple comparisons tests between each dose and uninduced controls, *P < 0.05, **P < 0.01, ***P < 0.001, ****P < 0.0001. **b**, Immunofluorescence quantitation of RKIP protein dose responses in both low-noise mNF-BACH1 (BL) and high-noise mNF-BACH1 (BH) MB231 clones with respect to the corresponding uninduced sample. *n* = 3, one-way ANOVA with Tukey’s multiple comparisons tests between each dose and uninduced controls, *P < 0.05, ***P < 0.001, ****P < 0.0001. **c**, Dose-responsive RKIP expression correlates negatively with nonmonotonic BACH1 invasion landscape, *r* = −0.8766, *P* = 0.0002. Samples were averaged with *n* = 3 technical replicates and normalized to the mean of corresponding uninduced sample, Pearson correlation. **d**, RKIP-dependent invasiveness changes at 0, 0.1, 0.5, 10 ng ml^−1^ dox concentrations. RKIP was overexpressed at 0.1 ng ml^−1^ and 10 ng ml^−1^ dox levels where it was significantly more suppressed, and RKIP was knocked down via shRNAmir at 0 and 0.5 ng ml^−1^ dox levels where it was much less suppressed. Two-tailed *t*-test between each experimental pair, *n* = 3, *P < 0.05, **P < 0.01, ***P < 0.001. **e**, HMOX1, MMP1 and CXCR4 mRNA level changes at increasing dox concentrations in low-noise mNF-BACH1 (BL) MB231 clones with respect to the corresponding uninduced sample. *n* = 3, one-way ANOVA with Dunnett’s multiple comparisons test between each dose and uninduced controls, *P < 0.05, **P < 0.01, ***P < 0.001, ****P < 0.0001. **f**, Regulatory network model that illustrates how iFFLs originating from BACH1 and converging on metastasis effectors such as CXCR4 and MMP1 can potentially underlie the nonmonotone invasion landscape and transcriptional regulation. Combining sharp and gradual response functions of opposite effects (activating and inhibitory) results in nonmonotone response functions. Solid lines indicate known regulatory pathways, whereas dashed lines indicate indirect, somewhat hypothetical pathways. Sigmoidal or gradual functions on the connections represent the characteristics of response curves for pairs of regulators. **g**, Invasion landscape interpolated based on invasiveness data points versus both mean and noise (CV) of log_10_ BACH1 expression, using biharmonic spline interpolation. **h**, Top: comparison between RNA levels of BACH1 and RKIP in TNBC (*n* = 28) cell lines and non-TNBC (*n* = 25) breast cancer cell lines. Bottom: BACH1 mRNA expression in major breast cancer cell lines classified into TNBC and non-TNBC categories. Met, metastasis, Ox., oxidative; RPKM, reads per kilobase of transcript per million mapped reads. Source data

**Extended Data Fig. 10:**
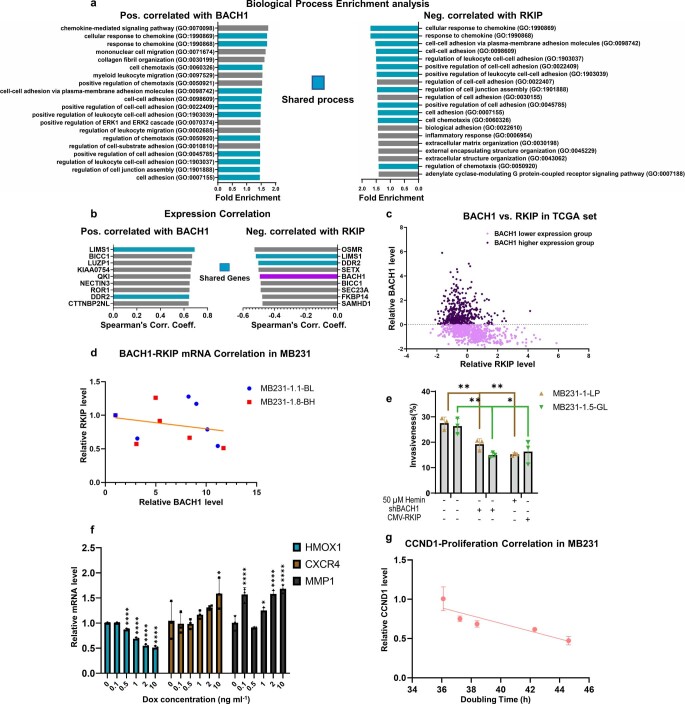
Phenotypic effects of BACH1 correlate significantly with downstream target gene expression. **(a)** Top 20 cell-motility-related biological processes from gene set enrichment (GSE) analysis performed for genes positively correlated with BACH1 **(left)** and for genes negatively correlated with RKIP **(right)** in the TCGA BRCA set (breast cancer; provisional, n = 1100), ranked by fold enrichment coefficient. Cyan-labeled processes are shared between the two GSE sets. **(b)** Top 10 genes with expression positively correlated with BACH1 expression **(left)** and negatively correlated with RKIP expression **(right)** in the TCGA BRCA set. Cyan-labeled genes are shared between two lists. **(c)** Relative BACH1 mRNA expression z-score with respect to the average expression of diploid samples in the TCGA BRCA Provisional dataset (n = 1100). Pearson Correlation value between total BACH1 and RKIP was r = −0.3690, P < 0.0001; while for BACH1 lower and higher expression groups were r = −0.3861, P < 0.0001 and r = 0.0062, P = 0.8930 respectively. **(d)** Correlation between RKIP mRNA and corresponding BACH1 mRNA level at each Dox concentration in both low-noise mNF-BACH1 (BL) and high-noise MB231 mNF-BACH1 (BH) clones, r = −0.2486, P = 0.4359. Samples were averaged with n = 3 technical replicates and normalized to the mean of corresponding uninduced sample, Pearson Correlation. **(e)** The microRNA-mediated BACH1 knockdown, hemin-mediated BACH1 degradation and RKIP overexpression repress invasiveness comparably. Two-tailed t-test between control samples and every other condition, n = 3, *P < 0.05, **P < 0.01. **(f)** mRNA level changes of HMOX1, MMP1 and CXCR4 for increasing Dox concentrations in high-noise mNF-BACH1 (BH) MB231 clones with respect to the corresponding uninduced sample. n = 3; one-way ANOVA with Dunnett’s multiple comparisons test between each dose and uninduced controls, *P < 0.05, **P < 0.01, ***P < 0.001, ****P < 0.0001. **(g)** Dose-response of CCND1 expression correlates significantly with monotone reduction of proliferation as BACH1 levels increase. r = −0.8988, P = 0.0381, n = 3, Pearson Correlation. Source data

Higher levels of transcriptional repressors should reduce the levels of their target proteins. BACH1 is a direct transcriptional repressor of metastasis suppressor RKIP^29^ (Fig. 1a), so increasing BACH1 should reduce RKIP expression^66^. Indeed, The Cancer Genome Atlas (TCGA) gene sets and biological functions antagonistically correlated with RKIP and BACH1 overlapped largely, being enriched in functions such as cell motility (Extended Data Fig. 10a,b). However, if RKIP contributes to BACH1ʼs invasion effects, then RKIP’s response to BACH1 upregulation might be nonmonotone. To test this, we measured RKIP mRNA levels at increasing dox doses using qRT–PCR in both low-noise and high-noise mNF clones from each cell line. Remarkably, RKIP levels responded to BACH1 upregulation nonmonotonically (Fig. 6a). Increasing BACH1 significantly suppressed RKIP at lower (0.1 ng ml^−1^) and higher (5 ng ml^−1^ and 10 ng ml^−1^) dox doses, as expected. However, at intermediate dox doses (0.5 ng ml^−1^ and 1 ng ml^−1^), BACH1 upregulated RKIP, all the way to overexpression. Immunofluorescence corroborated this observation at the protein level (Fig. 6b). Consistently, gene expression data from clinical breast tumor samples revealed that, although RKIP correlates inversely with BACH1 overall, the two transcripts cease to correlate when BACH1 surpasses a threshold, as in our cell line experiments (Extended Data Fig. 10c,d).

To confirm that RKIP contributes to BACH1ʼs invasion effects, we uncovered a significant negative correlation by plotting relative invasiveness versus relative RKIP expression at increasing dox concentrations (Fig. 6c). RKIP overexpression or BACH1 reduction by hemin or shRNAmir^67^, an shRNA embedded into a microRNA backbone, lowered the invasiveness of MB231 LP parental or mNF-GFP cells (Extended Data Fig. 10e). Transiently expressing RKIP or RKIP-targeting shRNAmir decreased and increased invasiveness, respectively (Fig. 6d). Overall, the strong correlation between the nonmonotone invasion landscape and nonmonotone RKIP regulation by BACH1 suggests that BACH1ʼs invasion effects might involve RKIP.

Curious if BACH1ʼs nonmonotone effects extend to other transcriptional targets, we tested how CXCR4 and MMP1 (ref. ^30^) respond to ectopic BACH1 tuning. We found nonmonotone expression changes that matched the invasion landscape. In contrast, HMOX1 expression decreased monotonically (Fig. 6e and Extended Data Fig. 10f), similarly to CCND1 expression and cell proliferation upon BACH1 tuning (Extended Data Fig. 10g). Previous studies as well as our calculations (Supplementary Notes 1.6 and 1.7) suggest that BACH1-driven incoherent feedforward loops (iFFLs) can generate the nonmonotone responses^68^ (Fig. 6f). Overall, the expression of multiple metastasis-related BACH1 targets, including RKIP, CXCR4 and MMP1, corroborates the nonmonotone BACH1 invasion landscape, suggesting that they might mediate BACH1ʼs effects in MB231 cells. On the other hand, some monotone responses to BACH1 tuning indicate at least two distinct modes of BACH1-driven transcriptional regulation: by iFFLs and by simple regulatory links (Fig. 6f).

## Discussion

Protein levels are closer to cellular phenotype than most other molecular characteristics of single cells. Protein level-to-phenotype (P2P) mapping is a crucial step in solving the outstanding genotype-to-phenotype problem of modern biology and medicine^69^ and could advance prognosis and treatment of diseases, including cancer. Cancer cells develop abnormality both as a cause and consequence of deviant protein levels^17,29,52^. Artificial, precise protein level perturbations could provide important, causal information for the P2P map^19^, requiring technologies that are available^70^ but insufficiently used across human cell lines. Consequently, quantitative, noise-aware P2P mapping is lacking for many phenotypes, including metastatic behaviors. To close this technology–knowledge gap, we demonstrate two-step SSR/SHS genome modification for SHS-specific, repeatable, single-copy integration of protein level tuning synthetic gene circuits, which we apply to map TNBC cell invasiveness versus the average and variability of BACH1 levels.

In this study, we combined SSR/SHS with synthetic gene circuit engineering to precisely control both the average and variance of BACH1 levels in a TNBC cell line. Contrary to the assumption that higher mean levels of a metastatic activator should promote more invasion^30^, we reveal a nonmonotone invasion landscape (Fig. 6g), showing that BACH1 can suppress invasion within a certain expression range. Furthermore, BACH1 nonmonotonically regulates the expression of multiple genes, such as RKIP^29,30^, CXCR4, MMP1 and even BACH1 itself, acting alternatively as an activator and a repressor over various expression ranges. We propose that such nonmonotone effects arise from combining opposite (activating and repressing) sharp and gradual responses to BACH1 (Fig. 6f) via iFFLs^68^. The microRNA Let-7 might be part of these iFFLs, because its targets can respond sharply to upstream expression changes^29^. Moreover, both BACH1 knockdown and overexpression repress SNAI2 expression^71^, and BACH1 can both activate and repress its targets^28^, which further imply iFFLs. However, HMOX1 and other regulatory targets have monotone responses to BACH1, indicating at least two distinct modes of gene regulation (Fig. 6f). Breast cancer cell line RNA sequencing (RNA-seq) data^21,72^ indicate higher BACH1 and lower RKIP expression in TNBC cell lines, suggesting TNBC-specific alterations of this regulatory network (Fig. 6h). Anticorrelation between BACH1 and RKIP in the TCGA breast cancer dataset up to a BACH1 level echoes our findings in MB231 cells. Identifying the unknown BACH1–RKIP interactors mediating this nonmonotonicity warrants further studies.

Studies in bacteria^33,73^, yeast^34,35^ and human cells^41^ indicate that nonmonotone fitness landscapes are common, including the effects of oncogenes and other drug targets, raising concerns about adverse effects of inhibiting them simplistically. Indeed, lowering BACH1 levels^31,32^ around landscape downslopes can adversely promote invasion and possibly metastasis. Counterintuitively, near such downslopes BACH1 upregulation may be desirable to reduce both invasiveness and cell proliferation, to improve clinical benefits. If cell lines represent inter-patient or intra-tumor diversity, then intermediate BACH1 expression in MB231 cells (Fig. 6h) suggests that BACH1 suppression might boost invasiveness in TNBC. Likewise, gene therapies or immunotherapies should consider the adversity of improper therapeutic gene expression by phenotypic landscape mapping.

We recently suggested^19^ that gene expression noise can aid or hinder drug resistance or metastatic steps^6,10,17,19,74^. Likewise, BACH1 expression noise can facilitate TNBC cell invasion at high BACH1 levels, whereas, at low BACH1 levels, noise may hinder invasion, as BACH1 noise interplays with the geography of the invasion landscape (Fig. 6g). So, suppressing or enhancing noise accordingly by chemicals^75,76^ or gene circuits^52^ could diminish metastatic tendencies. However, to avoid unwanted side effects, such noise control should also consider mapping cellular fitness versus the CV.

It will be interesting to similarly define uni-dimensional and multi-dimensional landscapes by recruiting new SHSs^77^ for other genes, phenotypes and human cell types in vitro or in vivo, exploring their predictive value for long-term evolution^52,74,78^. Expression shifts by phenotypic selection^64^ could confirm the landscapes, including fitness valleys that allow evolutionary branching^79^ or possibly phenotypic bifurcations^80^ of cancer cells in the proliferation–invasion space. These research strategies should be scalable to many genes and cell types, enabling quantitative phenotypic landscape mapping to unravel disease biology or to improve the accuracy and efficiency of drug development.

## Methods

### Cell culture

MDA-MB-231 and HEK293 cells were from the American Type Culture Collection. All engineered versions of HEK293 cells (referred to as 293) were cultured in DMEM media with 10% FBS and 1% penicillin–streptomycin. All engineered versions of MDA-MB-231 cells (referred to as MB231) were grown in RPMI 1640 media with 5% FBS and 1% penicillin–streptomycin. Both cell lines were maintained in Panasonic MCO-170AICUVL-PA cellIQ Series CO_2_ incubators at 37 °C and 5% CO_2_ and passaged regularly every 2–4 d. The cells were used in experiments within 15 passages after their arrival in the laboratory.

### Plasmid construction

For the generation of the LPutopia-bearing cell lines (LP clones), the **LPutopia-7** genome-targeting vector was constructed based on the earlier version LPutopia-3 assembled from cloning vectors **DC-RFP-SH01** (human AAVS1 safe harbor gene knock-in kits and clones, GeneCopoeia) and Addgene plasmid 92078 **PB_CMV_GFP_FRT**. The **LPutopia-7** vector contains HA regions to the human AAVS1 locus, which can site-specifically recombine after double-strand break generation by CRISPR–Cas9. In between the HA regions, **LPutopia-7** contains a reporter-selectable cassette consisting of a CMV promoter-driven eGFP reporter and a thymidine kinase (TK) promoter-driven neomycin resistance gene, all flanked by heterotypic FRT sequences (FRT/FRT3). Outside the HA regions, the vector bears RFP as a secondary negative selection marker, which wards against random integration events.

For constructing the RMCE vectors, we first built **pUt-NF-BACH1** that contains a TetR-based mNF gene circuit controlling the expression of the GFP::BACH1 fusion based on the BACH1 open reading frame (ORF) from GeneCopoeia (NM_206866, HPRM54453). The mNF cassette was obtained from Addgene plasmid 128253, **pDN-D2irTN2AG5kwh**. Besides the mNF unit, the vector also included a separate CMV promoter-driven copy of the blasticitin resistance gene BsrS2 from **pUNO-CodA::Upp** (InvivoGen) as a positive selection marker for successful RMCE events. Heterotypic FRT sequences (FRT/FRT3) oriented as in the LPutopia-7 vector flanked both the mLin gene circuit and the BsrS2 gene. Outside the FRT/FRT3 sequences, a PGK promoter-driven herpes simplex virus (HSV) TK gene obtained from **pHR(KRAS.B)-GFP** (GeneCopoeia) was also integrated into the vector as a negative selection marker for non-specific integration events. The RMCE vector **pUt-NF-BACH1-P2A-GFP** was subsequently constructed by adding the P2A sequences between the BACH1 and GFP ORFs. Finally, the RMCE vector **pUt-NF-GFP** was obtained by deleting the BACH1 ORF from the mNF-BACH1 plasmid, keeping everything else the same as described above.

The C-terminus-truncated BACH1 isoform, **BACH1t** (NR_027655.3), and **RKIP/PEBP1** (NM_002567.4) ORF were ordered from Integrated DNA Technologies (IDT) and cloned under the CMV promoter. The shRNAs embedded into an optimized miR-30 backbone^67^, also called ‘shRNAmir’, targeting **BACH1** and **RKIP** were designed using the online tool splashRNA^81^ (http://splashrna.mskcc.org/). Also, the **pUt-PF-BACH1** gene circuit was similarly cloned by combining the mPF circuit components from **pKF-P14MM2AG5h** (Addgene plasmid 128254) and GFP::BACH1 fusion sequence from **pUt-NF-BACH1**.

The enhanced spCas9-expressing vector **eSpCas9(1.1)** (Addgene plasmid 71814) was a gift from Feng Zhang. Using the eSpCas9(1.1) plasmid, we constructed the AAVS1-targeting eSpCas9 vector by adding AAVS1 single guide RNA (sgRNA) (Supplementary Table 1) into the plasmid’s expression scaffold after BbsI restriction digestion. The codon-optimized FLP recombinase-expressing vector **pCAG-Flpo** (Addgene plasmid 60662) was a gift from Massimo Scanziani. We used NEBuilder HiFi DNA Assembly in molecular cloning to fuse DNA pieces together.

### PCR genotyping and copy number determination

PCR genotyping was performed using 50–100 ng of genomic DNA with OneTaq DNA Polymerase (OneTaq Quick-Load 2× Master Mix with Standard Buffer, New England Biolabs, M0486S) in 25-μl reactions. Primer sequences for constructs in Extended Data Fig. 1a,f are listed in Supplementary Tables 2 and 3.

The relative transgene integration copy number for each monoclonal sample was determined for *eGFP* with the TaqMan Copy Number Assay, using the TaqMan Fast Advanced Master Mix (Thermo Fisher Scientific, 4444557). For each qPCR reaction, 100 ng of genomic DNA was run using the QuantStudio 3 Real-Time PCR System (Eppendorf, A28137) in standard curve mode. We used equal amounts of purified genomic DNA of each sample and human RNase P (RPPH1) as internal reference (Supplementary Table 4). eGFP copy number in every clone was calculated based on determined copy number of RPPH1 reference in both 293 genomes and MB231 genome using the ΔΔCt method.

### Transfection and cell sorting

We applied lipofection to transfect all HEK293-derived cells. Before transfection, cells were plated in six-well plates and grown to ~80% confluence. Then, LPutopia-7/espCas9 or pCAG-Flpo/NF circuit donor vector combinations were co-transfected at a 1-to-1 ratio with a final mass of 2.5 µg per well. The vectors were first incubated with 3.75 µl of Lipofectamine 3000 (Invitrogen, L3000-015) in OPTI-MEM media (Gibco, 31985062) for 15–30 min. The resulting DNA–lipid complex was then pipetted onto the cells and then incubated for at least 24 h before refreshing media. Appropriate drugs for selection were added 72 h after transfection. Drug selection lasted for at least 14 d before fluorescence-activated cell sorting (FACS). We used 1,000 µg ml^−1^ of G418 for HEK293 LP cell selection and 10 µg ml^−1^ blasticidin with 10 µg ml^−1^ ganciclovir for HEK293 mNF cell selection.

For all MDA-MB-231-derived cells, nucleofection was performed based on the manufacturer’s instructions and recommendations. In brief, newly thawed MB231 cells were plated in a T-25 flask and subcultured 3–5 d before nucleofection. Next, cells were harvested by adding trypsin and counted using a Cellometer Auto T4 (Nexcelom Bioscience). Around 2 × 10^6^ cells were collected and centrifuged at 200*g* for 10 min at room temperature. Then, the supernatant was removed, and cells were resuspended in 100 µl of room temperature Nucleofector Kit V (Lonza, VCA-1003) solution. The LPutopia-7/espCas9 or pCAG-Flpo/NF circuit donor vector combinations were co-transfected at a 1-to-1 ratio with a final mass of 2 µg per sample. The cell/DNA suspension was transferred into the certified Nucleofector cuvette, and the X-013 program of Nucleofector 2b Device (Lonza, AAB-1001) was applied. Finally, transfected cells were buffered with fresh media and gently transferred into a freshly prepared six-well plate. Drug selection started 24–48 h after nucleofection and lasted for at least 14 d before FACS. We used 700 µg ml^−1^ of G418 for MB231 LP cell selection and 5 µg ml^−1^ blasticidin with 10 µg ml^−1^ ganciclovir for MB231 NF cell selection.

### Stable native BACH1 knockout and reintroduction of ectopic BACH1

MDA-MB-231 (MB231) knockout cell lines were established by lentivirus-based genomic integration of a CRISPR–Cas9 system. Lentivirus stocks were generated by using lentiCrisprv2 (Addgene) with sgRNA targeting BACH1 exon 2 (sequence: CTCAAGAATCGTAGGCCAGG)^71^ (sgRNA sequences are listed in Supplementary Table 1). Infected MDA-MB-231 cells were polyclonally selected and cultured in medium supplemented with 4 μg ml^−1^ puromycin for 1 week.

After verification of the native BACH1 knockout, MB231 BACH1-knockout cells were further co-transfected with **LPutopia-7** donor vector and AAVS1 sgRNA to generate the stable MB231 BK-LP parental cells as described above. Meanwhile, the ectopic BACH1 sequence was single-site mutated at nucleotide 177, changing it from C to T, to disable the PAM site recognition by Cas9, to avoid unwanted cutting of the ectopic BACH1 copy. Later, the silent-mutated **pUt-NF-BACH1** circuit was exchanged into the MB231 BACH1-knockout-LPutopia cells through the same RMCE process and selection method as for the other cell lines. We enriched for recombinants and then performed monoclonal screening to minimize the unpredicted side effects of genome instability induced from constitutively expressed Cas9.

### Fluorescence microscopy

Microscopy was performed 48 h after induction. Cells were imaged in 24-well plates before flow cytometry using a Nikon Eclipse Ti-E inverted microscope with a DS-Qi2 camera (14-bit) for phase contrast and fluorescence images. A ×10 Ph1 objective (type: CFI Plan Fluor) was used in phase contrast and fluorescence mode imaging. The microscope was equipped with Chroma cubes including DAPI 1160B NTE (cat. no. 49000, excitation 395*/*25, emission 460*/*50) for DAPI, ET GFP (cat. no. 49002, excitation 470*/*40, emission 525*/*50) for FITC*/*GFP and ET mCH*/*TR (cat. no. 49008, excitation 560*/*40, emission 630*/*75) for TX Red. Each image was captured under the same exposure time and exported under the same scale of Look Up Table.

### Flow cytometry

For each sample, newly thawed cells were cultured for one passage before the experiment. Next, around 50,000–80,000 cells harvested from 80% confluent T-25 flasks were plated into 24-well plates, with three technical replicates for each inducer concentration. Dox was added into each well to obtain concentrations ranging from 0.01 ng ml^−1^ to 100 ng ml^−1^. Cells were incubated for 2 d (48 h) and then collected into a 96-well plate at a final volume of 250 µl per well and then read on a BD LSRFortessa flow cytometer with High Throughput Sampler at the Stony Brook Genomics Core Facility. GFP fluorescence signal data from at least 10,000 events for each dox concentration were collected within a predefined FSA/SSA gate in the FITC channel with identical PMT voltage settings across all induction levels of every sample (Supplementary Fig. 5).

To determine the reproducibility of gene expression histograms, cells were freshly thawed and incubated for the first week before flow cytometry testing. Then, we performed the dose–response measurements for three technical replicates as described above repeatedly over 4 weeks for each sample at several selected dox concentrations and compared the fluorescence intensity means between measurements from three timepoints.

### RNA isolation and qRT–PCR

For qRT–PCR, 100,000–300,000 cells were first pre-induced for 48 h with each dox concentration in six-well plates. RNA was then isolated using the RNeasy Plus Mini Kit (Qiagen, 74134). Then, 1 µg of total RNA from each sample was converted to cDNA using iScript Reverse Transcription Supermix (Bio-Rad, 1708841). Next, qPCR reactions were set up using TaqMan Fast Advanced Master Mix (Thermo Fisher Scientific, 4444557) with TaqMan Gene Expression Assay and run using the QuantStudio 3 Real-Time PCR System (Eppendorf, A28137) in standard curve mode, using the TaqMan probes listed in Supplementary Table 4. On the other hand, customized qPCR primers for detecting BACH1t were designed using PrimerBlast (NCBI) and ordered from IDT and were verified for specificity and efficiency using serial-diluted positive and negative control DNA. The GAPDH reference primer pair was a pre-designed product from IDT (Hs.PT.39a.22214836) and was also verified for efficiency using genomic DNA control. Eventually, we quantitated BACH1t mRNA levels using the PowerUp SYBR Green Master Mix (Thermo Fisher Scientific, A25741) with GAPDH level as reference in three independent repeats. BACH1t primer sequences are listed in Supplementary Table 4.

### Immunofluorescence

In total, 300,000–500,000 cells were first pre-induced over 48 h with each dox concentration in six-well plates or T-25 flasks. Cells were next harvested by trypsin and neutralized with fresh media, followed by centrifugation at 500*g* for 5 min and vacuum aspiration of the supernatant. Cells were then fixed with 750–1,000 µl of freshly made 4% paraformaldehyde at room temperature for 15 min, followed by washing with 750–1,000 µl of PBS and centrifugation at 500*g* for 5 min. The supernatant was discarded thoroughly, and cells were resuspended for 30 min in 750–1,000 µl of ice-cold methanol at −20 °C. Then, cells were washed with 750–1,000 µl of PBS again and centrifuged at 500*g* for 5 min, followed by vacuum aspiration of the supernatant. Cell pellets were resuspended in 100 µl 1:50 BACH1 antibody (Santa Cruz Biotechnology, sc-271211 AF647) or 100 µl 1:50 RKIP antibody (Santa Cruz Biotechnology, sc-376925 AF647) diluted in incubation buffer (1× PBS and 0.5 g of BSA) and incubated for 1 h at room temperature protected from light. After incubation, cells were washed again with 500 µl of excess incubation buffer and centrifuged at 500*g* for 5 min, with the supernatant discarded. Finally, cells were fully resuspended in 500 µl of PBS and run on a BD LSRFortessa flow cytometer, collecting about 10,000 events per sample. Fluorescence readouts were collected from the red APC channel with the PMT voltage set to 350 V, and the readout of a few samples was normalized to 350 V based on voltage reference samples.

### Hemin preparation and treatment

Hemin (Sigma-Aldrich, H9039-1G) was prepared in 10 mM NaOH solution and further diluted in culturing media to the desired concentrations for cell treatment. For expression level measurement, hemin was added into cell culturing media after 48-h dox induction and maintained for 48 h together with dox before flow cytometry. For invasion measurements, hemin was added into cell culturing media after 48-h dox induction and maintained for 48 h together with dox before the Boyden chamber assays.

### Boyden chamber invasion assays

The 24-well invasion assay plates were purchased from Thermo Fisher Scientific (353097). Each Boyden chamber membrane was coated with a thin layer of 1× Basal Membrane Extract (BME) solution (diluted from 5× Basal Membrane Extract solution, Thermo Fisher Scientific, 3455-096-02) and incubated overnight at 37 °C. Cells were pre-induced at each dox concentration for 48 h and serum-starved for another 24 h while maintaining the dox concentration constant. Then, the cells were trypsinized and centrifuged at 500*g* for 5 min, followed by two rounds of PBS washes to remove any remaining serum-containing media. Then, the cells were resuspended and roughly diluted to a 0.5 × 10^6^ concentration (three replicate measurements using the Nexcelom Cellometer). Then, we seeded 100 µl of serum-free media from each suspension with 45,000–60,000 cells for each Boyden chamber, setting up three replicates in separate chambers for each dox concentration. We used 10% serum as the chemoattractant in these assays. After 16–24 h, to stain for live cells, we applied NucBlue Live ReadyProbes (Thermo Fisher Scientific, R37605) to the membranes for 2 h at 37 °C in the dark. Cells in the top chamber were removed from the membrane with a wet cotton swab. Next, cells in the bottom chamber were imaged in the DAPI/GFP/BF channels using the microscopy setup described above. We imaged five random fields within the insert using a ×10 Ph1 objective (type: CFI Plan Fluor). To calculate invasiveness, we estimated the total under-membrane area from the imaged area, using an area factor of 21.54, because each frame was 1.18 × 1.18 mm^2^, and the total area was 0.3 cm^2^. Then, we multiplied the area factor with the average cell count from five random fields to estimate the total invading cell number, which we divided by the total seeding cell number to obtain invasiveness. We estimated invasiveness for three replicates in each dox condition and presented the results as mean ± s.d.

### Proliferation assays

For proliferation assays, cells were dox induced 48 h before seeding, and 3,000–5,000 cells (depending on the cell line) were plated in 96-well plates with 12 replicates in each dox condition. Around 6 h after seeding, the first three replicates were assayed using alamarBlue HS Cell Viability Reagent (Invitrogen, A50100) for viable cells. Cells were incubated in alamarBlue reagent for 4 h, and then absorbance measurements were taken at wavelengths of 570 nm and 600 nm, with media blank control using a Tecan Infinite Pro 200 spectrophotometer. Each of the remaining three replicates was then successively measured every 24 h until 72 h endpoint. Each absorbance value was adjusted by subtracting the media blank absorbance at the same wavelength. Cell proliferation was measured as the alamarBlue reduction score (*S*) calculated as:

$$S=\left({O}_{2}\times {A}_{570}\right)-({O}_{1}\times {A}_{600})$$

where *O*_1_ and *O*_2_ are the molar extinction coefficients of oxidized alamarBlue at 570 nm and 600 nm, respectively, and *A*_570_ and *A*_600_ are the absorbances of test wells at 570 nm and 600 nm, respectively. Relative proliferation was then calculated as the fold change between the average scores of the induced wells to uninduced control wells. Cell doubling times *T*_*d*_ were calculated from the average relative proliferation fold change between timepoints 0 h and 48 h, as follows:

$${T}_{d}=({t}_{2}-{t}_{1})\ast \frac{\mathrm{ln}(2)}{\mathrm{ln}(\frac{{f}_{2}}{{f}_{1}})}$$

where *t*_2_ and *t*_1_ are the times of measurement (48 h and 0 h, respectively), and *f*_2_ and *f*_1_ are the average relative proliferation fold changes measured at times *t*_2_ and *t*_1_, respectively.

### Immunoblotting (western blotting)

The tissues were lysed in RIPA buffer supplemented with protease and phosphatase inhibitor at 5 mg ml^−1^ concentration. The supernatant containing proteins was collected after centrifuging tissue lysates at 12,000 r.p.m. at 4 °C. Protein concentration was determined by the BCA protein assay kit, and 20 µg of protein samples was boiled and loaded onto SDS–PAGE gels. The gels were transferred to 0.22-µm nitrocellulose membranes and blocked with 5% non-fat milk in 1× Tris-Buffered Saline containing 0.1% Tween 20 (TBST). The membranes were incubated with primary antibodies against BACH1 (Santa Cruz Biotechnology, sc-271211) and β-tubulin (Santa Cruz Biotechnology, sc-55529) at 4 °C overnight. After three washes (15 min, 5 min and 5 min) with 1× PBS containing 0.1% Tween 20 (PBST), the membranes were incubated with a rabbit secondary antibody conjugated with horseradish peroxidase (1:2,000) for 1 h, followed by three washes (15 min, 5 min and 5 min) with PBST. A chemiluminescence reagent kit was used to visualize protein bands with horseradish peroxidase secondary antibodies.

### Breast cancer cell line expression analysis

Raw RNA-seq data of BACH1 and RKIP were directly acquired from the cBioPortal database with its source from the Cancer Cell Line Encyclopedia. The original cell line annotation from the encyclopedia did not contain TNBC status information. So, we assigned TNBC versus non-TNBC status to the breast cancer cell lines based on the existence of ER, PR and HER2 markers with the reference to previous report. The expression distributions of both BACH1 and RKIP were arranged from high to low level with TNBC and non-TNBC subtypes separately marked. MDA-MB-231 cell status was particularly labeled in both distributions.

### TCGA and gene set enrichment analysis

We normalized RNA-seq results from TCGA BRCA samples (provisional, *n* = 1,100) directly downloaded from the cBioPortal database (https://www.cbioportal.org). Likewise, we downloaded gene lists correlated with BACH1 and RKIP, as cBioPortal already has such correlation matrices generated for the TCGA BRAC provisional set. BACH1 expression baseline was defined to be the average expression of samples in which BACH1 was diploid, and BACH1 lower and higher expression groups were classified based on the *z*-score relative to the baseline.

Functional gene set enrichment analysis (biological process enrichment analysis and molecular function enrichment) of the gene sets that correlate with BACH1 and RKIP was performed using the web-based interface of PANTHER (http://pantherdb.org). To identify processes and functions enriched in the input gene lists, we used Gene Ontology annotation categories.

### Data processing and statistical analysis

Flow cytometry data were analyzed with FlowJo software version 10 (Becton Dickinson). Forward-scatter and side-scatter gates were predefined for each cell type or assay based on the reference sample pre-tests to exclude debris. Also, a fluorescence-based gate was imposed for FACS for desired target cells. Imaging data were collected and mainly analyzed using Nikon Elements AR version 4.40.00 (Build 1084). Fiji (ImageJ 1.52a) and the Image Processing Toolbox from MATLAB (MathWorks) were also used for image processing and analysis. Most of the data plots as well as statistical analysis were generated and performed using MATLAB or GraphPad Prism 8 (GraphPad Software). Statistical details are in the figure legends, including the statistical tests used. In all figures, results are presented as mean ± s.d. unless otherwise noted in the figure legend. *P < 0.05 was considered statistically significant, as indicated by an asterisk in the figure legend.

### Computational modeling and mathematical derivations

We used MATLAB (R2020b) for computational analyses and simulations. We converted single-cell expression data to the log space by taking their log_10_ values. The flow cytometry histograms became approximately Gaussian. Following previous work^13^, we developed exact simulations of Ornstein–Uhlenbeck processes^65^ according with the means and standard deviations matching those of the log-transformed data. We simulated cell invasion by a standard Monte Carlo approach, allowing each cell to invade if a random number pulled from a standard uniform distribution was lower than the landscape value of that cell’s simulated log_10_(BACH1) levels. The number of such invading cells versus the original cell number defined the simulated invasiveness. Simulated histograms of invaded and control cells were generated by binning log_10_(BACH1) levels. For details on inferring the landscape and performing the simulations, see Supplementary Notes 1.3 and 1.5.

We used standard algebra and properties of moments for stochastic variables to derive the shifts in the mean and variance based on the Price equation. For details, see Supplementary Notes 1.4.

### Reporting summary

Further information on research design is available in the Nature Portfolio Reporting Summary linked to this article.

## Online content

Any methods, additional references, Nature Portfolio reporting summaries, source data, extended data, supplementary information, acknowledgements, peer review information; details of author contributions and competing interests; and statements of data and code availability are available at 10.1038/s41589-023-01344-z.

## Supplementary information

Supplementary Information Supplementary Figs. 1–6, Notes 1–7 and Tables 1–4.

Reporting Summary

Supplementary Data 1 Statistical source data for Supplementary Fig. 4.

## Data Availability

The authors declare that all data supporting the findings of this study are available in the article and its supplementary files. Data for the main figures and extended data figures are provided in the source data files and supplementary information files for the supplementary figures. Raw data can be accessed at https://openwetware.org/wiki/CHIP:Data. Source data are provided with this paper.

## Source data

Source Data Fig. 2 Statistical source data.
Source Data Fig. 2 Unprocessed western blots.
Source Data Fig. 3 Statistical source data.
Source Data Fig. 4 Statistical source data.
Source Data Fig. 5 Statistical source data.
Source Data Fig. 5 Unprocessed western blots.
Source Data Fig. 6 Statistical source data.
Source Data Extended Data Fig. 1 Statistical source data.
Source Data Extended Data Fig. 1 Unprocessed gels.
Source Data Extended Data Fig. 2 Statistical source data.
Source Data Extended Data Fig. 3 Statistical source data.
Source Data Extended Data Fig. 4 Statistical source data.
Source Data Extended Data Fig. 5 Statistical source data.
Source Data Extended Data Fig. 6 Statistical source data.
Source Data Extended Data Fig. 7 Statistical source data.
Source Data Extended Data Fig. 8 Statistical source data.
Source Data Extended Data Fig. 10 Statistical source data.

## Code availability

Custom MATLAB code files are available at https://github.com/gaborbalazsi/BACH1Landscape.
