# Supplementary material for: Nonmonotone invasion landscape by noise-aware control of metastasis activator levels
Source: Nat Chem Biol. 2023 May 25;19(7):887–99. doi: 10.1038/s41589-023-01344-z (PMC10299915; doi:10.1038/s41589-023-01344-z)
Supplement: Supplementary file 1 — Supplementary Figs. 1–6, Notes 1–7 and Tables 1–4. [file 41589_2023_1344_MOESM1_ESM.pdf]

nature chemical biology

Article https://doi.org/10.1038/s41589-023-01344-z

Nonmonotone invasion landscape by noise-aware control of metastasis activator levels

In the format provided by the authors and unedited

Table of Contents

Supplementary Figures 2

Supplementary Notes 8

1.1 Modeling hemin-promoted BACH1 degradation. 8

1.2 Investigation of expression noise effects on cell invasion via Positive-Feedback gene circuit 9

1.3 Cellular fitness (invasion) landscape inference 10

- 1.3.1 Local cellular fitness landscapes 10
- 1.3.2 Global consensus cellular fitness landscapes 10

1.4 Mathematical theory of phenotypic selection 11

- 1.4.1 Price Equation for the shift in mean log10(BACH1 expression) 11
- 1.4.2 Price Equation for log10(BACH1) variance 14

1.5 Computational simulations of invasion. 18

1.6 Nonmonotonicity in let-7 mediated loss of targets 19

1.7 Nonmonotonicity by generic incoherent feedforward loops 20

Supplemental Tables 21

Supplementary Figures

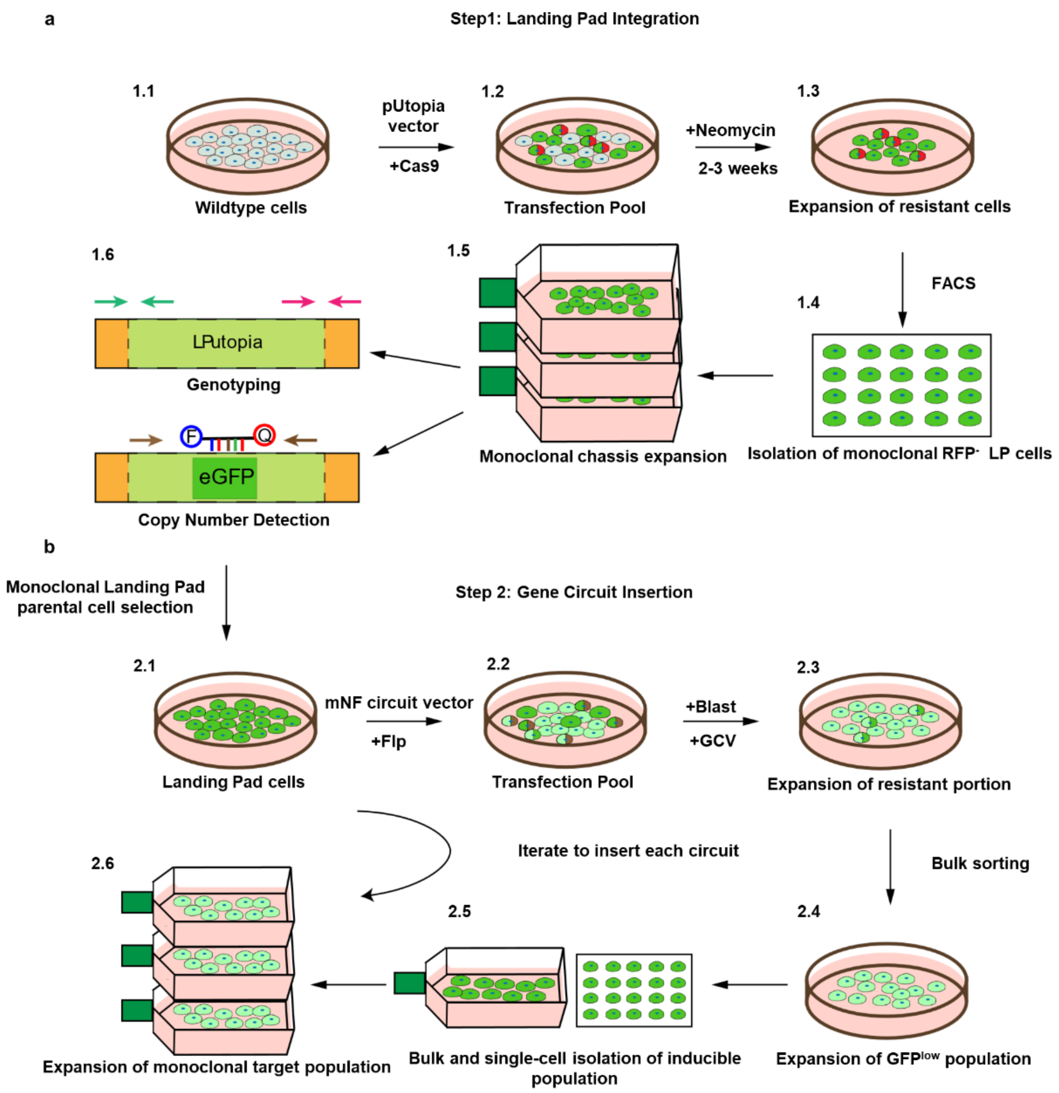

Supplementary Fig. 1 Experimental procedures of two-step integration strategy. (a) Experimental workflow for engineering *AAVS1*-integrated LP cells with CRISPR/Cas9. **(b)** Experimental workflow for integrating synthetic gene circuits into LP cells via RMCE.

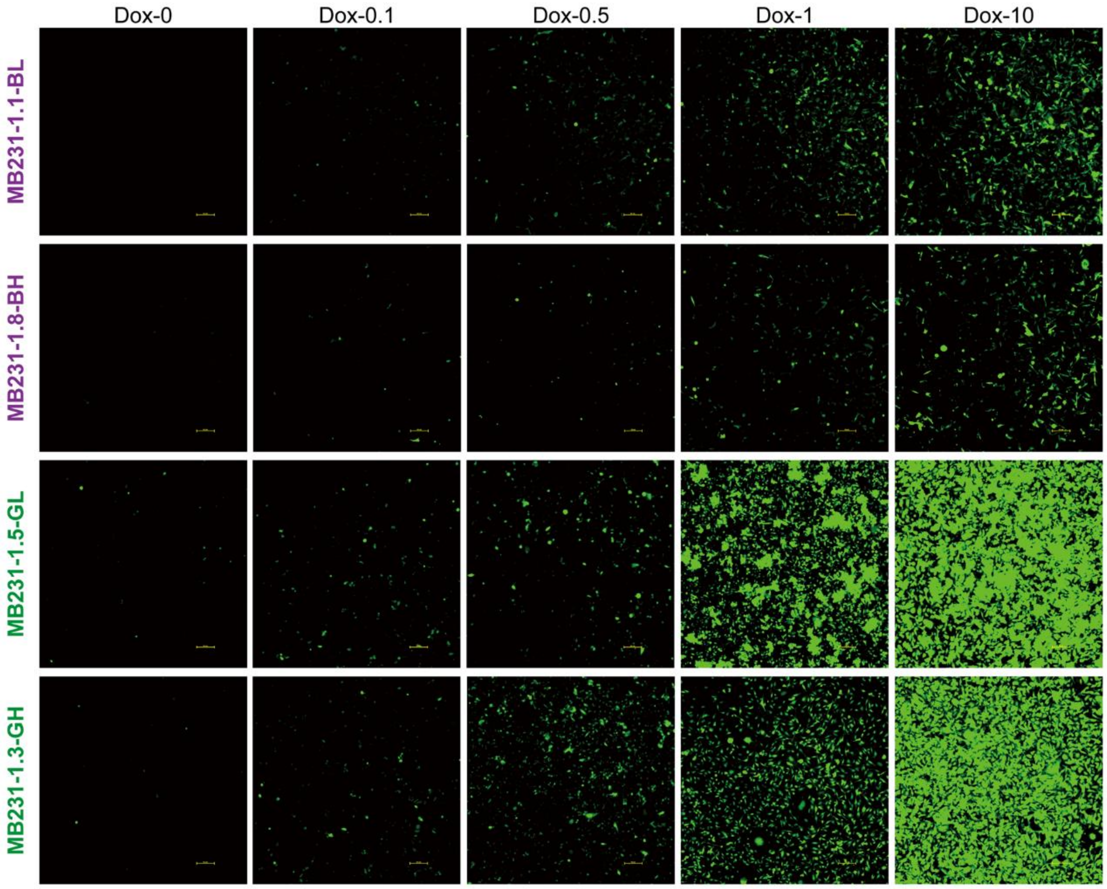

Supplementary Fig. 2 Representative dose response fluorescence microscopy images of the selected MB231 clones. Fluorescence microscopy images of all selected mNF-BACH1 and mNF-GFP clones of MB231 at 0, 0.1, 0.5, 1, and 10 ng/ml Dox induction conditions. Scale bar is 100 µm.

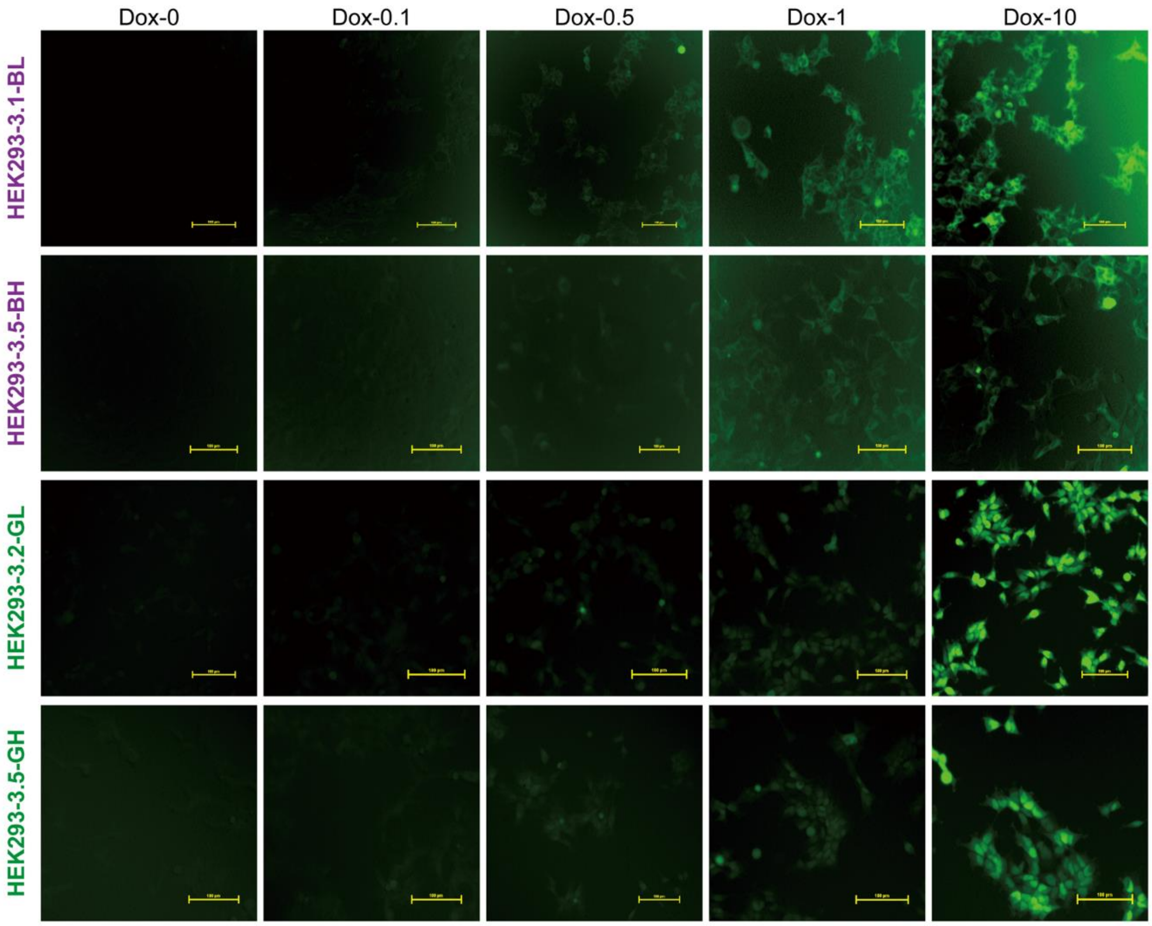

Supplementary Fig. 3 Representative dose response fluorescence microscopy images of the selected HEK293 clones. Fluorescence microscopy images of all selected mNF-BACH1 and mNF-GFP clones of HEK293 at 0, 0.1, 0.5, 1, and 10 ng/ml Dox induction conditions. Scale bar is 100 µm.

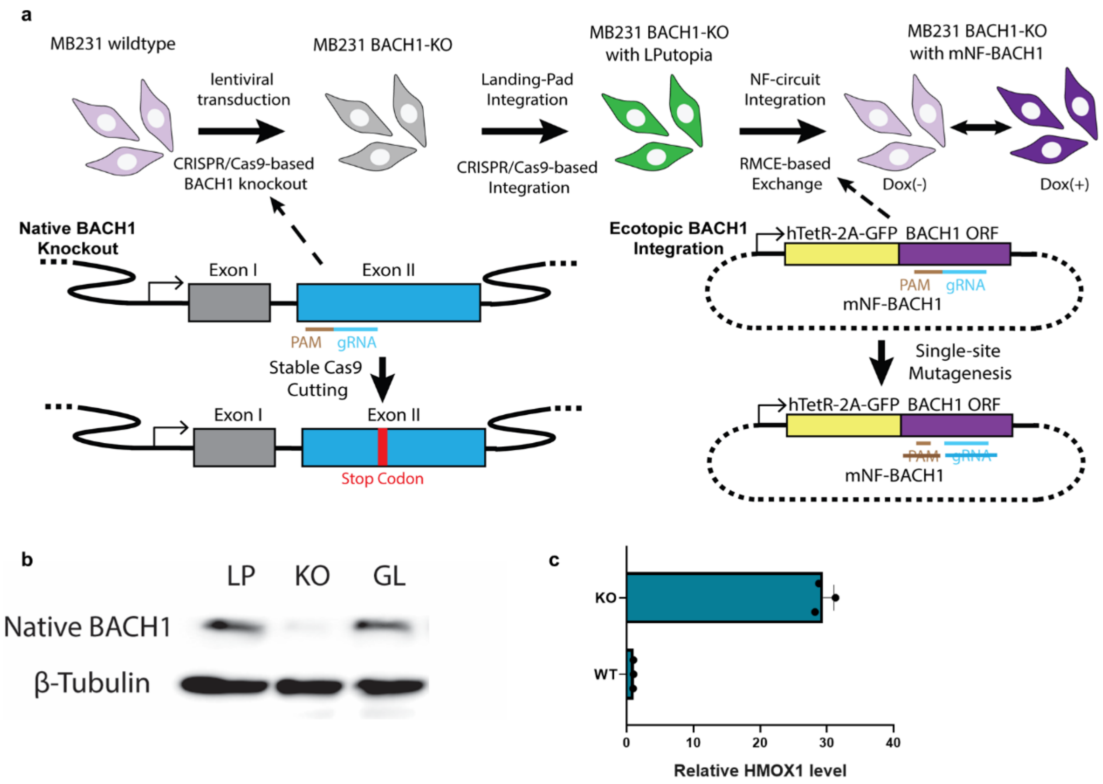

Supplementary Fig. 4 Experimental design and validation of native BACH1 knockout. (a) Experimental designs to generate native BACH1 knockout and reintroduction of circuit-regulated ectopic BACH1 copy using two-step site-specific integration strategy. **(b)** Western Blot verification of native BACH1 knockout in MB231 BK cells with reference to the MB231-1-LP parental cells and low-noise mNF-GFP MB231 clone. **(c)** qRT-PCR verification of transcriptional downstream effects to HMOX1 due to native BACH1 knockout.

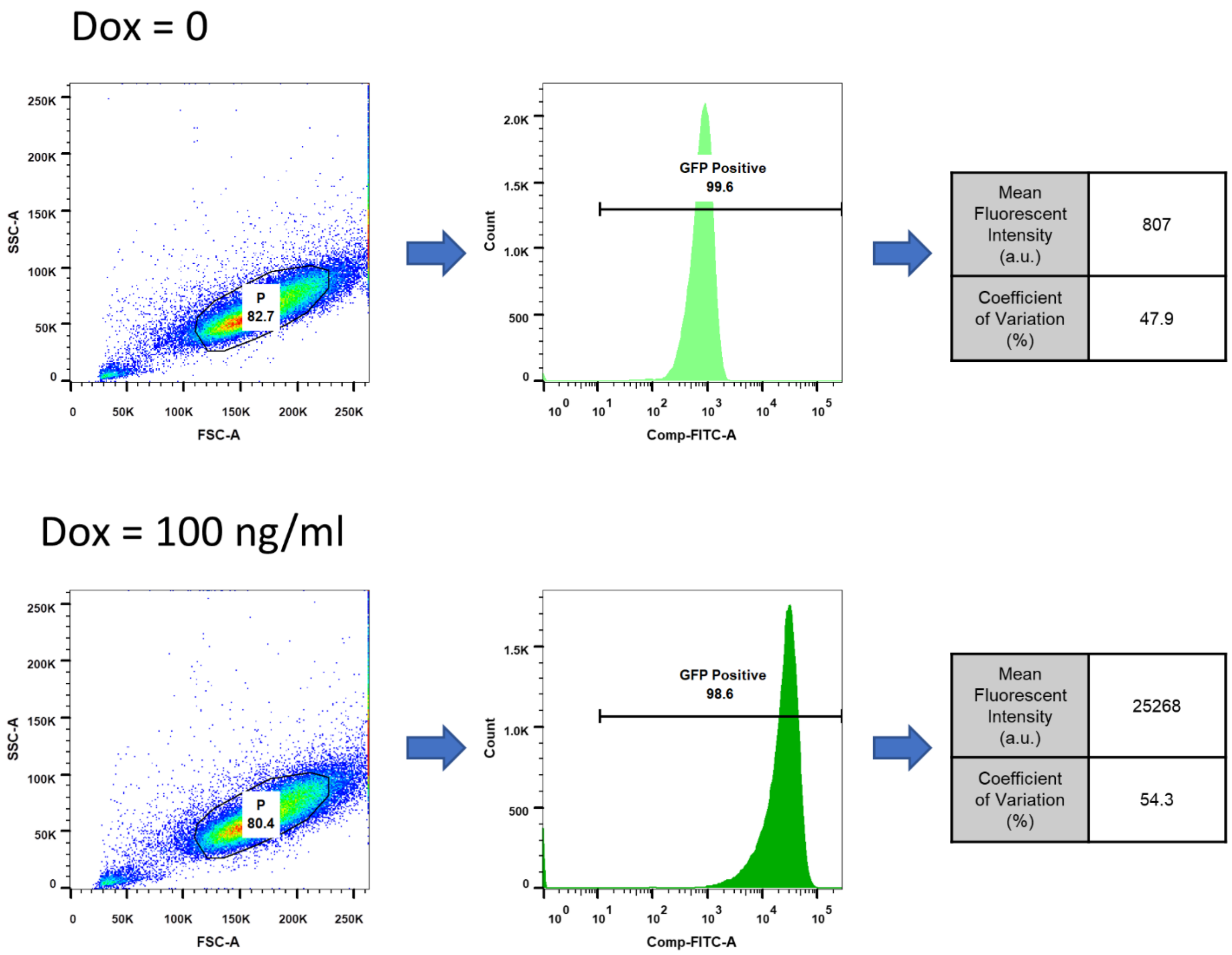

Supplementary Fig. 5 Events were gated to reduce false events in flow cytometry analysis.

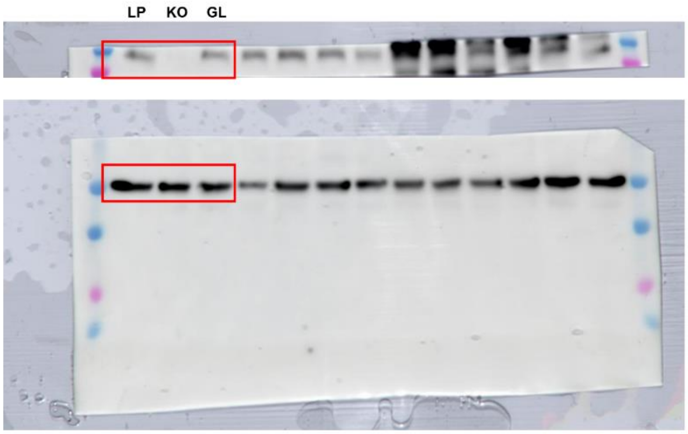

Supplementary Fig. 6 Unprocessed Western Blot images for Supplementary figure 4.

Supplementary Notes

1.1 Modeling hemin-promoted BACH1 degradation.

BACH1's concentration kinetics in mNF cells can be described by the following equation: $\frac{dB}{dt} = k - gB$, where k is BACH1 synthesis, g is BACH1 degradation and dilution.

Consequently, BACH1's steady state level will be $B_0 = \frac{k}{g}$.

When bound to BACH1 in a complex C, hemin will cause BACH1 degradation with a rate h. Denoting the rate of hemin-BACH1 association and separation by a and s, respectively, the equilibrium amounts of free and hemin-bound BACH1 will be:

$F = \frac{sB}{s + aH}$ and $C = \frac{aHB}{s + aH}$, with kinetics described by:

$\frac{dF}{dt} = k\frac{s}{s + aH} - gF$ and $\frac{dC}{dt} = k\frac{aH}{s + aH} - (g + h)C$.

The overall, total BACH1 kinetics will be: $\frac{dB}{dt} = k - gB - hC = k - \left(g + h\frac{aH}{s + aH}\right)B$.

BACH1's new steady state will be: $B_H = \frac{k}{g + h\frac{aH}{s + aH}} = B_0 \frac{\frac{s}{a} + H}{\frac{s}{a} + \left(1 + \frac{h}{g}\right)H}$.

We fit this formula to experimental data and obtained $\frac{s}{a} \approx 25.17$ and $\frac{h}{g} \approx 7.8867$.

Therefore, hemin's equilibrium binding constant is 25.17 per unit concentration and the degradation rate due to hemin is almost 8 times the normal rate of BACH1 degradation.

1.2 Investigation of expression noise effects on cell invasion via Positive-Feedback gene circuit

To investigate more broadly the effects of expression noise, we used the same two-step strategy to integrate a BACH1-controlling mammalian Positive Feedback (mPF-BACH1) gene circuit into the genome of MB231 cells. Contrasting a Chinese hamster cell line, eGFP::BACH1 expression in mPF-MB231 cells was bimodal above an induction threshold of ~100 ng/ml Dox (**Extended Data Fig.5c**). Higher Dox levels enriched the high-expression peak at the expense of the low-expression peak, while the positions of both peaks remained unchanged, causing a dose-dependent increase of eGFP::BACH1 expression mean (**Extended Data Fig.5d**). Such a bimodal distribution corresponds to two drastically different phenotypes, another noise effect that is different from the broad phenotypic distribution in high-noise clones. To study how bimodal expression affects invasion, we identified four induction levels where the expression means of mPF-BACH1 and mNF-BACH1 cells were similar while their CVs were drastically different (**Extended Data Fig.5e-f)**. We predicted mPF cell population invasiveness by averaging the invasiveness corresponding to the mean of low- mid- and high-expressing mPF-BACH1 cells, weighted by the estimated subpopulation sizes **(Extended Data Fig.5g)**. This analysis indicated that unlike for other gene circuits, mPF invasiveness should increase in a monotone fashion because of the progressive enrichment of the high-expressing subpopulation at the expense of the low-expressing cells, corresponding to constant low and high invasiveness on the landscape, respectively. Indeed, invasion assays confirmed the prediction **(Extended Data Fig.5h)**. Also, high noise in mPF cells still promoted invasion at high BACH1 expression.

1.3 Cellular fitness (invasion) landscape inference

1.3.1 Local cellular fitness landscapes

We inferred local cellular fitness landscapes based on log(eGFP::BACH1) fluorescence histograms from the invasion assays using Boyden chambers. We used identical sets of bins numbered $1 \le i \le M$ for estimating fluorescence histograms of seeded (p_i) and invading (q_i) cells as probability distributions, so $\sum_{i=1}^{M} p_i = \sum_{i=1}^{M} q_i = 1$. Then, for each Dox induction level, we rescaled the height of all bins for the invaded cells by the average invasiveness $\langle w \rangle_{Dox}$, so q_i a $\langle w \rangle_{Dox} q_i$ and $\sum_{i=1}^{M} q_i = \langle w \rangle_{Dox}$. Next, we estimated the local invasiveness as $w_i(Dox) = \frac{q_i}{p_i}$ within each bin i to construct the local fitness landscape at each Dox concentration. Finally, considering that both p_i and q_i are noisy, we used smoothing and then error propagation models to cut off regions with error CVs larger than some threshold.

1.3.2 Global consensus cellular fitness landscapes

We constructed global cellular fitness landscapes by weighted averaging of local fitness landscapes. The local fitness landscapes $w_i(Dox)$ inferred at different Dox concentrations show similar trends, but do not agree. Therefore, for each bin $1 \le i \le M$ we calculated the weighted average of local fitness landscapes as $W_i = \sum_{Dox} r_i w_i(Dox)$.

We calculated the weights for each local invasiveness as $r_i(Dox) = \frac{1}{CV_i} = \frac{\mu_i}{\sigma_i}$. Based on Holmes & Buhr, *Clin. Biochem*. 40:728 (2007), errors for ratios of two quantities p_i and q_i propagate approximately as: $CV_i = \frac{\sigma_i}{\mu_i} = \frac{\sqrt{CV_p^2 + CV_q^2 + 3CV_p^2 CV_q^2 + 8CV_q^2}}{1 + CV_q^2}$, where CV_p and CV_p are the coefficients of variation of p_i and q_i, respectively.

1.4 Mathematical theory of phenotypic selection

1.4.1 Price Equation for the shift in mean log10(BACH1 expression)

First, we rederive the known relationship for the shift $\Delta\bar{z}$ due to selection in the average "character" $\bar{z} \equiv \langle z \rangle$, which is the mean of $\log_{10}$(BACH1 protein expression level) in our case.

Assume there are M groups (fluorescence bins) labeled $i = 1, 2, ..., M$, each of which contains n_i cells sharing a property z_i, $i = 1, 2, ..., M$ and fitness w_i, $i = 1, 2, ..., M$. Here, z_i represents discretized $\log_{10}$(BACH1 level), with the same z_i value assigned to all cells within fluorescence bin i. Cells in bin i contribute to the post-selection (i.e., post-invasion) population according to their fitness w_i, being replaced by their $n_i' = n_i w_i$ "descendants", such that $w_i > 1$ implies net gain, $w_i = 1$ implies no population change, and $0 \le w_i < 1$ implies net loss of cells within bin i. Thus, the total number of cells in the post-invasion population will be: $N' = \sum_{i=1}^{M} n_i w_i$.

So far, we assumed that cells do not change their BACH1 level z_i during invasion, which corresponds to perfect inheritance (cellular memory). To make the model more realistic, we assume a z_i-changing mechanism by bin switching. Specifically, when inheritance of the trait z is imperfect, all cells within the first K bins out of M total bins are reassigned to the same K bins somehow after selection, so generally $z_j' \ne z_j$ for the bins numbered $j = 1, 2, ...K$, whereas $z_j' = z_j$ for the bins numbered $j = K+1, K+2, ..., M$. We note that the bins can always be arbitrarily renumbered so that the cells within the first K bins will be switching bins. The values $K = 0$ and $K = M$ then correspond to perfect inheritance and no inheritance, respectively.

If we denote the probability of being in bin i by $p_i = \frac{n_i}{\sum_{i=1}^{M}(n_i)} = \frac{n_i}{N}$, the average fitness will be:

$$\bar{w} = \langle w \rangle = \frac{\sum_{i=1}^{M} n_i w_i}{\sum_{i=1}^{M} n_i} = \sum_{i=1}^{M} (p_i w_i) .$$

Likewise, the average of the $\log_{10}$(BACH1 level) property z_i, prior to invasion will be:

$$\bar{z} \equiv \langle z \rangle = \frac{\sum_{i=1}^{M} n_i z_i}{\sum_{i=1}^{M} n_i} = \sum_{i=1}^{M} (p_i z_i) . \qquad (1)$$

After invasion, the average post-invasion property $\bar{z}'$ will be:

$$\bar{z}' = \frac{\sum_{i=1}^{M}[(n_i w_i) z_i']}{\sum_{i=1}^{M}(n_i w_i)} = \frac{\sum_{i=1}^{K}[(n_i w_i) z_i'] + \sum_{i=K+1}^{M}[(n_i w_i) z_i]}{\sum_{i=1}^{M}(n_i w_i)}$$

By subtracting and adding the same sum to the numerator, and regrouping terms, we have:

$$\bar{z}' = \frac{\sum_{i=1}^{M}[(n_i w_i)(z_i' - z_i)] + \sum_{i=1}^{M}[(n_i w_i) z_i]}{\sum_{i=1}^{M}(n_i w_i)} . \qquad (2)$$

With simplified notation, the average post-invasion property $\bar{z}'$ can also be written as:

$$\bar{z}' = \frac{\langle w_i (z_i' - z_i)\rangle + \langle w_i z_i \rangle}{\bar{w}} = \frac{\langle w_i z_i' \rangle}{\bar{w}} .$$

Using Equations (1) and (2), the difference in average character can be written as:

$$\Delta\bar{z} = \bar{z}' - \bar{z} = \frac{\sum_{i=1}^{M}[(n_i w_i)(z_i' - z_i)] + \sum_{i=1}^{M}[(n_i w_i) z_i]}{\sum_{i=1}^{M}(n_i w_i)} - \frac{\sum_{i=1}^{M}[n_i z_i]}{\sum_{i=1}^{M} n_i} = \frac{\langle w_i z_i' \rangle}{\bar{w}} - \bar{z} .$$

We now use some algebra to rewrite this expression.

$$\Delta\bar{z} = \bar{z}' - \bar{z} = \frac{\langle w_i z_i' \rangle}{\bar{w}} - \bar{z} = \frac{\langle w_i z_i' \rangle - \bar{z}\,\bar{w}}{\bar{w}} = \frac{\langle w_i (z_i' - z_i)\rangle + \langle w_i z_i \rangle - \langle z \rangle \langle w \rangle}{\bar{w}} = \frac{\langle w_i \Delta z_i \rangle}{\bar{w}} + \frac{Cov(w,z)}{\bar{w}}$$

Therefore, the Price equation for the change in average BACH1 levels upon invasion is:

$$\bar{w}\Delta\bar{z} \equiv \bar{w}(\bar{z}' - \bar{z}) = Cov(w,z) + \langle w_i \Delta z_i \rangle \text{, or } \Delta\bar{z} = \frac{Cov(w,z) + \langle w_i \Delta z_i \rangle}{\bar{w}} . \qquad (3)$$

Next, we discuss two cases: (i) without selection; and (ii) with selection.

(i) Without selection BACH1 levels are uncorrelated with fitness, so $Cov(w,z)=0$. The only remaining term in (3) is $\Delta\bar{z}=\frac{\langle w_i\Delta z_i\rangle}{\bar{w}}$. We show that this term will vanish, so $\Delta\bar{z}\equiv 0$.

a. First, with perfect cellular memory, $\Delta z_i=0$, so $\Delta\bar{z}=0$.
b. Second, without any memory, all +/- Δz_i shifts can occur (due to random bin reassignments), so $\langle\Delta z_i\rangle$ must average to 0, and $\langle w_i\Delta z_i\rangle=\langle w_i\rangle\langle\Delta z_i\rangle=0$.
c. Third, with intermediate memory, we have a mixture of subcases (i)a and (i)b.

(ii) With selection BACH1 levels correlate with fitness, $Cov(w,z)\neq 0$.

a. First, with perfect cellular memory, $\Delta z_i=0$, so $\langle w_i\Delta z_i\rangle=0$ and we obtain the covariance equation: $\Delta\bar{z}=\frac{Cov(w,z)}{\bar{w}}$.
b. Second, without inheritance, all z_i cells redistribute randomly across the bins, so: $\langle w\Delta z\rangle=\langle w_i(z_i'-z_i)\rangle=\langle w_iz_i'\rangle-\langle w_iz_i\rangle=Cov(w_i,z_i')-Cov(w_i,z_i)+\langle w_i\rangle(\langle z_i'\rangle-\langle z_i\rangle)$. As discussed above, $\Delta\bar{z}=0$. Also, $Cov(w_i,z_i')=0$, so $\langle w\Delta z\rangle=-Cov(w_i,z_i)$.
c. Third, with intermediate memory, the terms in the denominator will be nonzero, allowing selection, to degree between cases (ii)a and (ii)b: $0<\Delta\bar{z}<\frac{Cov(w,z)}{\bar{w}}$.

Linear (directional) selection: We compute the shift $\Delta\bar{z}$ with perfect memory and $w_i=az_i+b$.

$$\Delta\bar{z}=\frac{Cov(w,z)}{\langle w\rangle}=\frac{\langle w_iz_i\rangle-\langle w\rangle\langle z\rangle}{\bar{w}}=\frac{\langle(az_i+b)z_i\rangle-\langle az_i+b\rangle\langle z\rangle}{\bar{w}}=\frac{a\langle z_i^2\rangle-a\bar{z}^2+b\langle z_i\rangle-b\bar{z}}{\bar{w}}$$

$$\Delta\bar{z}=\frac{aVar(z)}{\bar{w}}=\frac{aVar(z)}{a\bar{z}+b}.$$

Thus, with perfect memory, on a linear invasion landscape, the mean of z *increases* (upslope, a>0) or *decreases* (downslope, a<0) proportional to the landscape's slope, a & variance $Var(z)$.

Quadratic (divergent or disruptive) selection: With perfect memory & $w_i=az_i^2+bz_i+c$ we have:

$$\Delta\bar{z}=\bar{z}'-\bar{z}=\frac{Cov(w,z)}{\langle w\rangle}=\frac{\langle w_iz_i\rangle-\langle w\rangle\langle z\rangle}{\bar{w}}=\frac{\langle(az_i^2+bz_i+c)z_i\rangle-\langle az_i^2+bz_i+c\rangle\langle z\rangle}{\bar{w}}$$

$$\Delta\bar{z}=\frac{aCov(z,z^2)+bVar(z)}{\bar{w}}=\frac{aCov(z,z^2)+bVar(z)}{a\langle z^2\rangle+b\bar{z}+c}$$. For Gaussian z, $\Delta\bar{z}=\frac{[2a\bar{z}+b]Var(z)}{a\langle z^2\rangle+b\bar{z}+c}$.

Thus, the mean of z *increases* if $2az+b>0$ or *decreases* if $2az+b<0$, proportional to the fitness derivative [$2az+b$] and the covariance $Cov(z^2,z)$.

1.4.2 Price Equation for log10(BACH1) variance

Again, we assume that there are there are M groups (fluorescence bins), each bin containing n_i cells that share the same $\log_{10}$(BACH1 level) property z_i, $i = 1, 2, ..., M$ and fitness w_i, $i = 1, 2, ..., M$. The cells within each bin i contribute $n_i' = n_i w_i$ cells to the same bin in the post-invasion population, which for invasion assays are a subset of the original n_i cells since $w_i \le 1$.

Therefore, the total number of invading cells will be: $N' = \sum_{i=1}^{M} n_i w_i$. Inheritance of the trait z may be imperfect, so $z_j' \ne z_j$ for bins $j = 1, 2, ...K$, and $z_i' = z_i$ for bins $j = K+1, K+2, ..., M$.

The variance of property z_i in the pre-invasion cell population will be:

$$Var(z) = \frac{\sum_{i=1}^{M}[n_i z_i^2]}{\sum_{i=1}^{M} n_i} - \left\{ \frac{\sum_{i=1}^{M}[n_i z_i]}{\sum_{i=1}^{M} n_i} \right\}^2 = \left\langle z^2 \right\rangle - \left\langle z \right\rangle^2 . \quad (4)$$

The variance of property z_i after invasion will be:

$$Var(z') = \frac{\sum_{i=1}^{M}[(n_i w_i) z_i'^2]}{\sum_{i=1}^{M}(n_i w_i)} - \left\{ \frac{\sum_{i=1}^{M}[(n_i w_i) z_i']}{\sum_{i=1}^{M}(n_i w_i)} \right\}^2 = \frac{\left\langle w z'^2 \right\rangle}{\bar{w}} - \left\{ \frac{\left\langle w z' \right\rangle}{\bar{w}} \right\}^2 . \quad (5)$$

Combining Equations (4) and (5), the change in variance will be:

$$\Delta Var(z) = Var(z') - Var(z) = \frac{\left\langle w z'^2 \right\rangle}{\bar{w}} - \frac{\left\langle w z' \right\rangle^2}{\bar{w}^2} - \left\langle z^2 \right\rangle + \left\langle z \right\rangle^2$$

$$\Delta Var(z) = \frac{\left\langle w z'^2 \right\rangle - \bar{w}\left\langle z^2 \right\rangle}{\bar{w}} - \frac{\left\langle w z' \right\rangle^2 - \left\langle w \right\rangle^2 \left\langle z \right\rangle^2}{\bar{w}^2}$$

$$\Delta Var(z) = \frac{\left\langle w z^2 \right\rangle - \left\langle w \right\rangle \left\langle z^2 \right\rangle + \left\langle w (z'^2 - z^2) \right\rangle}{\bar{w}} - \frac{\left\langle w z' \right\rangle^2 - \left\langle w z \right\rangle^2 + \left\langle w z \right\rangle^2 - \left\langle w \right\rangle^2 \left\langle z \right\rangle^2}{\bar{w}^2}$$

$$\Delta Var(z)=\frac{Cov(w,z^2)+\langle w(z'^2-z^2)\rangle}{\bar{w}}-\frac{\{\langle wz'\rangle-\langle wz\rangle\}\{\langle wz'\rangle+\langle wz\rangle\}+\{\langle wz\rangle-\langle w\rangle\langle z\rangle\}\{\langle wz\rangle+\langle w\rangle\langle z\rangle\}}{\bar{w}^2}$$

$$\Delta Var(z)=\frac{Cov(w,z^2)+\langle w(z'^2-z^2)\rangle}{\bar{w}}-\frac{\langle w(z'-z\rangle\langle w(z'+z)\rangle+\left[\langle wz\rangle+\langle w\rangle\langle z\rangle\right]Cov(w,z)}{\bar{w}^2}$$

$$\Delta Var(z)=\frac{Cov(w,z^2)+\langle w\Delta z(z'+z)\rangle}{\bar{w}}-\frac{\langle w\Delta z\rangle\langle w(z'+z)\rangle+\left[\langle wz\rangle+\langle w\rangle\langle z\rangle\right]Cov(w,z)}{\bar{w}^2}$$

In the following calculations, we assume perfect inheritance, $z_i'=z_i$, so $\Delta z=0$. This implies:

$$\Delta Var(z)=\frac{Cov(w,z^2)}{\bar{w}}-\frac{\left[\langle wz\rangle+\langle w\rangle\langle z\rangle\right]Cov(w,z)}{\bar{w}^2}=\Delta_1 Var(z)-\Delta_2 Var(z) \qquad (6)$$

Note that with symmetric fitness peaks/valleys & z_i, $Cov(w_i,z_i)=0$, so $\Delta Var(z)=\frac{Cov(w,z^2)}{\bar{w}}$.

<u>(i.) Directional selection (linear landscape).</u>

We assume $w_i=az_i+b$, and compute $\Delta Var(z)$ term by term. The first term in (6) is:

$$\bar{w}\Delta_1 Var(z)=Cov(w_i,z_i^2)=\langle w_i z_i^2\rangle-\langle w_i\rangle\langle z_i^2\rangle=\langle(az_i+b)z_i^2\rangle-\langle az_i+b\rangle\langle z_i^2\rangle=$$

$$=a\langle z_i^3\rangle+b\langle z_i^2\rangle-a\langle z_i\rangle\langle z_i^2\rangle-b\langle z_i^2\rangle=a\langle z_i^3\rangle-a\langle z_i\rangle\langle z_i^2\rangle=aCov(z,z^2)$$, so:

$\Delta_1 Var(z)=a\frac{Cov(z,z^2)}{\bar{w}}=\frac{aCov(z,z^2)}{a\bar{z}+b}$. For Gaussian z_i, $\Delta_1 Var(z)=\frac{2a\langle z\rangle Var(z)}{a\langle z\rangle+b}$.

The second term in (6) is:

$$\bar{w}^2\Delta_2 Var(z)=\left[\langle w_i z_i\rangle+\bar{z}\,\bar{w}\right]Cov(w_i,z_i)=\left[\langle(az_i+b)z_i\rangle+\bar{z}\,\langle az_i+b\rangle\right]Cov(az_i+b,z_i)=$$

$$=\left[a\langle z_i^2\rangle+b\langle z_i\rangle+a\bar{z}\,\langle z_i\rangle+b\bar{z}\right]\left[\langle(az_i+b)(z_i)\rangle-\langle az_i+b\rangle\langle z_i\rangle\right]=$$

$$=\left[a\langle z_i^2\rangle+a\,\bar{z}^2+2b\bar{z}\right]\left[a\langle z_i^2\rangle+b\bar{z}-a\bar{z}^2-b\bar{z}\right]=aVar(z)\left[a\langle z_i^2\rangle-a\,\bar{z}^2+2a\,\bar{z}^2+2b\bar{z}\right]=$$

$$=a^2Var^2(z)+2a\bar{z}Var(z)(a\,\bar{z}+b)=a^2Var^2(z)+2a\bar{z}\,\bar{w}Var(z).$$

Overall, $\Delta Var(z)=\Delta_1 Var(z)-\Delta_2 Var(z)=\frac{a}{\bar{w}}\left[Cov(z,z^2)-2\bar{z}Var(z)\right]-\left(\frac{a}{\bar{w}}\right)^2 Var^2(z)$.

For Gaussian z_i, $Cov(z,z^2)=2\bar{z}Var(z)$, so $\Delta Var(z)=-\left(\frac{a}{\bar{w}}\right)^2 Var^2(z)$.

Thus, the trait variance always *decreases* on any non-flat invasion landscape, by a factor proportional to the slope a, times the square variance of the trait.

(ii.) Divergent or stabilizing selection (quadratic landscape).

We assume: $w_i = az_i^2 + bz_i + c$. We calculate the terms of $\Delta Var(z)=\Delta_1 Var(z)-\Delta_2 Var(z)$:

$$\bar{w}\Delta_1 Var(z)=Cov(w_i,z_i^2)=\left\langle (az_i^2+bz_i+c)z_i^2\right\rangle-\left\langle (az_i^2+bz_i+c)\right\rangle\left\langle z_i^2\right\rangle=$$

$$=a\left\langle z_i^4\right\rangle+b\left\langle z_i^3\right\rangle+c\left\langle z_i^2\right\rangle-a\left\langle z_i^2\right\rangle\left\langle z_i^2\right\rangle-b\left\langle z_i\right\rangle\left\langle z_i^2\right\rangle-c\left\langle z_i^2\right\rangle=$$

$$=aVar(z^2)+bCov(z,z^2)$$

For Gaussian z_i, $\Delta_1 Var(z)=\frac{aVar(z^2)+bCov(z,z^2)}{\bar{w}}=\frac{aVar(z^2)+2b\bar{z}Var(z)}{\bar{w}}$.

The second term in (6) is:

$$\bar{w}^2\Delta_2 Var(z)=\left[a\left\langle z_i^3\right\rangle+a\left\langle z_i^2\right\rangle\left\langle z_i\right\rangle+b\left\langle z_i^2\right\rangle+b\left\langle z_i\right\rangle^2+2c\left\langle z_i\right\rangle\right]\left[a\left\langle z_i^3\right\rangle-a\left\langle z_i^2\right\rangle\left\langle z_i\right\rangle+b\left\langle z_i^2\right\rangle-b\left\langle z_i\right\rangle^2\right]$$

$$\bar{w}^2\Delta_2 Var(z)=\left[aCov(z,z^2)+bVar(z)+2\bar{z}\left(a\left\langle z_i^2\right\rangle+b\left\langle z_i\right\rangle+c\right)\right]\left[aCov(z,z^2)+bVar(z)\right]$$

$$\bar{w}^2\Delta_2 Var(z)=\left[aCov(z,z^2)+bVar(z)+2\bar{z}\,\bar{w}\right]\left[aCov(z,z^2)+bVar(z)\right]$$

$$\bar{w}^2\Delta_2 Var(z)=2\bar{z}\,\bar{w}\left[aCov(z,z^2)+bVar(z)\right]+\left[aCov(z,z^2)+bVar(z)\right]^2$$

$$\Delta_2 Var(z)=2\bar{z}\frac{aCov(z,z^2)+bVar(z)}{\bar{w}}+\left[\frac{aCov(z,z^2)+bVar(z)}{\bar{w}}\right]^2$$

Overall, $\Delta Var(z)=\frac{a}{\bar{w}}\left[Var(z^2)-2\bar{z}Cov(z,z^2)\right]-\left[\frac{aCov(z,z^2)+bVar(z)}{\bar{w}}\right]^2$.

Using the calculations in the Addendum, for Gaussian z_i, we have:

$$\Delta Var(z)=\left[\frac{2a}{\bar{w}}-\left(\frac{2a\mu+b}{\bar{w}}\right)^2\right]\sigma^4 .$$

Noting that the derivative $\frac{d\bar{w}}{dz}=2a\bar{z}+b=0$ at fitness peaks/valleys, $\Delta Var(z)=\frac{2a\sigma^4}{\bar{w}}$.

Thus, the trait variance *increases* at fitness valleys, $a>0$ while it *decreases* at fitness peaks, $a<0$ by a factor proportional to the landscape curvature a, times the variance of the square trait.

We used these formulas to estimate the changes in the mean and CV of $\log_{10}$(BACH1) levels at each induction level in **Extended Data Fig. 9**. Using the inferred cellular invasion landscapes, we estimated the linear slope and quadratic curvature by linear and quadratic fits, respectively, within a standard deviation centered at the current mean of $\log_{10}$(BACH1). Thus, we generated the theoretical data points in **Extended Data Fig. 9**.

We note that the real invasion landscape is neither linear nor quadratic over the expression range of the seeded cells, so exact quantitative agreement of these theoretical predictions with experimental results is not expected. The direction of change (up/down) for the mean predicted by this theory tend to agree well with simulations and experiments.

Addendum: Some calculations used above for Gaussian distributions, $\sigma N + \mu$

In the above derivations we used two relationships for Gaussian distributions, $z = \sigma N + \mu$.

First,

$$Cov(z,z^2) = Cov[\sigma n + \mu, (\sigma n + \mu)^2] = Cov[\sigma n + \mu, \sigma^2 n^2 + 2\mu\sigma n + \mu^2]$$

$$Cov(z,z^2) = Cov[\sigma n + \mu, \sigma^2 n^2] + Cov[\sigma n + \mu, 2\mu\sigma n] + Cov[\sigma n + \mu, \mu^2]$$

$$Cov(z,z^2) = Cov[\sigma n, \sigma^2 n^2] + Cov[\mu, \sigma^2 n^2] + Cov[\sigma n, 2\mu\sigma n] + Cov[\mu, 2\mu\sigma n]$$

$$Cov(z,z^2) = \sigma^3 Cov[n,n^2] + 2\mu\sigma^2 Cov[n,n] = \sigma^3 0 + 2\mu\sigma^2 1 = 2\mu\sigma^2 .$$

Second,

$$Var(z^2) = Var[(\sigma n + \mu)^2] = Var[\sigma^2 n^2 + 2\mu\sigma n + \mu^2]$$

$$Var(z^2) = Var[\sigma^2 n^2 + 2\mu\sigma n]$$

$$Var(z^2) = Var[\sigma^2 n^2] + Var[2\mu\sigma n] + 2Cov[\sigma^2 n^2, 2\mu\sigma n],$$

$$Var(z^2) = \sigma^4 Var[n^2] + 4\mu^2\sigma^2 Var[n] + 4\mu\sigma^3 Cov[n^2, n]$$

$$Var(z^2) = 2\sigma^4 + 4\mu^2\sigma^2 = 2\sigma^2[\sigma^2 + 2\mu^2].$$

Above we have used: $Var(X+Y) = Var[X] + Var[Y] + 2Cov[X,Y]$.

Also, we note that for χ_1^2 chi-square distributed Z, we have:

$\langle Z \rangle = \langle n^2 \rangle = 1$ and $Var(Z) = Var(n^2) = 2$.

1.5 Computational simulations of invasion.

We developed stochastic simulations based on methods described by Daniel A. Charlebois, Nezar Abdennur, and Mads Kærn in Phys. Rev. Lett. 107, 218101 (14 November 2011). We modeled the fluctuating BACH1 levels of individual cells using the exact simulation method for the OU process as described by Daniel T. Gillespie in “Exact numerical simulation of the Ornstein-Uhlenbeck process and its integral”, Phys. Rev. E 54, 2084 (1 August 1996).

Specifically, we assume that as time progresses, each cell’s $\log_{10}$(BACH1) levels denoted as $x(t)$ are a solution of the Ornstein-Uhlenbeck Langevin Equation:

$x(t+dt) = x(t) + \frac{1}{\tau}[\mu - x(t)]dt + c^{\frac{1}{2}} N(t)(dt)^{\frac{1}{2}}$, where $N(t)$ is a temporally uncorrelated standard normal random variable; whereas the constants μ, c, and τ are the mean, the diffusion constant, and the relaxation time, respectively.

We chose to model $\log_{10}$(BACH1) levels because the flow cytometry distributions were approximately lognormal. Thus, $\log_{10}$(BACH1) levels should be normally distributed, matching the solution of the Ornstein-Uhlenbeck process.

The $\log_{10}$(BACH1) levels can be computationally simulated to progress in time according to the following exact updating formula:

$x(t+\Delta t) = \mu + [x(t) - \mu]e^{-\frac{\Delta t}{\tau}} + \left[\frac{c\tau}{2}\left(1 - e^{-\frac{2\Delta t}{\tau}}\right)\right]^{\frac{1}{2}} n(t)$, where $n(t)$ is a single sample from a normal random variable generator, while μ, c and τ are constants.

Considering the long-term (stationary) statistics of this stochastic process: $\langle x(t) \rangle \to \mu$ and $Var\{x(t)\} \to \frac{c\tau}{2}$, the standard deviation is: $\sigma = \sqrt{\frac{c\tau}{2}}$, so the updating formula becomes:

$$x(t+\Delta t) = \mu + [x(t) - \mu]e^{-\frac{\Delta t}{\tau}} + \left[\sigma^2\left(1 - e^{-\frac{2\Delta t}{\tau}}\right)\right]^{\frac{1}{2}} n(t).$$

To model the effects of selection on cells, we calculated the mean and CV of $\log_{10}$(BACH1) levels for all experimental data, and then simulated BACH1 level fluctuations in 5000 cells using the above updating formula. We ignored cell division for simplicity. At every time step, each seeded cell could invade with a probability $\alpha w[x(t)]$, where $w[x]$ is inferred cellular invasiveness vs. $\log_{10}$(BACH1) levels, i.e., the cellular fitness landscape.

We allowed $\log_{10}$(BACH1) levels to fluctuate in both seeded and invading cells. After 500 timesteps of 0.1 hours (50 hours total simulation time), we measured the statistics and numbers of seeded and invading cells to generate the simulated data.

1.6 Nonmonotonicity in let-7 mediated loss of targets

We used a system of ODEs to model the RKIP-BACH1 system in Lee et al., PNAS 2014. In that study, we model RKIP protein (R) repression by ectopic (βE) and native (βB) BACH1 protein:

$$\frac{dR}{dt} = \frac{1}{1+\beta(B+E)} - \rho R$$

We model the RKIP-activated synthesis of let7 miRNA (L) by a Hill function, and the association of let7 with any let-7 target (T) including BACH1 mRNA (B) by mass action kinetics, with linear degradation:

$$\frac{dL}{dt} = \frac{aR^r}{m^r + R^r} - L - cLT$$

We assume that this equation has fast dynamics, so it equilibrates to steady state:

$$L = \frac{aR^r}{(m^r + R^r)(1+cT)}.$$

$$cLT = \frac{aR^r}{m^r + R^r} - L = \frac{aR^r}{m^r + R^r} - \frac{aR^r}{(m^r + R^r)(1+cT)} = \frac{aR^r}{m^r + R^r}\left[1 - \frac{1}{1+cT}\right] = \frac{cT}{1+cT}\frac{aR^r}{m^r + R^r}$$

Next, assume that the same let-7 target is activated by BACH1, approximated by a linear function:

$$T = \alpha B$$

Target loss by association with let-7 will be given by:

$$cLT = \frac{c\alpha B}{1+c\alpha B}\frac{aR^r}{m^r + R^r} = \frac{c\alpha B}{1+c\alpha B}\frac{a}{\left(\frac{m}{R}\right)^r + 1}$$

The steady state level of RKIP can be obtained from the first equation:

$$R = \frac{1}{\rho[1+\beta(B+E)]}$$

From here, target loss by association with let-7 will be given by:

$$cLT = \frac{c\alpha B}{1+c\alpha B}\frac{a}{\left(m\rho[1+\beta(B+E)]\right)^r + 1}$$

This function will depend on BACH1 in a nonmonotone fashion, just like the invasion landscape, see Lee et al., PNAS 111(3):E364-73 (2014).

1.7 Nonmonotonicity by generic incoherent feedforward loops

Imagine that a BACH1 target is regulated by BACH1 by two opposing (activating and repressory) regulatory pathways, which are combined in a multiplicative fashion.

One pathway regulates the target sharply, which we model by a steep Hill function:

$$h(B) = s + (S - s)\frac{H^h}{H^h + B^h}$$

The other pathway regulates the target more gradually, which we model by a linear function:

$$f(B) = \alpha B$$

The expression of the target will be given by the product (like a logical AND gate):

$$f(B)h(B) = \alpha B\left[s + (S - s)\frac{H^h}{H^h + B^h}\right].$$

This function will depend on BACH1 in a nonmonotone fashion, as indicated in main text Figure 6H, just like the invasion landscape.

Supplemental Tables

**Single-guide RNA**	**Sequence (5’ -> 3’)**
Fwd-AAVS1-gRNA	CACCGGGGCCACTAGGGACAGGAT
Rev-AAVS1-gRNA	CCCCGGTGATCCCTGTCCTACAAA
Fwd-BACH1-gRNA	CCTGGCCTACGATTCTTGAG
Rev-BACH1-gRNA	CTCAAGAATCGTAGGCCAGG
**shRNAmir**	**Sequence (5’ -> 3’)**
Anti-BACH1	TTCTGAAACATAATCATCGTTT
Anti-RKIP	TGAATCAAGACCATCCCACGAA

Supplemental Table 1: List of guide RNAs used for AAVS1 CRISPR-Cas9 targeting and BACH1 knockout and shRNA sequences targeting BACH1 and RKIP.
Both guide RNAs were synthesized and annealed for the ligation reaction with BbsI digested eSpCas9(1.1) vector.

Primer name	Sequence (5’ -> 3’)
5’ LP junction forward	AACGGGGCTCAGTCTGAAGAGC
5’ LP junction reverse	TCTCGGCATGGACGAGCTGTACAAGTAA
3’ LP junction forward	CCGCCTCTGGCCCACTGTTTC
3’ LP junction reverse	AATCCATCTTGTTCAATGGCCGATCCCA
5’ LP random Integration forward	TCTCGGCATGGACGAGCTGTACAAGTAA
5’ LP random Integration reverse (for HEK293 cell)	AGTTTACCCCGCGCCACCTTCTCTAG
5’ LP random Integration reverse (for MB231 cell)	TCAGGCCGTGCTTACTAAGGGCC
3’ LP random Integration forward	AATCCATCTTGTTCAATGGCCGATCCCA
3’ LP random Integration reverse	TACGGGGAAAAGGCCTCCAAGGCCTACT

Supplemental Table 2: List of genotyping primers for Land-Pad (LP) insertion.
Primers were diluted with molecular-grade water to a working stock of 10 μM from 100 μM frozen stocks.

Primer name	Sequence (5’ -> 3’)
5’ NF junction forward	AACGGGGCTCAGTCTGAAGAGC
5’ NF junction reverse	TCAAAGTCCTTCTGCCCGTTGCTCA
3’ NF junction forward	CCGCCTCTGGCCCACTGTTTC
3’ NF junction reverse	TCGTCAGGCCTTCGATACCGAC
5’ NF random Integration forward	CTGTCCACCTCATCAGAGTA
5’ NF random Integration reverse	TGTGGTGTAGATGTTCGCGA
3’ NF random Integration forward	TTTACGTCGCCGTCCAGCTC
3’ NF random Integration reverse	ACACAACACCGCCTCGACCA

Supplemental Table 3: List of genotyping primers for Negative-Feedback (NF) circuit exchange. Primers were diluted with molecular-grade water to a working stock of 10 μM from 100 μM frozen stocks.

Probe name	Source	Identifier
EGFP/YFP (Mr04097229_mr)	Thermo Fisher Scientific	*CAT#* 4331182
BACH1 (Hs00230917_m1)	Thermo Fisher Scientific	*CAT#* 4331182
PEBP1 (RKIP) (Hs00831506_g1)	Thermo Fisher Scientific	*CAT#* 4331182
HMOX1 (Hs01110250_m1)	Thermo Fisher Scientific	*CAT#* 4331182
MMP1 (Hs00899658_m1)	Thermo Fisher Scientific	*CAT#* 4331182
CXCR4 (Hs00607978_s1)	Thermo Fisher Scientific	*CAT#* 4331182
CCND1 (Hs00765553_m1)	Thermo Fisher Scientific	*CAT#* 4331182
RPL13A (Hs04194366_g1, VIC)	Thermo Fisher Scientific	*CAT#* 4331182
Human GAPD (GAPDH) Reference	Thermo Fisher Scientific	*CAT#* 4326317E
RNase P (RPPH1) Reference	Thermo Fisher Scientific	*CAT#* 4403326
**Primer**	**Source**	**Sequence/Identifier**
BACH1t qPCR primer forward	Customized	ACTCCAGAACAGCTGGATTGT
BACH1t qPCR primer reverse	Customized	TGGTATGGAACACTTCTTGGCA
Human GAPD (GAPDH) Reference primer set	IDT	Hs.PT.39a.22214836

Supplemental Table 4: List of TaqMan® probes and qPCR primers for gene expression and copy number detection.
GAPDH and RPL13A reference probe was used in gene expression quantitative PCR analysis while RPPH1 reference probe was used in copy number detection quantitative PCR analysis.
