# Supplementary material for: Nonmonotone invasion landscape by noise-aware control of metastasis activator levels
Source: Nat Chem Biol. 2023 May 25;19(7):887–99. doi: 10.1038/s41589-023-01344-z (PMC10299915; doi:10.1038/s41589-023-01344-z)
Supplement: Supplementary file 2 — Reporting Summary [file 41589_2023_1344_MOESM2_ESM.pdf]

nature research

Corresponding author(s): Gábor Balázsi

Last updated by author(s): Mar 10, 2023

Reporting Summary

Nature Research wishes to improve the reproducibility of the work that we publish. This form provides structure for consistency and transparency in reporting. For further information on Nature Research policies, see our Editorial Policies and the Editorial Policy Checklist.

Statistics

For all statistical analyses, confirm that the following items are present in the figure legend, table legend, main text, or Methods section.

n/a	Confirmed	
☐	☒	The exact sample size (n) for each experimental group/condition, given as a discrete number and unit of measurement
☐	☒	A statement on whether measurements were taken from distinct samples or whether the same sample was measured repeatedly
☐	☒	The statistical test(s) used AND whether they are one- or two-sided<br>*Only common tests should be described solely by name; describe more complex techniques in the Methods section.*
☒	☐	A description of all covariates tested
☐	☒	A description of any assumptions or corrections, such as tests of normality and adjustment for multiple comparisons
☐	☒	A full description of the statistical parameters including central tendency (e.g. means) or other basic estimates (e.g. regression coefficient) AND variation (e.g. standard deviation) or associated estimates of uncertainty (e.g. confidence intervals)
☐	☒	For null hypothesis testing, the test statistic (e.g. F, t, r) with confidence intervals, effect sizes, degrees of freedom and P value noted<br>*Give P values as exact values whenever suitable.*
☒	☐	For Bayesian analysis, information on the choice of priors and Markov chain Monte Carlo settings
☒	☐	For hierarchical and complex designs, identification of the appropriate level for tests and full reporting of outcomes
☒	☐	Estimates of effect sizes (e.g. Cohen's d, Pearson's r), indicating how they were calculated

Our web collection on statistics for biologists contains articles on many of the points above.

Software and code

Policy information about availability of computer code

Data collection	Cellometer® Vision CBA Image Cytometer (Nexcelom Bioscience LLC.); Tecan Infinite 200Pro Microplate Reader; Nikon Elements AR v4.40.00 (Build 1084); FACSDiva v9.0 (Becton, Dickinson and Company);
Data analysis	GraphPad Prism 8 (GraphPad Software, Inc); FlowJo_V10 (FlowJo LLC); MATLAB R2020b (MathWorks, Inc.); ImageJ 1.52a; PANTHER_17.0 (web-based interface); cBioPortal_v5.2.8 (web-based tool); SplashRNA_v2; Custom MATLAB code files are available on: https://github.com/gaborbalazsi/BACH1Landscape

For manuscripts utilizing custom algorithms or software that are central to the research but not yet described in published literature, software must be made available to editors and reviewers. We strongly encourage code deposition in a community repository (e.g. GitHub). See the Nature Research guidelines for submitting code & software for further information.

Data

Policy information about availability of data

All manuscripts must include a data availability statement. This statement should provide the following information, where applicable:

- Accession codes, unique identifiers, or web links for publicly available datasets
- A list of figures that have associated raw data
- A description of any restrictions on data availability

The authors declare that all data supporting the findings of this study is available in the article and its supplementary files. Data for the main figures and Extended Data figures are provided in the Source Data files, and supplementary information files for the supplementary figures. Raw data can be accessed as a link from the webpage: https://openwetware.org/wiki/CHIP:Data

April 2020

Field-specific reporting

Please select the one below that is the best fit for your research. If you are not sure, read the appropriate sections before making your selection.

☒ Life sciences ☐ Behavioural & social sciences ☐ Ecological, evolutionary & environmental sciences

For a reference copy of the document with all sections, see nature.com/documents/nr-reporting-summary-flat.pdf

Life sciences study design

All studies must disclose on these points even when the disclosure is negative.

Sample size	All analyses utilized complete experimental or simulated data sets. No sample-size calculation was performed. Each cell type was biologically replicated with two monoclonal populations in all the experimental designs, and with three technical repeats for each experimental operation where applicable. Cell sample size for specific experiments was determined based on the manufacturer's recommendation or published literature's method. For flow cytometry, we analyzed at least 10000 events/sample.
Data exclusions	No data were excluded.
Replication	Every experiment was conducted with n>=3 technical replicates for each individual sample. Biological replicates with two additional independent samples (total n=3) were assessed from both cell types. Gene circuit characteristics were stably reproducible for over a month. Data were repeatable with different attempts.
Randomization	Individual samples were grouped based on the integrated gene circuit type and genetic background. Within the same cell type, samples were distinguished based on monoclonality. In dose-response experiments, each sample was classified into different test groups based on chemical inducer (Dox) levels, and was analyzed and compared accordingly.
Blinding	The study does not contain experiments where blinding would be applicable.

Reporting for specific materials, systems and methods

We require information from authors about some types of materials, experimental systems and methods used in many studies. Here, indicate whether each material, system or method listed is relevant to your study. If you are not sure if a list item applies to your research, read the appropriate section before selecting a response.

Materials & experimental systems

n/a	Involved in the study	
☐	☒	Antibodies
☐	☒	Eukaryotic cell lines
☒	☐	Palaeontology and archaeology
☒	☐	Animals and other organisms
☒	☐	Human research participants
☒	☐	Clinical data
☒	☐	Dual use research of concern

Methods

n/a	Involved in the study	
☒	☐	ChIP-seq
☐	☒	Flow cytometry
☒	☐	MRI-based neuroimaging

Antibodies

Antibodies used	BACH1 Antibody (F-9) Alexa Fluor (Santa Cruz Biotechnology, CAT# sc-271211_AF647, RRID: AB_10608972);<br>BACH1 Antibody (F-9) (Santa Cruz Biotechnology, CAT# sc-271211, RRID: AB_10608972);<br>β-Tubulin (G-8) (Santa Cruz Biotechnology, sc-55529);<br>Goat anti Mouse HRP secondary (Abcam, ab131368)
Validation	For BACH1 Antibody (F-9) Alexa Fluor and BACH1 Antibody (F-9), see: https://datasheets.scbt.com/sc-271211.pdf;<br>For β-Tubulin (G-8), see: https://datasheets.scbt.com/sc-55529.pdf;

Eukaryotic cell lines

Policy information about cell lines

Cell line source(s)	HEK293 and MDA-MB-231 from American Type Culture Collection (ATCC)
Authentication	We authenticated the cell lines by direct source confirmation and phenotypic observation.
Mycoplasma contamination	The cell lines were confirmed to have no mycoplasma contamination.

Commonly misidentified lines (See ICLAC register)	There is no commonly misidentified lines in this research.

Flow Cytometry

Plots

Confirm that:

☒ The axis labels state the marker and fluorochrome used (e.g. CD4-FITC).

☒ The axis scales are clearly visible. Include numbers along axes only for bottom left plot of group (a 'group' is an analysis of identical markers).

☒ All plots are contour plots with outliers or pseudocolor plots.

☒ A numerical value for number of cells or percentage (with statistics) is provided.

Methodology

Sample preparation	Each sample was first washed with 1x PBS twice before trypsinization for 4-5 minutes. Then trypsin was neutralized with corresponding fresh complete culture media. Next, the sample culture was transferred into a flow cytometry tube with strainer cap or into a 96-well plate before flow cytometry measurement.
Instrument	BD LSRFortessa flow cytometer (Becton, Dickinson and Company)
Software	Data collection: FACSDiva v9.0 (Becton, Dickinson and Company);<br>Data analysis: FlowJo_V10 (FlowJo LLC); MATLAB R2020b (MathWorks, Inc.)
Cell population abundance	Each flow cytometry measurement aimed to collect at least 10K viable single cells within the FSC/SSC gate, with a few exceptions in post-immunofluorescence flow cytometry. Cells were washed with PBS and filtered before flow cytometry.
Gating strategy	Preliminary FSC/SSC gates were determined using an individual control sample for each cell type, with assistance from professional technicians. Cell debris and clusters were excluded from data analysis. Fluorescence-based gating was further applied on post-sort populations before analysis. The same gating strategy was applied for all samples measured in a given round. Gates were only slightly shifted between different rounds of measurement due to batch effects. Positive fluorescence boundaries were defined, ensuring that nearly all cells from negative control samples where excluded.

☒ Tick this box to confirm that a figure exemplifying the gating strategy is provided in the Supplementary Information.
