# Supplementary material for: Nonmonotone invasion landscape by noise-aware control of metastasis activator levels
Source: Nat Chem Biol. 2023 May 25;19(7):887–99. doi: 10.1038/s41589-023-01344-z (PMC10299915; doi:10.1038/s41589-023-01344-z)

Ectopic BACH1 Dose response in mNF-BACH1 dose response (right 6 lanes) & mNF-GFP dose response (middle 3 lanes) & native BACH1 in LP, KO, mNF-GFP low-noise with no induction and KO-ctrl (left 4 lanes)

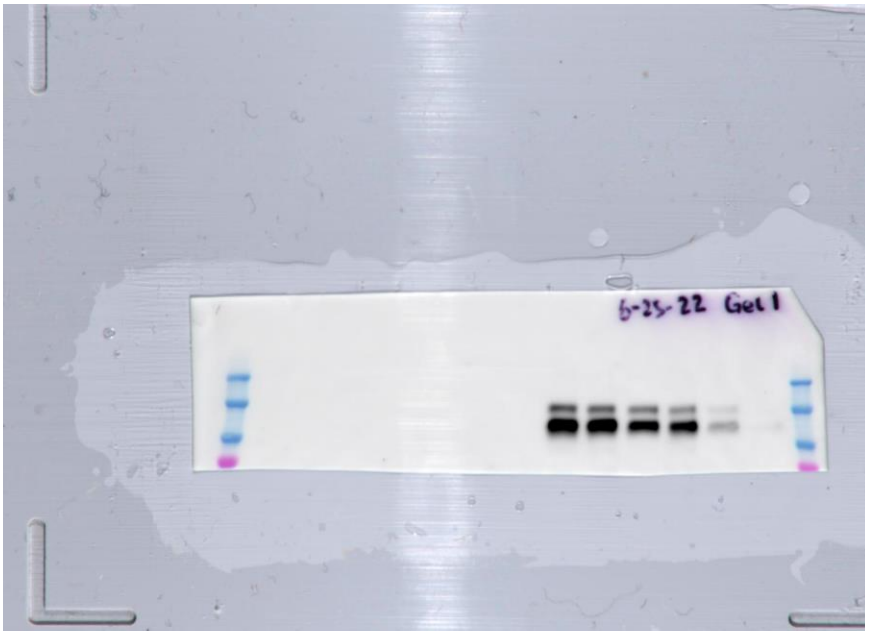

Ectopic BACH1 imaging minimized for Native BACH1

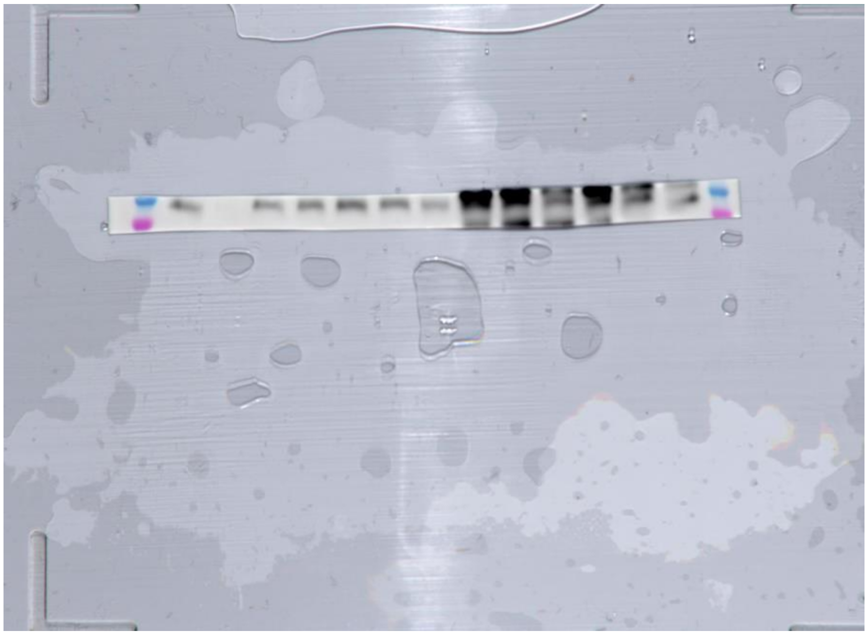

Beta-Tubulin internal reference in the same order

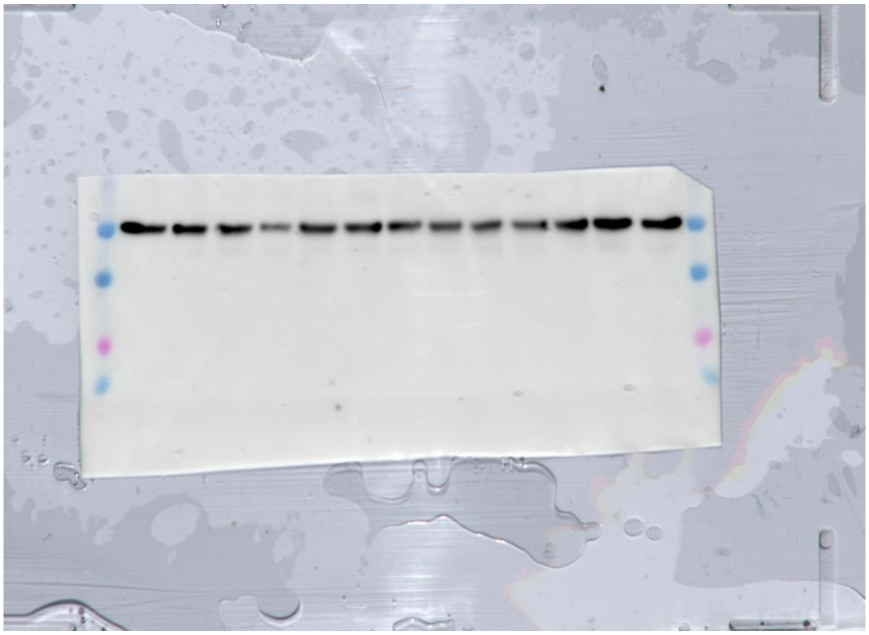

Supplement: Source Data Fig. 2 — Unprocessed western blots. [file 41589_2023_1344_MOESM5_ESM.pdf]
