# Supplementary material for: Nonmonotone invasion landscape by noise-aware control of metastasis activator levels
Source: Nat Chem Biol. 2023 May 25;19(7):887–99. doi: 10.1038/s41589-023-01344-z (PMC10299915; doi:10.1038/s41589-023-01344-z)

Ectopic BACH1 together with Native BACH1

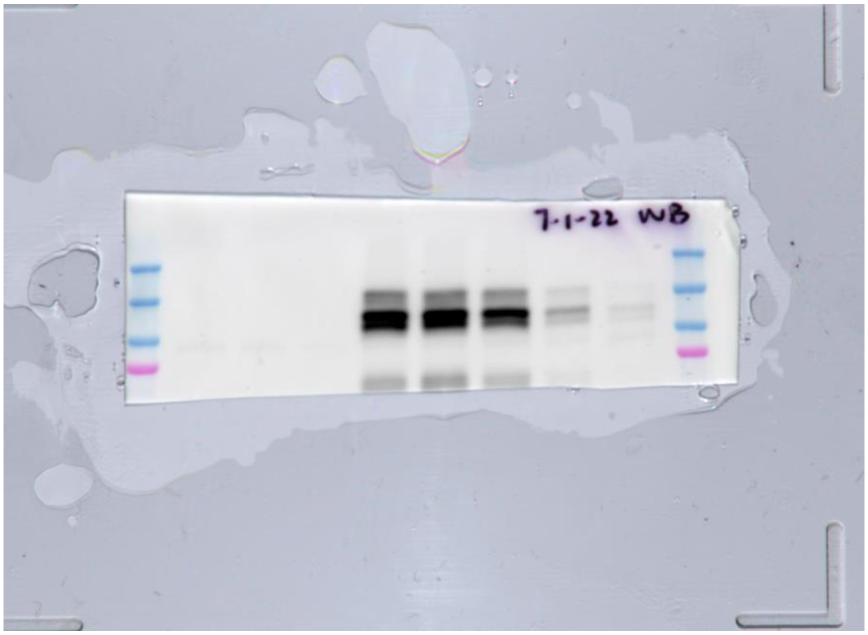


Ectopic BACH1 imaging minimized for Native BACH1

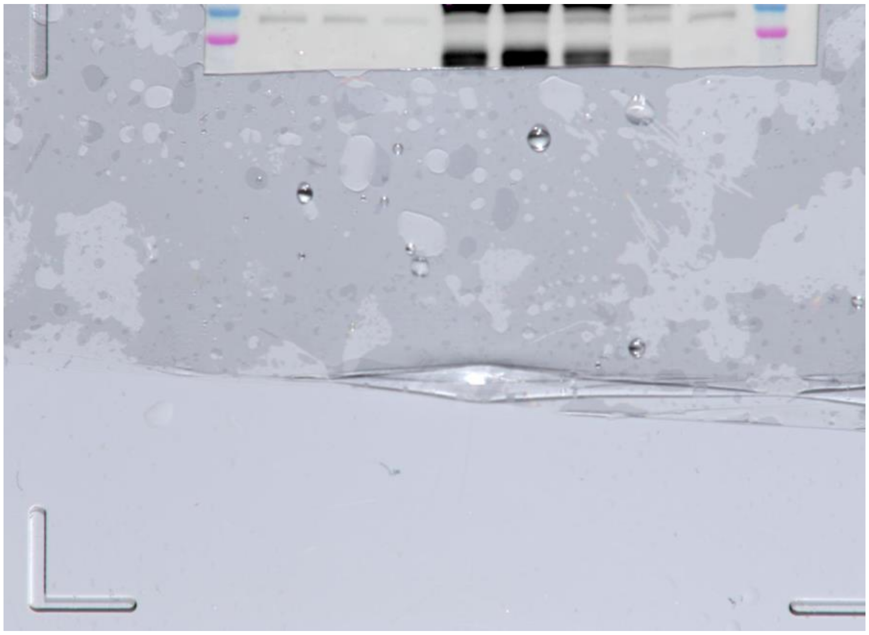

Beta-Tubulin internal reference

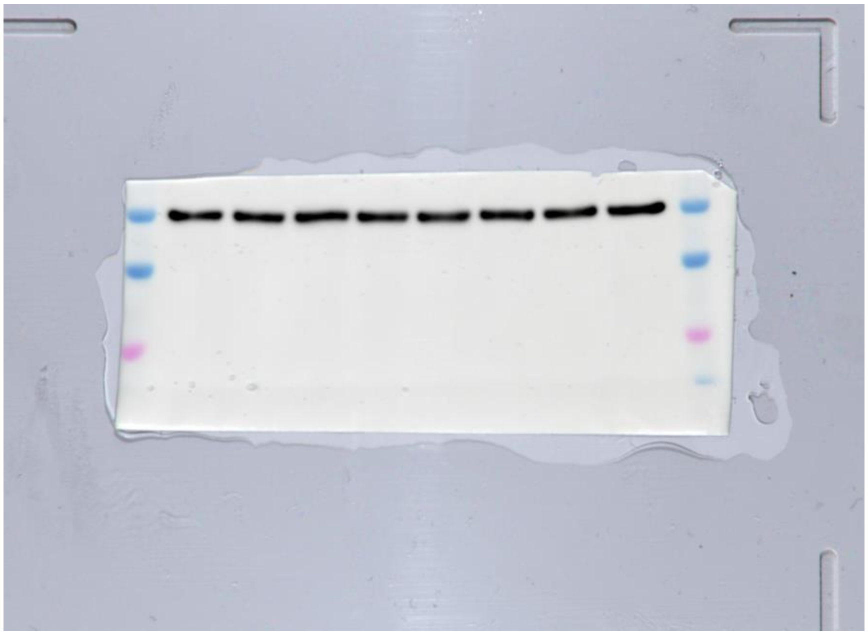

Supplement: Source Data Fig. 5 — Unprocessed western blots. [file 41589_2023_1344_MOESM9_ESM.pdf]
