# Supplementary material for: Nonmonotone invasion landscape by noise-aware control of metastasis activator levels
Source: Nat Chem Biol. 2023 May 25;19(7):887–99. doi: 10.1038/s41589-023-01344-z (PMC10299915; doi:10.1038/s41589-023-01344-z)

MB231 LP clones 3’ LP RI

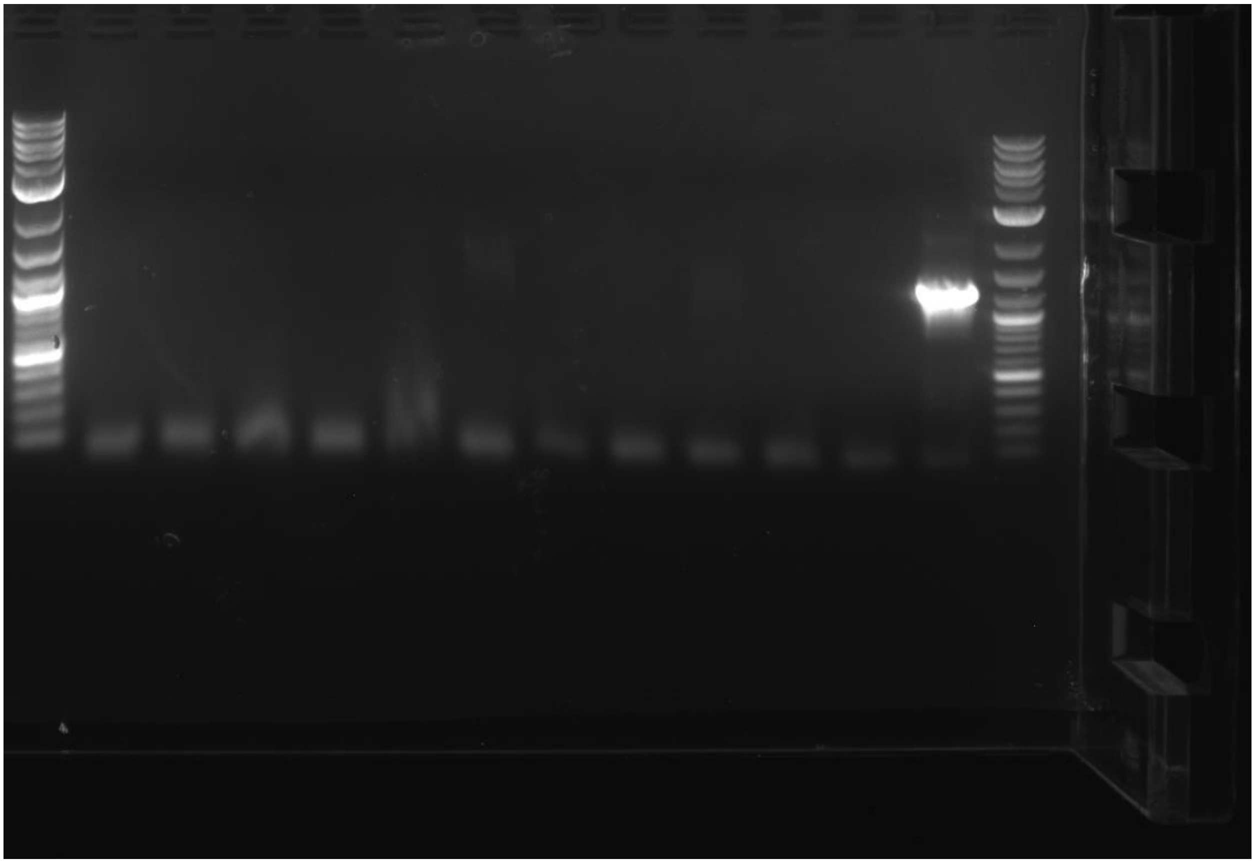

MB231 LP clones 5’ LP JA and 3’ LP JA

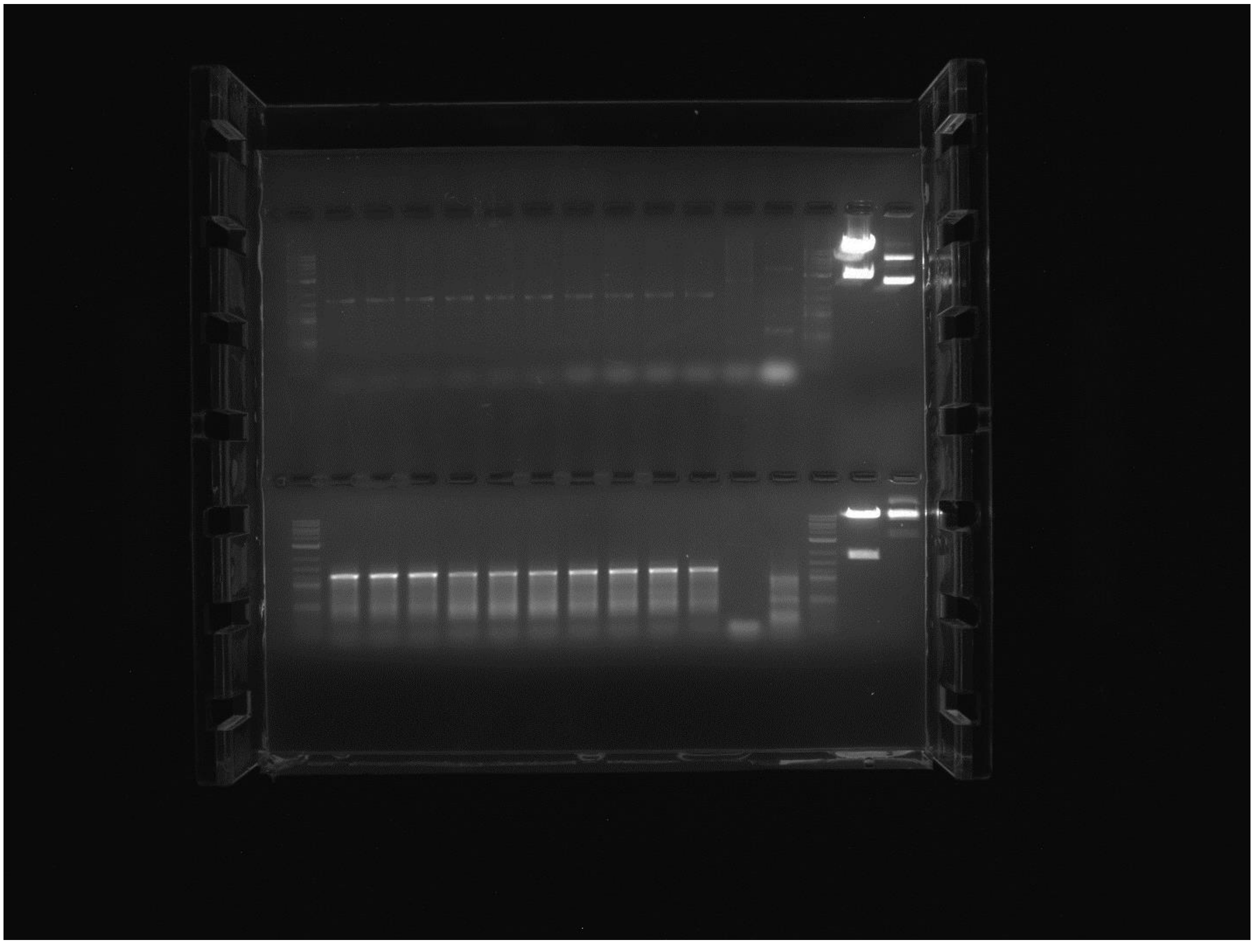

MB231 LP clones 5’ LP RI

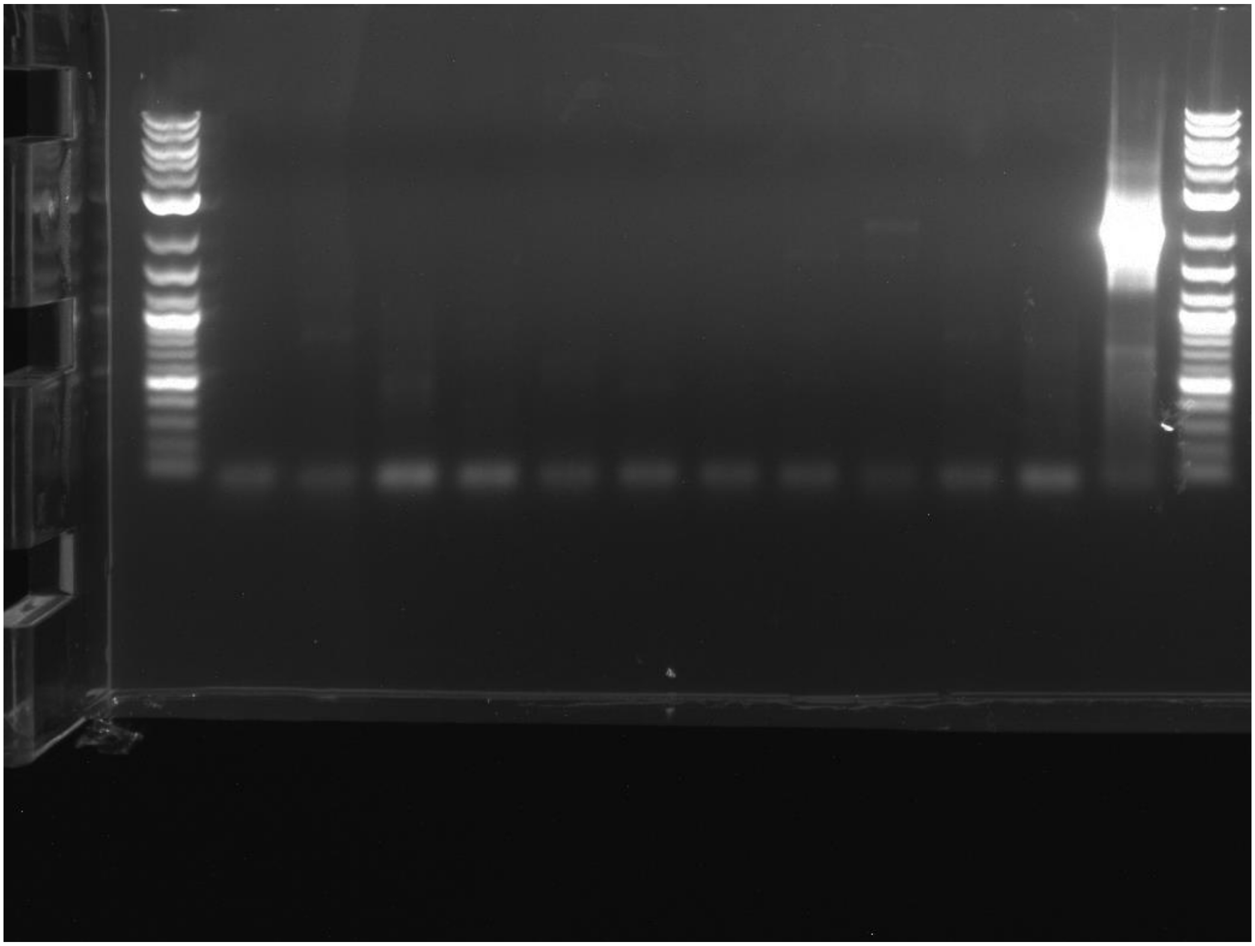

MB231 NF clones 5' and 3’ NF JA

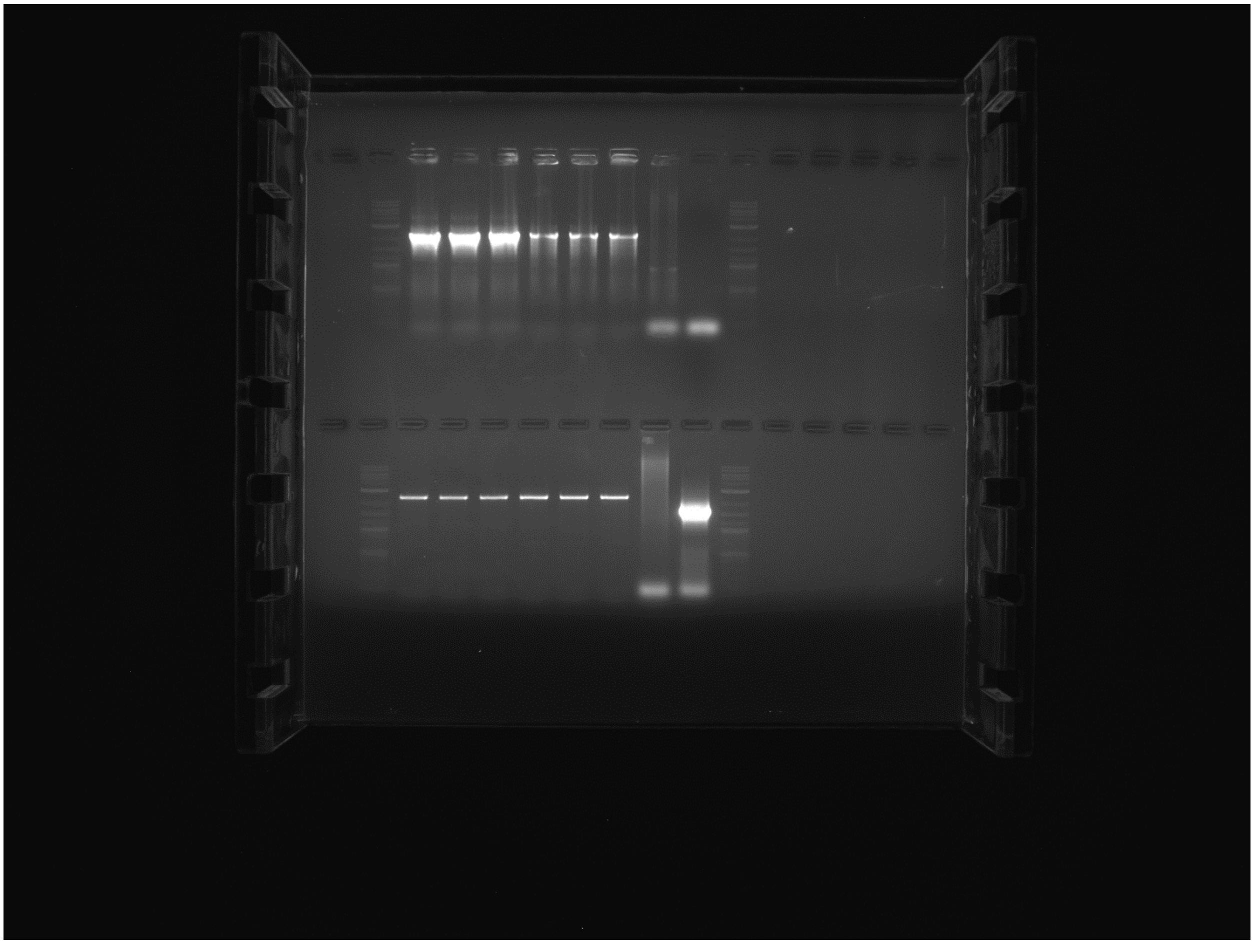

MB231 NF clones 5' and 3’ NF RI

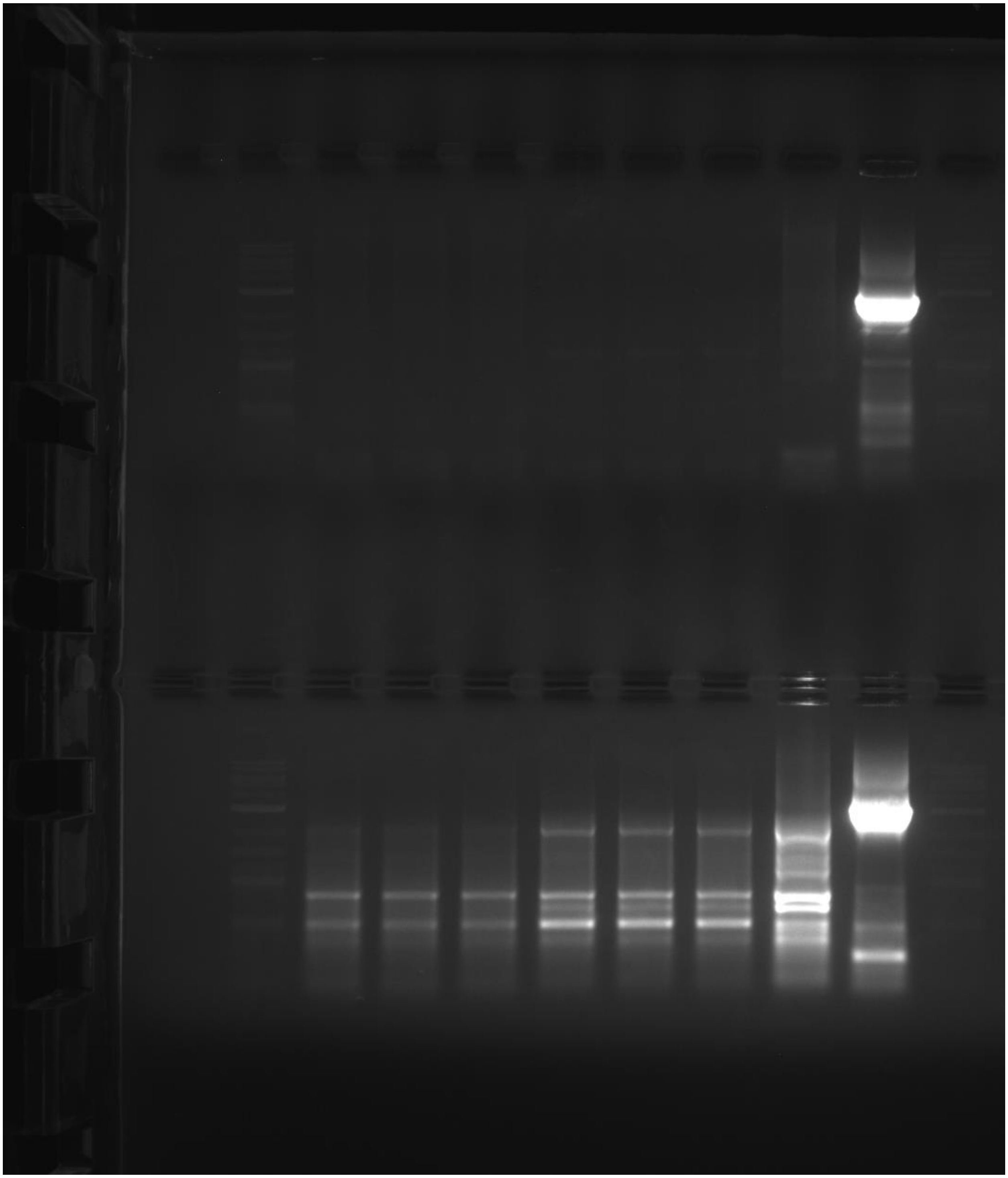

HEK293 LP clones 3 LP JA

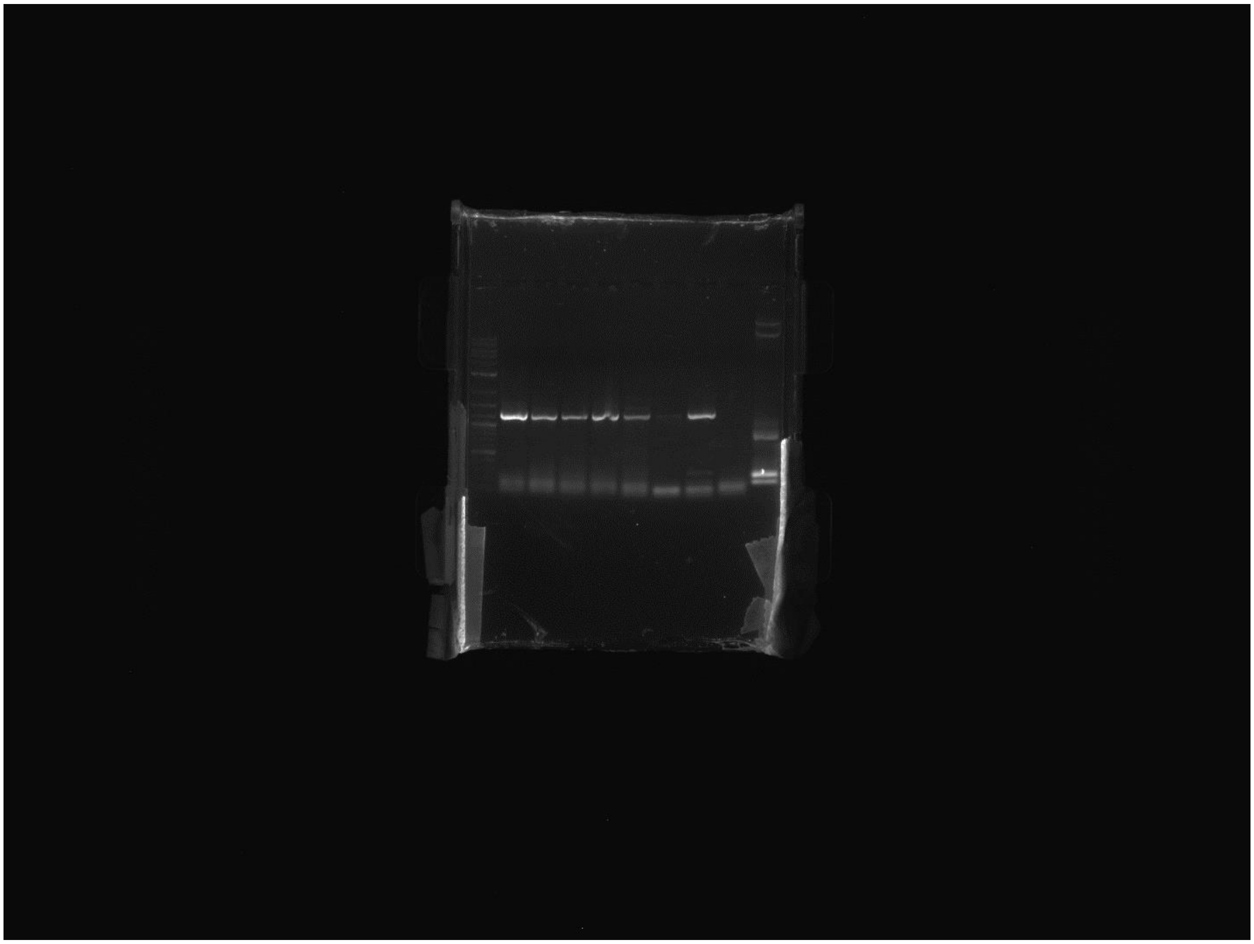

HEK293 LP clones 5’ LP JA

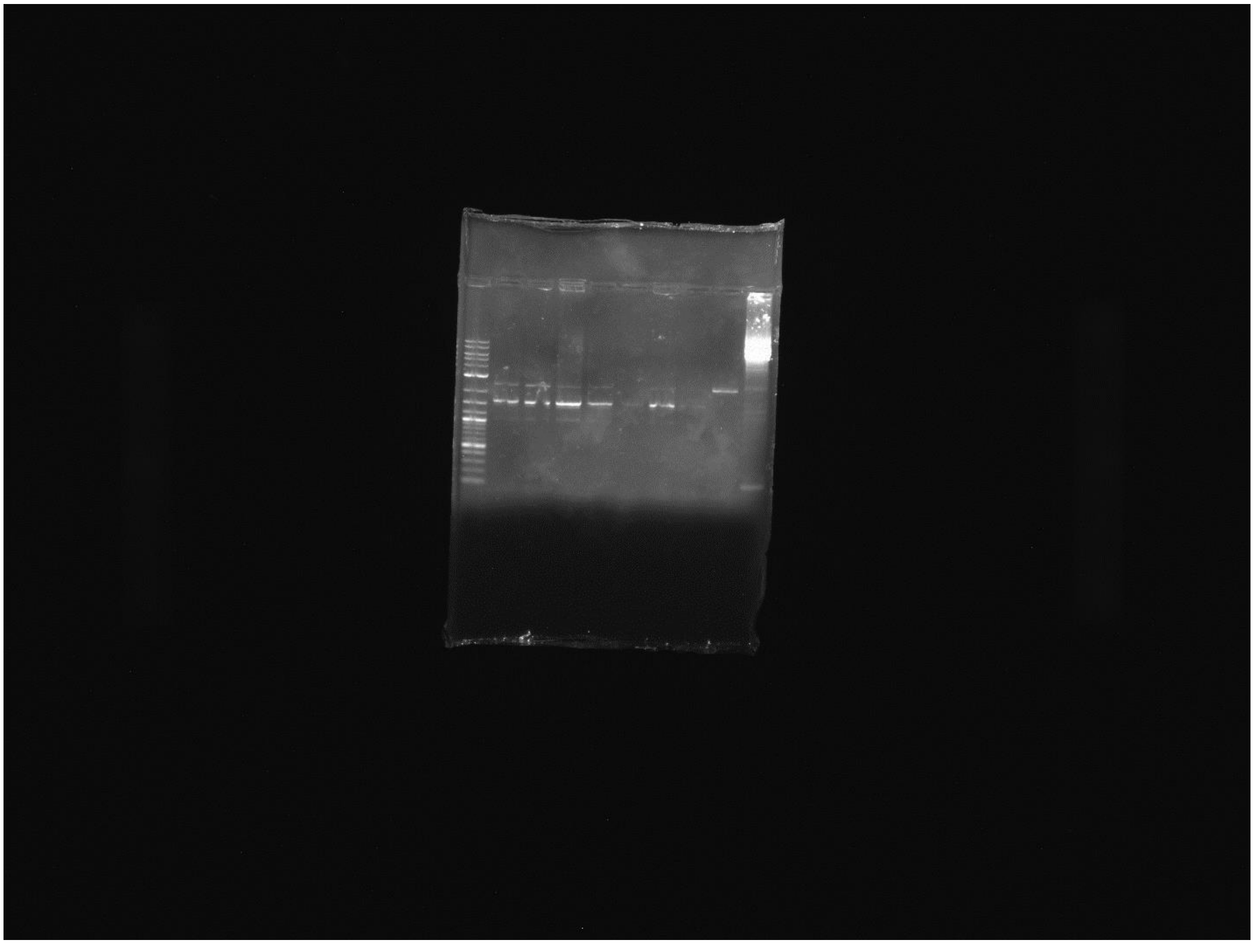

HEK293 NF clones 3' NF RI

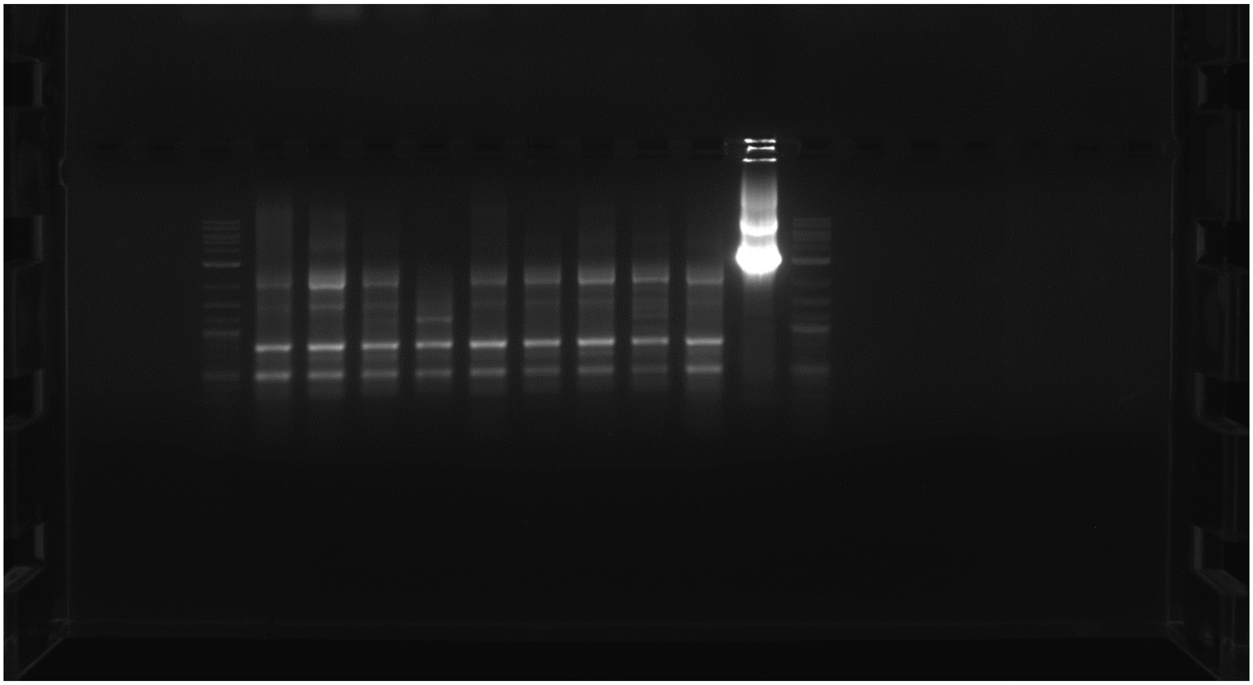

HEK293 NF clones 3’ NF JA

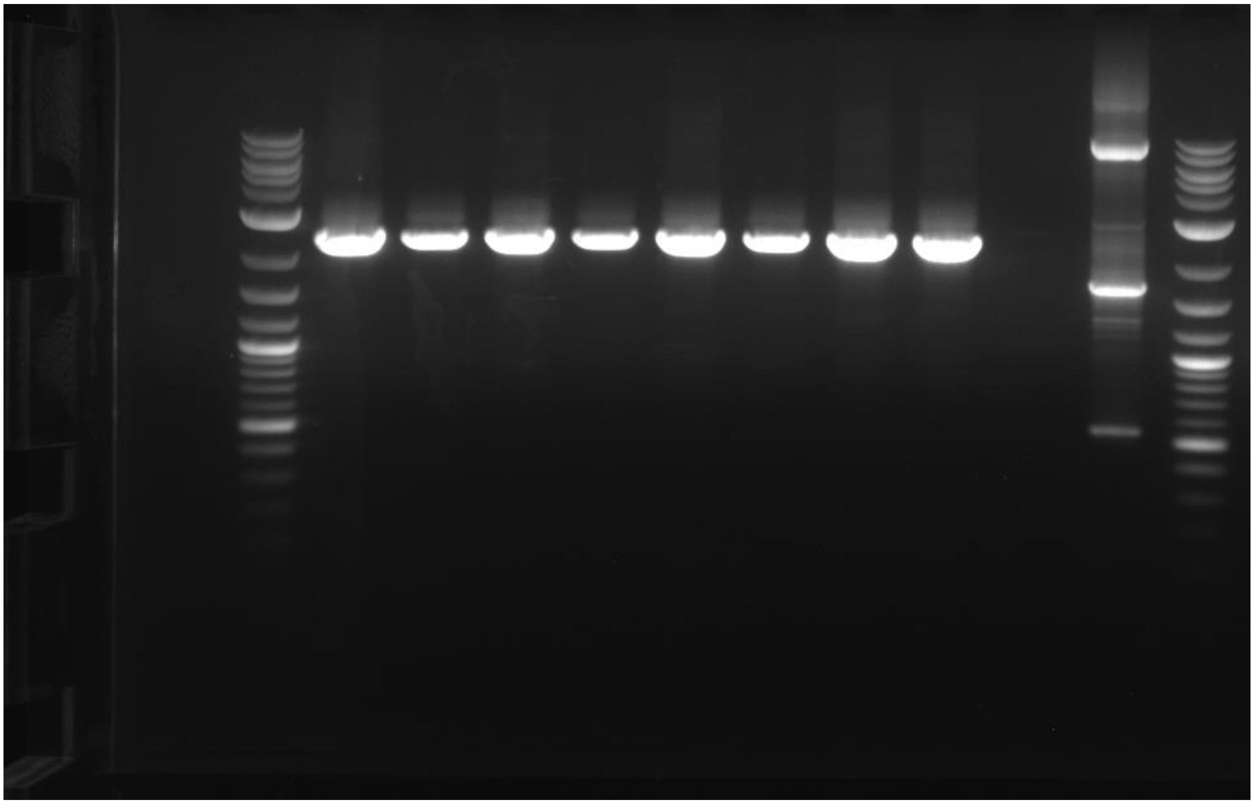

HEK293 NF clones 5' NF RI

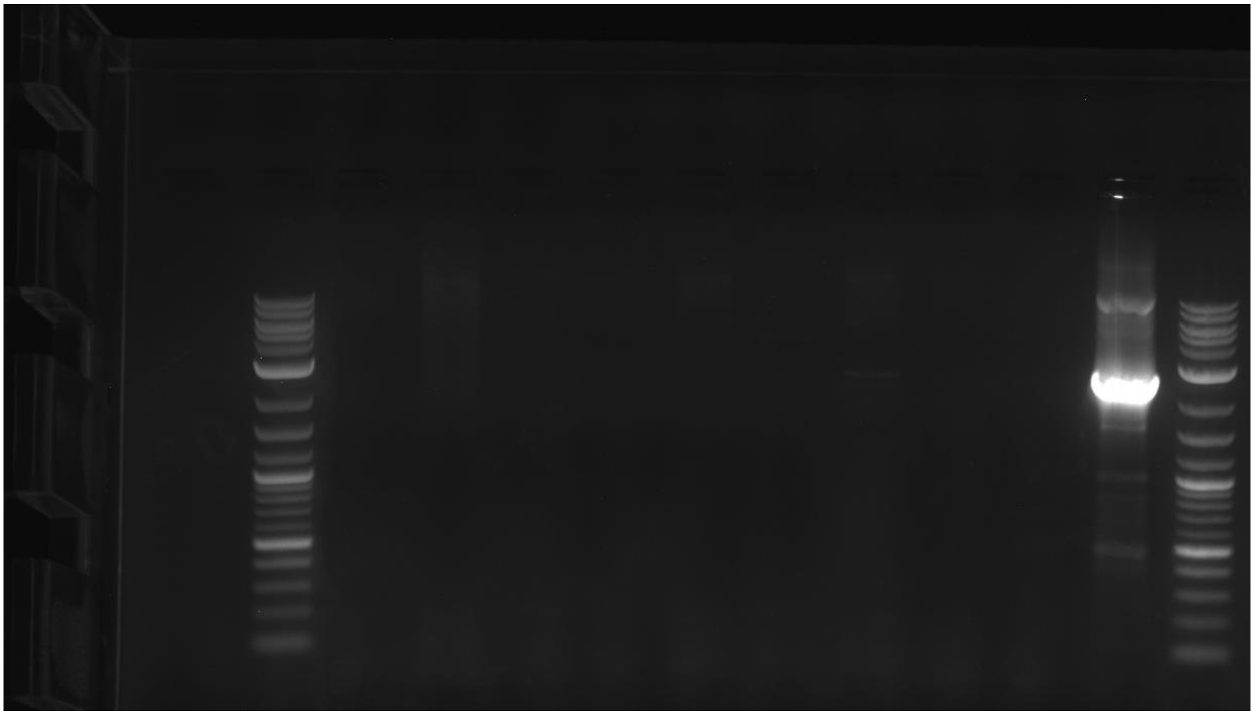

HEK293 NF clones 5’ NF JA

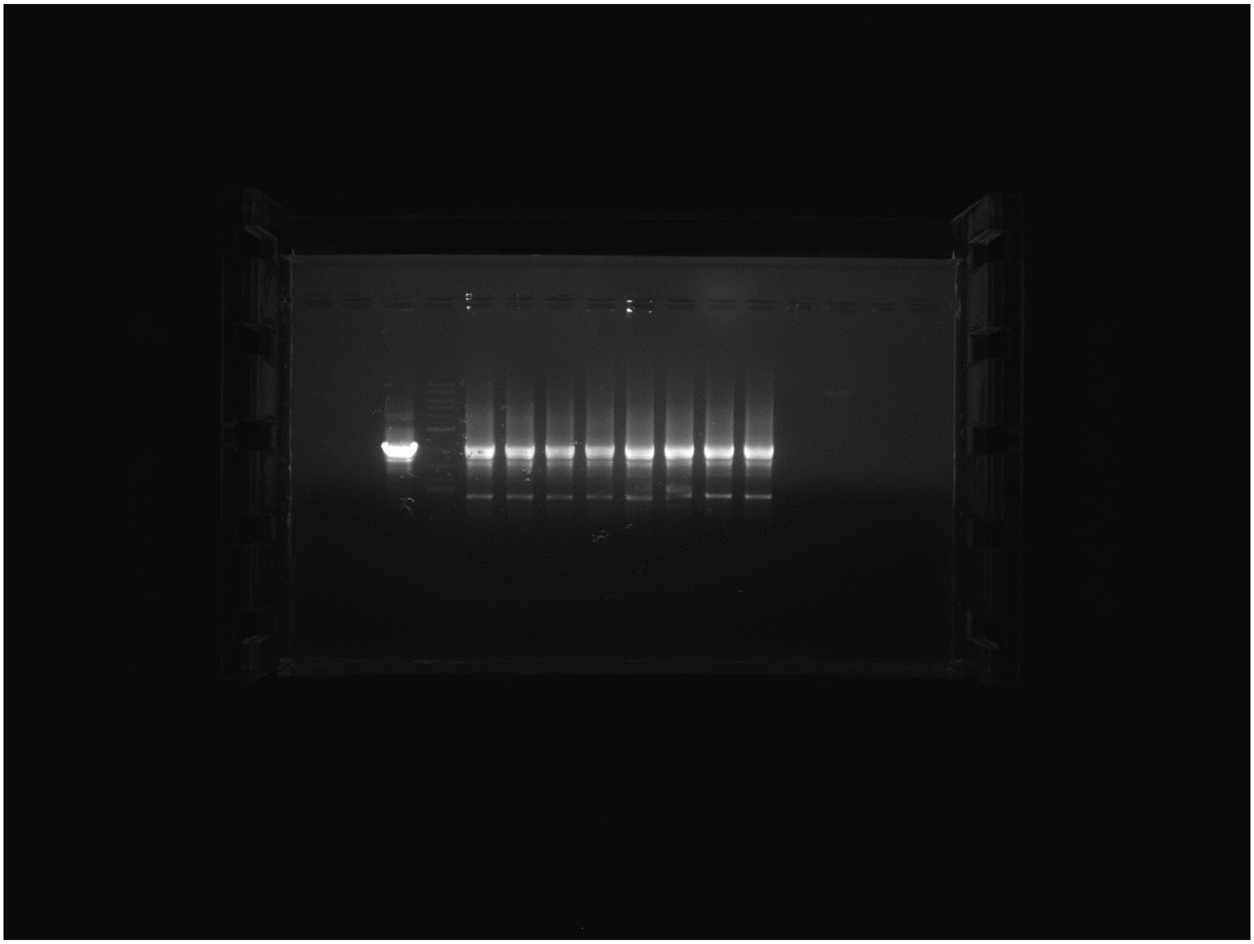

HEK293-LP clones 3' LP RI

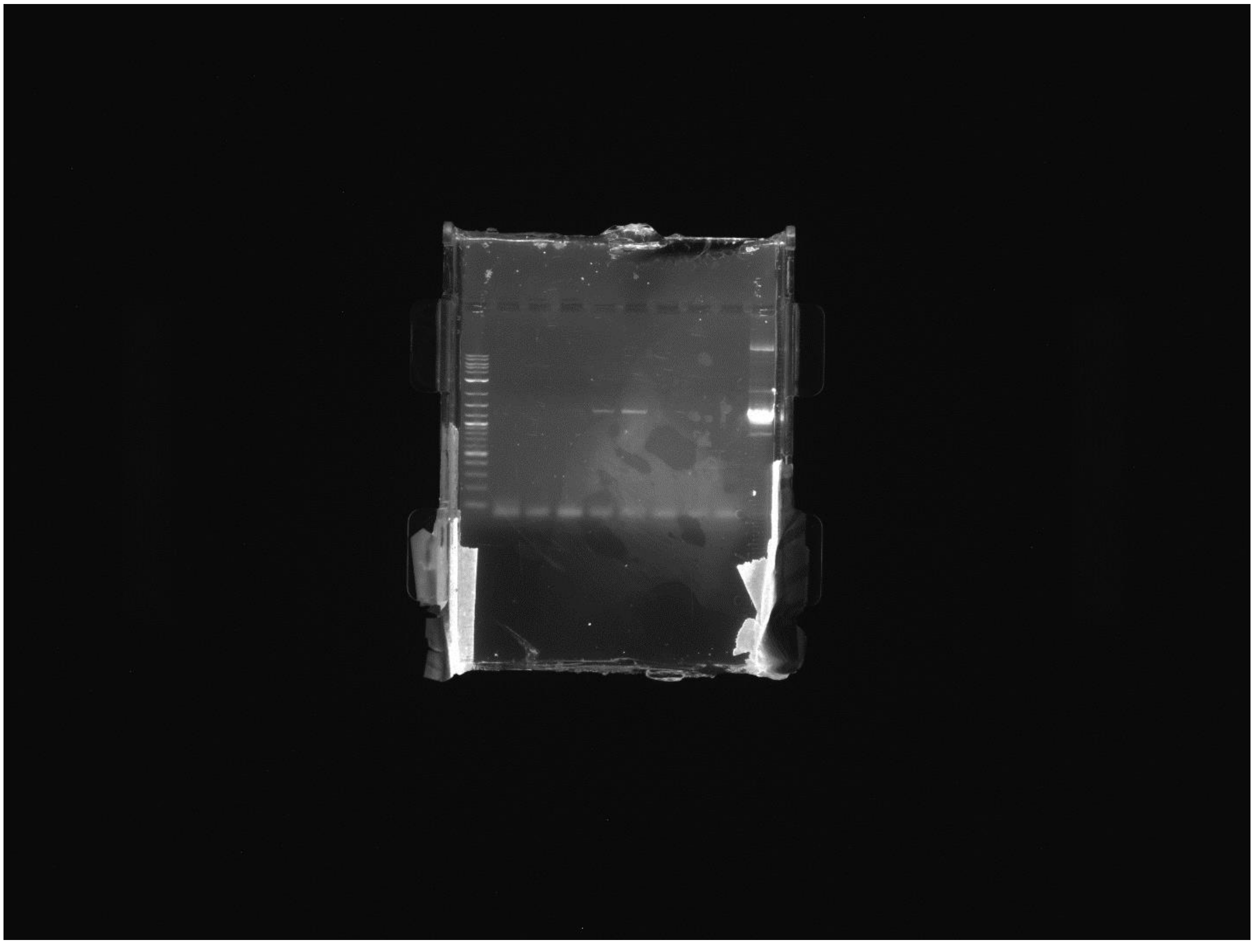

HEK293-LP clones 5' LP RI

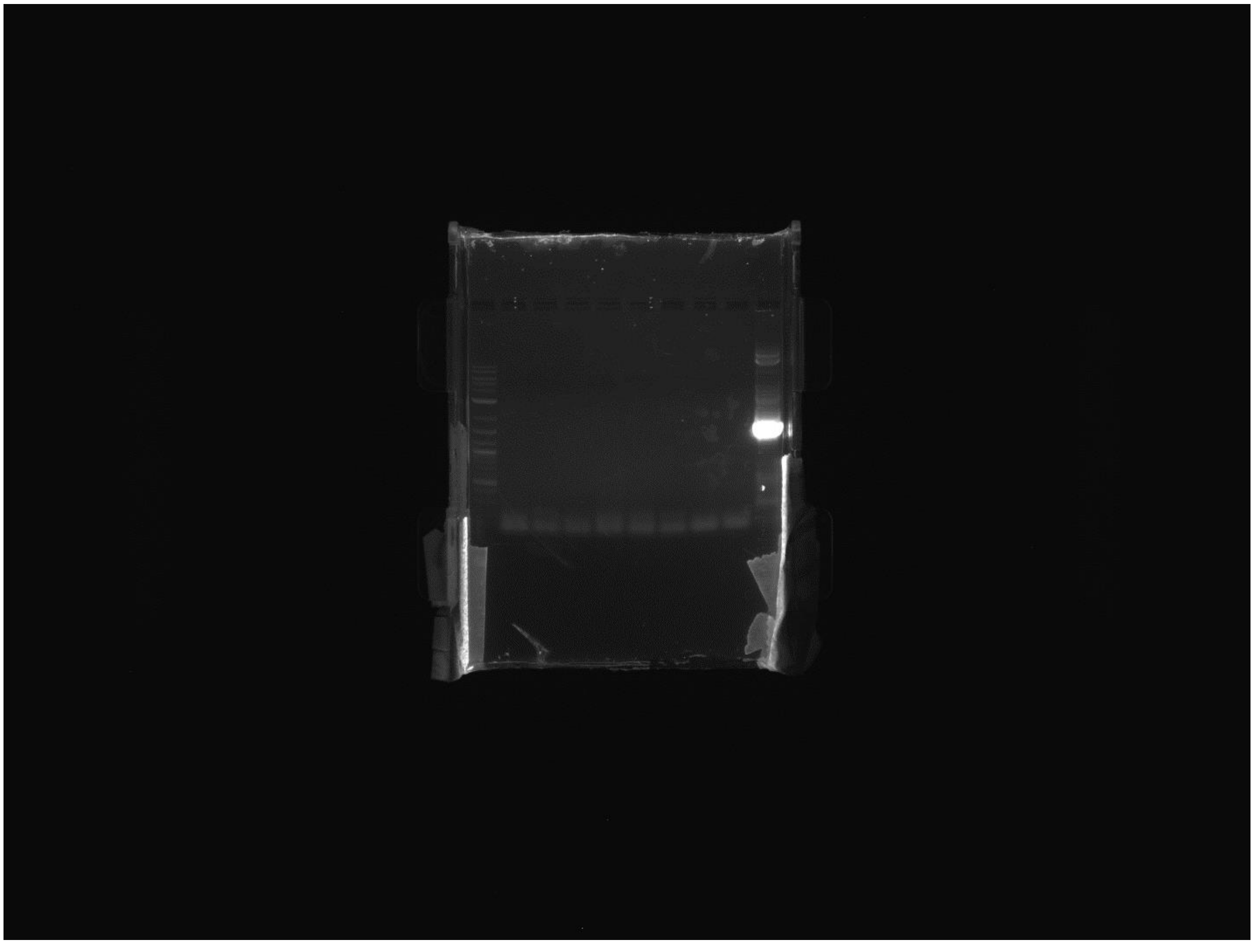

Supplement: Source Data Extended Data Fig. 1 — Unprocessed gels. [file 41589_2023_1344_MOESM12_ESM.pdf]
